# The *Bovidae CSN2 Locus* as a Multilayered Evolutionary System: Evidence from Retroposons, Trans-Species Variation and Ancient Recombination

**DOI:** 10.3390/ani16162482

**Published:** 2026-08-10

**Authors:** Gianfranco Cosenza, Andrea Fulgione, Sara Albarella, Alfredo Pauciullo

**Affiliations:** 1 Department of Agriculture, University of Naples Federico II, 80055 Portici, NA, Italy; giacosen@unina.it (G.C.); andrea.fulgione@unina.it (A.F.); 2 Department of Veterinary Medicine and Animal Production, University of Naples Federico II, 80137 Naples, NA, Italy; 3 Department of Agricultural, Forest and Food Sciences, University of Turin, 10095 Grugliasco, TO, Italy; alfredo.pauciullo@unito.it

**Keywords:** β-casein, retrotransposons, structural variation, trans-species polymorphism, incomplete lineage sorting, recombination

## Abstract

The β-casein gene (*CSN2*) encodes one of the major milk proteins involved in calcium and phosphorus transport and combines a highly conserved genomic organization with extensive genetic diversity, making it an informative model for investigating mammalian genome evolution. Although numerous studies have described *CSN2* polymorphisms, particularly in domestic species, the broader evolutionary history of the *locus* remains poorly understood. Here, we investigated the evolutionary architecture of *CSN2* across *Bovidae* and related *Cetartiodactyla* by integrating coding variation, non-coding polymorphisms, retroposon insertions and recombination analyses. Comparative genomic analyses identified previously undescribed caprine haplotypes, expanded the known spectrum of β-casein variation in domestic and wild *Caprinae*, and revealed multiple structural configurations of the *locus*, including distinct retroposon architectures and intronic insertion/deletion polymorphisms. Retroposon mapping and comparative analyses supported a hierarchical accumulation of structural variants, while also indicating ancestral polymorphism and lineage-specific retention in rapidly radiating caprine lineages. A *locus*-level phylogenetic analysis revealed two deeply divergent allelic lineages and provided evidence of ancient interallelic recombination predating the divergence of *Capra* and *Ovis*. Comparative analyses further showed that a two-amino-acid deletion previously associated mainly with *Caprinae* is more widely distributed across *Bovidae* than previously recognized. Overall, the *CSN2 locus* emerges as a multilayered evolutionary system shaped by structural variation, ancestral polymorphism, incomplete lineage sorting and ancient recombination, illustrating how the integration of coding, structural and retroposon variation can improve *locus*-level evolutionary reconstruction in *Bovidae*.

## Simple Summary

Milk proteins are essential for the growth and development of newborn mammals. One of the most important of these proteins is β-casein, which is encoded by a gene that has been preserved throughout mammalian evolution while also accumulating considerable genetic variation. Because it combines evolutionary conservation with genetic diversity, the encoding gene (*CSN2*) provides a valuable tool for reconstructing relationships among species, especially in domesticated dairy animals that have been influenced by both natural evolution and human breeding. In this study, we compared *CSN2* sequences from domestic and wild members of the cattle, sheep and goat family, as well as from related mammals, to reconstruct the evolutionary history of the gene. We identified previously unknown genetic variants, discovered ancient genetic features shared among different species, and found evidence that the evolutionary history of this gene is more complex than a strictly bifurcating phylogenetic tree. Our results show that ancient structural and allelic variants at the *CSN2 locus* have persisted across multiple speciation events, generating trans-species patterns of variation within *Bovidae*. These findings improve our understanding of mammalian evolution and provide new information that may be useful for the conservation, management, and genetic characterization of domestic and wild ruminant species.

## 1. Introduction

Milk caseins represent one of the most informative molecular systems for investigating mammalian genes evolution and the structural diversification of proteins involved in lactation. Among them, the β-casein encoding gene (*CSN2*) has emerged as a particularly powerful model for evolutionary and comparative genomics because it combines a highly conserved genomic organization with extensive lineage-specific structural and allelic variability [1,2,3]. Beyond their biological role in milk composition, casein genes also provide useful markers for reconstructing mammalian phylogeny and tracing the evolutionary history of domesticated species [2,4].

Comparative genomic studies indicate that casein genes originated before the radiation of extant mammals and evolved from ancestral genes of the secretory calcium-binding phosphoprotein (SCPP) family, initially involved in mineralized tissue formation before being co-opted into lactation biology [2]. This evolutionary transition most likely involved duplication and functional specialization of an ancestral SCPP gene in the mammalian stem lineage, followed by subsequent diversification of the casein gene family before the radiation of extant mammals [1,2]. The occurrence of both calcium-sensitive and κ-like caseins across all living mammals supports an ancestral origin of the casein gene family prior to mammalian diversification [1]. Early evolutionary models proposed that calcium-sensitive caseins evolved through extensive exon shuffling and intragenic duplications from a common ancestral gene [5], whereas later studies refined this interpretation by revealing a more complex history of exon fragmentation, duplication, and structural remodeling within the casein *locus* [1,6].

Among the calcium-sensitive caseins, *CSN2* exhibits the most conserved exon–intron organization across mammals. Unlike *CSN1S1* (αs1-casein gene) and *CSN1S2* (αs2-casein gene), which underwent extensive duplications and rearrangements, *CSN2* experienced relatively limited structural remodelling, with most variability consisting of nucleotide substitutions and localized coding InDels [1]. The gene typically contains nine exons, including a cryptic exon 3 in humans; exon 4 harbours the major phosphorylation sites, whereas exon 7 encodes the hydrophobic core of the mature protein. Owing to this relative structural conservation, *CSN2* has been proposed as a close representative of the ancestral calcium-sensitive casein gene from which other caseins evolved through serial duplications and exon shuffling [1,6].

Structural analyses of mammalian *CSN2* orthologs further suggest that the central portion of the gene evolved through progressive remodelling of an ancestral phosphoserine-rich exon. Modifications of splice sites fragmented this ancestral exon into smaller modular exons that subsequently underwent duplications and rearrangements. In bovines, the resulting modular organization mainly correspond to exons 3–6, encoding the repetitive and highly phosphorylated region of β-casein, whereas part of the ancestral exon persisted as the large exon 7 [1,6]. The conservation of this modular architecture across diverse mammalian lineages (ruminants, camelids, rodents and primates), together with lineage-specific rearrangements in exon number and length, makes *CSN2* an excellent system for investigating molecular evolution and adaptation of milk composition to neonatal physiological requirements.

Beyond its structural conservation, *CSN2* is characterized by extensive allelic diversity in both domestic and wild mammals, particularly within *Bovidae*. In cattle, at least fifteen β-casein variants have been identified. Among these, variants A1 and A2 are the most common worldwide, whereas variant B occurs at lower frequency, together with several rarer allelic forms [7,8,9]. Goat populations exhibit even greater diversity, including strong-expression alleles (A, A1, B, C, C1, D, E and F), null alleles (0 and 01), and recently identified intermediate-expression variants (C2 and F1) [10,11,12,13,14,15,16,17,18]. Substantial polymorphism has also been reported in sheep and buffalo populations. The molecular basis of these variants includes missense substitutions, splice-associated mutations, premature stop codons, and small insertions/deletions affecting the mature protein sequence [12,13,14,15,19,20,21,22,23,24,25]. This diversity makes *CSN2* a highly informative system for studying the relationship among coding evolution, gene regulation, and phylogenetic diversification.

Alongside nucleotide polymorphisms, increasing availability of whole-genome sequences has highlighted the importance of retroposons, particularly SINE and LINE insertions, as robust phylogenetic markers. Because retroposon insertions are effectively irreversible and unlikely to arise independently at identical orthologous positions, shared insertions provide strong evidence of common ancestry [26,27]. Retroposons are especially valuable for resolving rapid radiation events in which sequence-based phylogenies generate unresolved or contradictory trees that are easily refuted by subsequent genomic analyses, as shown in *Cetartiodactyla* evolution [26,28].

Despite extensive characterization of *CSN2* coding polymorphisms, the broader structural evolution of the *locus* across *Bovidae* remains insufficiently explored. Previous studies rarely integrated retroposon architecture, intronic structural polymorphisms, and coding variation within a unified phylogenetic framework. Consequently, the contributions of ancestral polymorphism, lineage-specific retention, retroposon remodelling, and incomplete lineage sorting to the diversification of *CSN2* haplotypes during *bovidae* and *caprine* radiations remains unclear.

In this context, the present study investigates the evolutionary architecture of *CSN2* across *Bovidae* and related *Cetartiodactyla* by integrating retroposon insertions, intronic structural polymorphisms, and coding variation. Combining comparative genomics with structural and phylogenetic analyses, we propose a model of evolutionary dynamics for *CSN2* and hypothesize how ancestral polymorphism, along with lineage-specific retention, retroposon remodelling, and coding diversification, most likely contributed to the emergence of extant bovid diversity, with particular emphasis on rapidly radiating caprine lineages.

## 2. Materials and Methods

### 2.1. Dataset Assembly and Database Curation

*CSN2* genomic and coding sequences were retrieved from NCBI GenBank, RefSeq, Whole Genome Shotgun (WGS) assemblies, and Sequence Read Archive (SRA) datasets. The *Capra hircus CSN2* reference sequence AJ011018.3 was used for coordinate standardization, exon annotation, and comparative variant mapping because it represents a complete genomic reference with experimentally validated exon–intron boundaries and contains the exon 2 nucleotide state (g.3794C) corresponding to the haplotype here designated as *CSN2* A2. This sequence provides a consistent reference framework for positional annotation of caprine *CSN2* variants and for the revised haplotype interpretation proposed in the present study. Homologous sequences were collected from domestic goat breeds, wild Capra species, *Ovis* taxa, representative *Bovidae* lineages, additional Cetartiodactyla species, and selected mammalian outgroups. Accession numbers and genomic information (coordinates, sequence orientation, and taxonomy) are reported in Supplementary Table S1. Taxonomic representation reflects the availability of publicly accessible genomic resources and is therefore uneven across some bovid subfamilies.

BLASTn searches (https://blast.ncbi.nlm.nih.gov/Blast.cgi, last access on 15 June 2026) were performed using complete *CSN2* genomic sequence and selected exonic regions as queries. For taxa lacking annotated *CSN2*, the corresponding genomic regions were identified from publicly available whole-genome SRA datasets using similarity searches against AJ011018.3. Only sequences with complete or near-complete coverage of the *CSN2 locus*, unambiguous exon–intron boundaries, consistent orientation, and sufficient overlap with the reference *locus* were retained for comparative and phylogenetic analyses. All available genomic sequences were manually inspected and compared by pairwise alignment against the reference sequence to verify sequence consistency, exon–intron organization, and the occurrence of polymorphic sites. Whenever multiple independent genomic resources were available for the same species, sequence concordance was evaluated across accessions to minimize the inclusion of assembly or sequencing artefacts. Furthermore, inferred polymorphisms were evaluated within the context of allele-specific haplotypes and retained only when consistently supported by independent genomic resources, whenever available.

### 2.2. Multiple Sequence Alignment and Coordinate Standardization

Sequences were initially aligned using EMBOSS Needle ver 6.6.0 (European Molecular Biology Open Software Suite, Hinxton, Cambridge, UK) and subsequently refined manually using DNAsis-Max ver. 3.0 software (Hitachi Software Engineering, Yokohama, Japan) to optimize exon–intron boundaries, resolve alignment ambiguities surrounding insertion/deletion regions, and preserve coding-frame continuity and orthology of homologous regions.

Coordinates of polymorphic sites were standardized relative to *Capra hircus CSN2* (GenBank AJ011018.3). Coding variants were annotated according to their position within the mature β-casein protein and, where appropriate, relative to the signal peptide cleavage site.

Translated alignments were inspected to verify reading-frame conservation and to identify synonymous substitutions, nonsynonymous substitutions, premature stop codons, and in-frame insertions/deletions.

### 2.3. Identification of Coding Variants and Haplotypes

Polymorphic sites within *CSN2* exons were identified by direct comparison with the caprine reference sequence. Haplotypes were defined according to informative nucleotide combination and compared with previously described *CSN2* alleles.

The ancestral or derived status of selected amino acid states was inferred by comparison with mammalian outgroups. In particular, exon 7 coding sequences were aligned across mammals to evaluate the presence or absence of the two-codon region corresponding to Tyr180 and Pro181 in *Bos taurus* β-casein. Full-length (+2 aa) and truncated (−2 aa) states were scored across *Bovidae* and non-bovid mammalian taxa.

### 2.4. Analysis of Non-Coding Variation

Promoter and intronic regions of *CSN2* were extracted from complete genomic sequences and aligned across representative caprine alleles and related taxa. Single nucleotide substitutions and insertion/deletion variants were recorded relative to AJ011018.3 and classified according to their location within promoter, intronic or untranslated regions.

Allele- and species-associated polymorphism patterns were evaluated across domestic and wild caprine sequences. The 10 bp insertion/deletion polymorphism located in intron 4 (AJ011018.3:g.5292_5301) was scored across all available taxa, including *Capra*, *Ovis* and additional ruminant e non-ruminant lineages.

### 2.5. Retroposon Identification and Annotation

Retroposon insertions at *CSN2* were identified by comparative alignment of orthologous genomic regions. Insertions were annotated by sequence similarity against previously characterized ruminant retroposon families and reference sequences (GenBank accession numbers AC150707.3 and AC150561.6). Retroposon families were assigned based on sequence similarity, insertion length, and characteristic structural patterns. Presence/absence and structural configuration of retroposon insertions were subsequently scored across all taxa included in the phylogenetic analyses.

### 2.6. Phylogenetic Mapping of CSN2 Structural Characters

Retroposon states, intronic insertion/deletion polymorphism and coding variants were mapped onto published, time-calibrated molecular phylogenies of *Cetartiodactyla*, *Bovidae* and *Caprinae* [29,30,31,32].

Temporal references associated with inferred insertion events therefore correspond to divergence-time estimates reported in these studies and do not represent independent molecular dating analyses performed in the present work. Phylogenetically informative markers included the R1–R3 retroposons, the buffalo-specific LINE insertion L1_BT, the 10 bp InDel of intron 4, the Val177Ala substitution, the Tyr180/Pro181 deletion, the signal peptide Leu/Ile polymorphism, and lineage-specific coding insertions. Character-state distributions were interpreted considering ancestral polymorphism, incomplete lineage sorting, lineage-specific retention, and localized structural remodelling. The hierarchical accumulation of retroposon insertions and the evolutionary distribution of the two-amino-acid deletion was inferred by mapping presence/absence patterns onto the currently accepted species phylogeny of *Cetartiodactyla* and *Bovidae*, using a parsimony-based character mapping approach to provide the most parsimonious interpretation of the observed structural character distribution.

### 2.7. Network and Recombination Analyses of Caprine CSN2 Alleles

Phylogenetic networks were reconstructed using the NeighborNet algorithm [33] implemented in SplitsTree v.5 [34] to visualize reticulate relationships among alleles. Because the objective of the analysis was to visualize conflicting phylogenetic signals and reticulate relationships rather than infer a bifurcating phylogeny, bootstrap resampling was not performed. Bootstrap support is primarily intended to assess confidence in bifurcating tree topologies and is therefore of limited interpretative value for exploratory NeighborNet networks designed to represent conflicting evolutionary signals. Instead, network interpretation was based on the concordance between the NeighborNet topology, GARD breakpoint analyses, and the Phi-test for recombination.

Recombination analyses were conducted using GARD (Genetic Algorithm for Recombination Detection; [35]) through the Datamonkey server (https://www.datamonkey.org; Weaver et al., 2018 [36]) implemented with HyPhy ver 2.5 (Kosakovsky Pond et al., 2020 [37]) with default nucleotide substitution models and automatic breakpoint optimization. Putative breakpoints identified by GARD were used to partition the alignment into regions potentially characterized by distinct evolutionary histories. The phylogenetic trees inferred by GARD for each partition were qualitatively compared to assess topological incongruence across segments. The presence of recombination was statistically tested using the Pairwise Homoplasy Index (Phi test) and further evaluated through NeighborNet network reconstruction and GARD partition analysis.

Recombination signals were further evaluated using the pairwise homoplasy index (Phi test; [38]) implemented in SplitsTree. Topological incongruence among GARD-defined partitions was interpreted as evidence of ancient interallelic recombination when supported by concordant NeighborNet structure, Phi-test significance, and breakpoint analyses.

## 3. Results and Discussion

### 3.1. Discovery and Annotation of Genomic Variants: In Silico Analysis of Goat CSN2 Exonic Sequences

Because goats exhibit the highest level of allelic diversity currently described at the *CSN2 locus*, the comparative analyses first focused on caprine coding variation as the basis for the subsequent evolutionary comparisons across *Bovidae*. Although several caprine *CSN2* alleles have been previously described, available information on their underlying polymorphisms remains limited, apart from a number of known exonic and regulatory variants [19,20,39]. To expand this knowledge, publicly available genomic resources were screened for *CSN2* sequences from domestic goats, wild Capra species, and phylogenetically related taxa.

Using the BLAST tool (https://blast.ncbi.nlm.nih.gov/Blast.cgi, access on 15 June 2026), retrieved sequences were all compared with the *Capra hircus CSN2* A reference sequence AJ011018.3. In addition to annotated genome assemblies, homologous *CSN2* sequences were identified in whole-genome SRA datasets from wild goats, including data from the large-scale caprine genomic study [40,41].

Comparison of exonic sequences revealed four polymorphic sites at the *CSN2 locus* in *Capra hircus*. Three had been previously described. These included a AJ011018.3:g.8946T>C transition at nucleotide 404 of exon 7, resulting in p.Val177Ala substitution; a AJ011018.3:g.10562C>T transition at nucleotide 180 of exon 9, located in the 3′UTR; and a AJ011018.3:g.8915C>T transition at position 373 of exon 7, generating a premature stop codon at position 167 [15,18,19]. Among these, the g.8915C>T mutation generates the previously described *CSN2* 01 null allele. As previously demonstrated, the resulting premature stop codon markedly reduces *CSN2* transcript abundance through nonsense-mediated mRNA decay, leading to absence or extremely low levels of β-casein in milk [18]. The g.8946C>T polymorphism distinguished two major allele groups: A-type alleles, carrying Ala177 (*CSN2* A, A1, E, F, F1, 0, 01), and C-type alleles, carrying Val177 (*CSN2* C, C1, C2, D). The g.10562C>T variant characterizes the silent A1 and C1 alleles [19,20].

In addition to these variants, sequence comparison revealed a further exonic variant, consisting of a AJ011018.3:g.3794A>C transversion at position 52 of exon 2. This mutation causes an Ile → Leu amino acid replacement at position 14 of the leader peptide, corresponding to position −2 relative to the signal peptidase cleavage site.

Considering these four polymorphic sites, six distinct allelic haplotypes were identified in *Capra hircus*, corresponding to six distinct alleles: *CSN2* A (g.3794A/g.8915C/g.8946C/g.10562C), *CSN2* 01 (g.3794A/g.8915T/g.8946C/g.10562C), *CSN2* A1 (g.3794C/g.8915C/g.8946C/g.10562T), *CSN2* C1 (g.3794C/g.8915C/g.8946T/g.10562T), *CSN2* C (g.3794A/g.8915C/g.8946T/g.10562C), and *CSN2* A2 (g.3794C/g.8915C/g.8946C/g.10562C). Extension of the analysis to wild *Capra* species identified an additional allelic haplotype (g.3794C/g.8915C/g.8946T/g.10562C), which was not detected in *Capra hircus* and is here designated as *CSN2* CX (Supplementary Table S2).

The presence of cytosine at exon 2 characterizes not only the allele here proposed as *CSN2* A2 (GeneBank AJ011018.3), introduced to avoid nomenclature ambiguity with the previously described *CSN2* A allele [10] (GeneBank AH001195.2), but also the *CSN2* CX, C1, and A1 allelic sequences. In contrast, this nucleotide state is absent in the *CSN2* C allele (Supplementary Table S2), which has so far been considered as the ancestral form from which the C1 allele originated through a subsequent C>T transition at nucleotide 180 of exon 9 (UTR) [20].

However, this interpretation was based on the polymorphisms known at that time and does not take into account the additional exon 2 polymorphism identified in the present study, which supports a more complex evolutionary history of the C and C1 alleles.

Notably, although additional caprine *CSN2* alleles (B, C2, D, E, F and F1) have previously been described in the literature [13,14,15,16], the mutations responsible for these alleles are currently undocumented in the public genomic databases screened in the present study. However, two silent mutations were identified in exon 7: AJ011018.3:g.8572T>C in Tibetan goat sequences (JBKFXW010000026.1; JBJNUV010000026.1, *CSN2* C1) and AJ011018.3:g.8813C>T in a Nubian goat sequence (JBKFYD010000026.1, *CSN2* A).

### 3.2. Non-Coding Variations at the Goat CSN2 Locus

To investigate additional sources of inter- and intra-species genetic variability at the *CSN2 locus*, promoter and intronic regions were compared across representative caprine alleles, including the major *CSN2* lineages within the *genus Capra*, and sheep (*Ovis aries*) included for inter-genus comparison (Supplementary Table S3).

At least 141 polymorphic sites were identified in non-coding regions of *Capra hircus*, including 113 single nucleotide substitutions and 28 single or multiple nucleotide insertions/deletions. Fourteen polymorphisms are located in the promoter, whereas the remaining variants were distributed across introns (Supplementary Table S3).

Analysis of non-coding regions further identified several allele-associated patterns. In particular, the *CSN2* 01 and *CSN2* A alleles display multiple unique polymorphisms in promoters and introns, enabling discrimination based solely on non-coding variation. By contrast, no strictly allele-specific non-coding mutations were detected for the *CSN2* A1, A2, C, or C1 alleles, which largely shared regulatory and intronic variants. Thus, non-coding polymorphisms provide additional discriminatory power primarily for the *CSN2* A allele and the null allele *CSN2* 01, whereas discrimination among the remaining alleles relies mainly on coding-region polymorphisms.

Extension of the analysis to wild *Capra* species (*Capra falconeri*, *Capra aegagrus*, and *Capra ibex*/*sibirica*) identifies 16 additional SNPs, most of which were shared between *Capra falconeri* (*CSN2* A2 genotype) and *Capra ibex*/*sibirica* (*CSN2* CX genotype) (Supplementary Table S3). This pattern supports the persistence of shared non-coding variation across species boundaries and highlights the close evolutionary relationships among caprine *CSN2* alleles.

### 3.3. Retroposon Architecture of the Goat CSN2 Locus

Retrotransposons, especially LINEs and SINEs, are major components of ruminant genomes. In particular, Bov-B LINEs and their associated SINE families (e.g., Bov-A2 and Bov-tA) are particularly abundant. Their insertions are typically irreversible and rarely undergo precise excision, thus their presence or absence at orthologous *loci* provides highly reliable markers for phylogenetic inference and evolutionary reconstruction within *Ruminantia* [42].

Genes encoding calcium-sensitive caseins (*CSN2*, *CSN1S1*, *CSN1S2*) are a well-characterized example of lineage-specific retrotransposon accumulation. Comparative analyses across ruminant species indicate that several retroposon insertions at these *loci* are shared among taxa and likely reflect ancestral insertion events, whereas others appear to be clade- or species-specific [6,43,44,45,46,47,48,49,50,51].

The present analysis revealed at least four retrotransposon insertions within the promoter region and intron 4 of goat *CSN2*. The promoter-associated element (R0; AJ011018.1, positions 1044–1163) corresponds to a Bov-tA retroposon, whereas three intron 4 insertions (R1, R2, and R3) show high sequence similarity to Bov-A (positions 4785–4909), Bov-A2 (5531–5809), and Bov-B (5890–6480) elements, respectively, with pairwise nucleotide identities ranging from approximately 75% to 90% relative to reference sequences (GenBank AC150707.3; AC150561.6).

More broadly, retrotransposon insertions at the *CSN2* contribute to genomic diversification among ruminants and provide useful markers for phylogenetic reconstruction, population genetic analyses, and traceability studies aimed at fraud detection in dairy and meat products. Overall, the proposed hierarchical accumulation model represents the most parsimonious evolutionary interpretation of the observed retroposon distribution based on the currently available genomic evidence. Although this framework is supported by the congruence between retroposon architecture and the accepted species phylogeny, additional genomic resources from currently underrepresented lineages may allow formal evaluation of alternative evolutionary scenarios in future studies.

### 3.4. Presence–Absence of Two Amino Acids in β-Casein Across Mammals

Comparative analysis of exon 7 revealed a short insertion/deletion polymorphism corresponding to two codons in the mature β-casein protein. Most mammals retain the full-length sequence, whereas many members of the subfamily *Caprinae*, including *Capra hircus* and *Ovis aries*, lack the two amino acids, corresponding to residues Tyr180 and Pro181 in the mature bovine β-casein.

At the nucleotide level, this difference corresponds to a 6 bp deletion that removes two consecutive codons (TATCCC in *Bos taurus*) without disrupting the reading frame and resulting in a mature protein of 207 amino acids in goat and sheep versus 209 in cattle. This structural difference between bovine and ovine–caprine β-caseins has been previously documented in comparative studies of milk proteins [52]. However, the expanded taxonomic comparison performed here demonstrates that this deletion is not restricted to *Caprinae*. It also occurs in several additional bovid lineages, including representatives of *Reduncinae*, *Cephalophinae*, *Alcelaphinae*, and *Hippotraginae*. Notably, the same deletion is also observed in several *Antilopinae* lineages (e.g., *Saiga tatarica*, *Litocranius walleri*, *Eudorcas thomsonii*, *Nanger dama*), whereas other antelopes such as *Aepyceros melampus* and *Neotragus moschatus* retain the full-length (+2 aa) state (Supplementary Figure S1). To our knowledge, this broader and heterogeneous distribution in all these taxa has not been previously reported.

Notably, an additional lineage-specific coding structural variant was also identified in *Syncerus caffer* that carries a triplet insertion (ATT) encoding isoleucine at position 182 of the mature β-casein protein. Beyond *Bovidae*, further structural variation was observed in *Hippopotamidae* [53] that exhibit a unique 12 bp insertion (CCACAGCCCGTT) encoding a tetra peptide (Pro^165^Gln^166^Pro^167^Val^168^) in the mature β-casein (Supplementary Figure S1). These insertions, distinct from both the −2 aa deletion, further highlights the structural plasticity of the *CSN2* coding region across mammalian evolution.

Taken together, these observations indicate that the phylogenetic distribution of the −2 aa InDel across *Bovidae* is broader and more heterogeneous than previously recognized. Rather than representing a feature restricted to ovine and caprine species, the available evidence suggests that the deletion most likely originated as a single derived structural event early during the diversification of *Bovidae* and was subsequently shaped by lineage-specific retention, secondary loss, and incomplete lineage sorting. Although alternative evolutionary scenarios, including multiple independent deletion events, cannot be excluded, they require additional assumptions and therefore appear less parsimonious than a single ancestral origin.

The coexistence of both +2 aa and −2 aa states within *Antilopinae* highlights a mosaic distribution of the deletion that does not strictly follow current species relationships. Within this subfamily, closely related taxa may differ in deletion state, indicating that the character has not been uniformly fixed during diversification. This pattern is consistent with differential retention of ancestral variation during rapid caprine diversification rather than requiring repeated independent origin of identical deletions. Because the deletion is in-frame and occurs within a proline-rich region of the protein, which generally contributes to local conformational flexibility without necessarily disrupting the overall protein architecture, it is unlikely to have caused major functional disruption of β-casein, suggesting that it represents a tolerated structural variation. The deleted region is located outside the major phosphorylation cluster of β-casein and does not alter the reading frame. Moreover, the long-term persistence of both the full-length (+2 aa) and truncated (−2 aa) forms across diverse mammalian lineages suggests that both structural configurations are compatible with normal β-casein function. Nevertheless, subtle effects on local protein conformation, casein micelle assembly, or other technological and nutritional properties associated with β-casein cannot be excluded and will require dedicated biochemical and functional studies. Importantly, the phylogenetic distribution of the −2 aa deletion only partially overlaps that of other structural markers at the *CSN2 locus*, including retroposon insertions (R1–R3) and the intron 4 insertion/deletion polymorphism. This partial concordance indicates that these characters capture different evolutionary processes and timescales, with the coding deletion complementing rather than duplicating the phylogenetic information provided by the structural markers.

In this framework, the −2 aa deletion does not behave as a simple clade-defining synapomorphy, but it seems to act as a structural marker reflecting the combined effect of ancestral polymorphism, lineage-specific retention, and secondary loss. This multi-layered signal reinforces the inference of hierarchical accumulation of rare genomic changes while simultaneously capturing non-tree-like evolutionary processes within *Bovidae*.

### 3.5. Trans-Species Polymorphisms and Ancestral Haplotypes at the CSN2 Locus

Alanine at position 177 of the mature β-casein has traditionally been considered ancestral in goat *CSN2* because it is shared with other ruminants, including cattle, sheep, and buffalo [20]. However, broader mammalian comparison indicates that Val177 is widespread among non-ruminants, including *cetaceans*, *perissodactyls*, *suids*, *carnivores*, and *primates* (Supplementary Figure S1). This distribution is consistent with Val177 representing the ancestral mammalian condition, whereas the g.8946T>C substitution (p.Val177Ala) most likely arose during early ruminant evolution.

A comparable pattern was observed for the g.3794A>C in exon 2. Cytosine at this position, encoding Leu at position −2 of the signal peptide, is conserved across most placental mammals and is therefore likely the ancestral (Supplementary Figure S2). Consistent with this interpretation, the signal peptide of β-casein is highly conserved, and leucine at position −2 is also retained in homologous leader peptides of related casein genes *CSN1S1* and *CSN1S2* in most mammals [1]. Likewise, cytosine at position g.10562 in the 3′UTR appears to be evolutionarily conserved [19].

These observations suggest that the ancestral *CSN2* haplotype likely corresponded to g.3794C/g.8946T/g.10562C, encoding the residues Leu-2/Val177 and carrying cytosine in the 3′UTR. Although this haplotype was not detected in domestic goats, it occurs in wild *Capra* species, such as *Capra sibirica* and *Capra ibex*, and appears widespread among *Artiodactyla*, supporting its ancestral origin.

**Figure 1:**
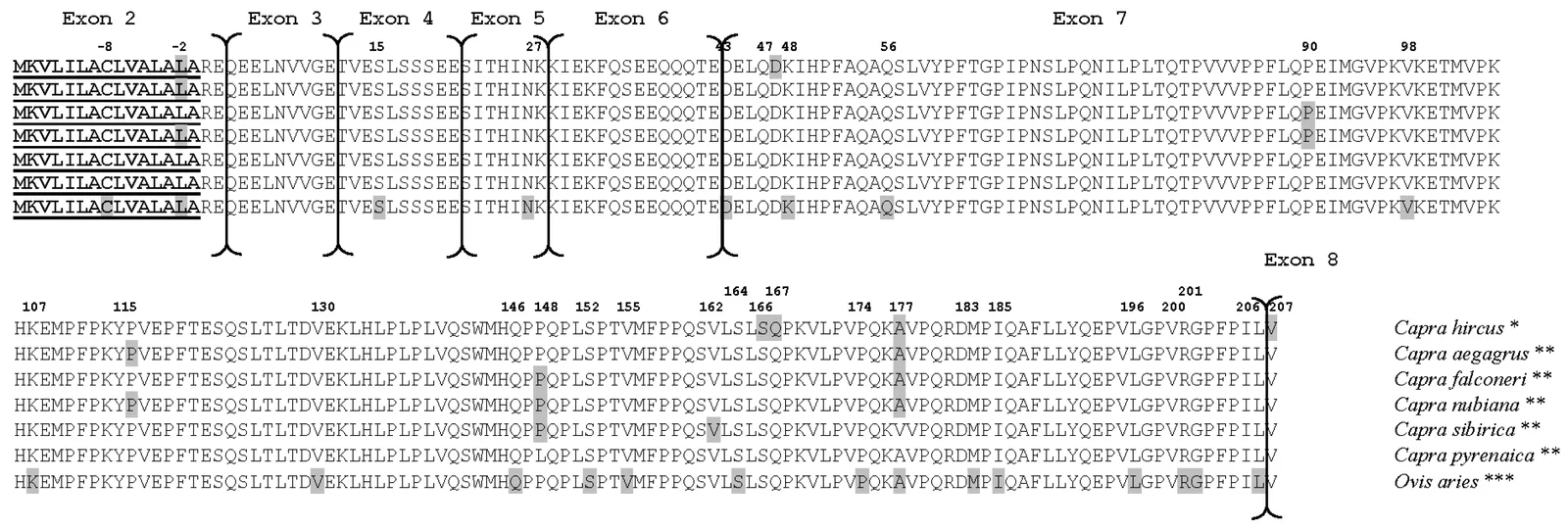
Comparative alignment of deduced β-casein amino acid sequences across representative species of the *genus Capra* and *Ovis aries*. Exon boundaries are indicated above the alignment. The signal peptide region is shown in bold and underlined. Amino acid numbering refers to the mature β-casein protein, whereas negative numbering indicates residues within the signal peptide relative to the signal peptidase cleavage site. Grey shading highlights polymorphic amino acid positions relative to *Capra hircus*: p.Leu–2Ile, p.Asp47Tyr, p.Ser166Tyr, p.Gln167Stop, p.Ala177Val, and p.Val207Asn; *Capra aegagrus*: p.Leu–2Ile, p.Pro115Ser, and p.Ala177Val; *Capra falconeri*: synonymous substitution at codon 90, p.Pro148Leu, and p.Ala177Val; *Capra nubiana*: p.Leu–2Ile, synonymous substitution at codon 90, p.Pro115Ser, p.Pro148Leu, and p.Ala177Val; *Capra sibirica*: p.Pro148Leu and p.Val162Leu; *Ovis aries*: p.Cys–8Tyr, p.Leu–2Ile, p.Ser15Ile, p.Asn27Asp, p.Asp43Glu, p.Lys48Arg, p.Gln56His, p.Val98Ala, p.Lys107Glu, p.Val130Ala, p.Ser152Pro, p.Val155Ile, p.Ser164Pro, p.Pro174Leu, p.Ala177Val, p.Met183Val, p.Ile185Thr, p.Leu196Ile, p.Arg200Gln, p.Gly201Glu, and p.Leu206Ile. * from [19]; ** from (present work); *** from [24].

Additional synonymous and nonsynonymous polymorphisms were detected in wild *Capra* species, including the substitution AJ011018.3:g.8900G>C (p.Val162Leu) shared by *C. ibex* (SJYO01041852.1) and *C. sibirica* (NIYN02007310.1); the mutation AJ011018.3:g.8759C>T (p.Pro115Ser) observed in *C. aegagrus* (JXYW01067823.1); a synonymous substitution AJ011018.3:g.8683G>A shared by *C. falconeri* and *C. nubiana* (CM141908.1), and the nonsynonymous mutation AJ011018.3:g.8859C>T (p.Pro148Leu) identified in *C. falconeri*, *C. nubiana* and *C. sibirica*. Notably, *C. pyrenaica* appears to be characterized by the fixation of the p.Leu148 (Supplementary Table S4, Figure 1).

The persistence of these polymorphisms across multiple caprine species indicates that they predate the radiation of the *genus Capra* and are more parsimoniously explained by retention of ancestral variations and incomplete lineage sorting than recurrent mutations. These patterns further indicate that, in addition to ancestral polymorphism, the *CSN2 locus* has undergone lineage-specific mutational diversification within the *genus Capra*.

Notably, the same coding variants affecting exon 2 (Leu/Ile at position −2 of the signal peptide) and exon 7 (Val/Ala at position 177) also occur in ovine *CSN2* sequences [24], indicating that these polymorphisms are shared across genera (Figure 1). This pattern is consistent with trans-species polymorphisms, suggesting that these allelic variants originated prior to the divergence of *Capra* and *Ovis* and have been maintained across species boundaries.

Although further analyses are required to formally test this hypothesis, the occurrence of shared exonic polymorphisms at the *CSN2 locus* suggests that milk-protein genes retain ancient allelic variation across ruminant lineages. Taken together, these observations support a hierarchical model of variation, in which polymorphisms are maintained within *Capra* as ancestral intra-generic variation and extend across *Capra* and *Ovis* as trans-species polymorphisms.

Alternative evolutionary processes, including introgression and convergent evolution, may also contribute to shared allelic states across taxa. However, the present *locus*-based dataset does not allow these mechanisms to be formally distinguished because robust discrimination would require genome-wide data or multiple independent *loci*. Instead, the observed combination of linked coding, non-coding, and structural variants is more consistent with persistence of ancestral polymorphism followed by incomplete lineage sorting than with recent introgression or repeated convergent evolution.

In this context, the *CSN2 locus* represents a multilayered evolutionary system in which coding polymorphisms, structural variation, and retroposon architecture jointly reflect deep evolutionary processes such as ancestral polymorphism retention, incomplete lineage sorting, and lineage-specific diversification.

### 3.6. Intron 4 Insertion/Deletion Polymorphism and Comparative Evidence Across Ruminant Species

Among the intronic variants identified, the 10 bp insertion/deletion in intron 4 (AJ011018.3:g.5292_5301) is particularly informative. Comparative genomic surveys indicate that the insertion state (+10 bp), with minor sequence variation, is widely distributed among representative mammalian lineages (Supplementary Table S5), a pattern consistent with its interpretation as the ancestral condition at this *locus* (Supplementary Figure S3).

Within *Bovidae*, however, the phylogenetic distribution of this InDel is complex and discontinuous. The deletion state (−10 bp) is primarily observed in derived caprine lineages, particularly within the *rupicolous* clade preceding the divergence of *Capra* and *Ovis*. In *C. hircus*, the polymorphism segregates among allelic lineages. Sequences carrying the *CSN2* A allele generally lack the insertion, although exceptions occur like the Xinong Saanen dairy goat (GenBank accessions JBBJUA010000004.1 and JAIWQT010000026.1). A similarly heterogeneous pattern is observed in *O. aries*, where both states are present in available genomic sequences (Supplementary Table S2), indicating ongoing intronic polymorphism within the species.

This patchy phylogenetic distribution is unlikely to be explained by recurrent mutation alone. Instead, it is consistent with the persistence of alternative insertion states across lineages. Within this framework, the observed distribution is consistent with the coexistence of both insertion and deletion states in ancestral bovid populations, followed by differential fixation in descendant lineages.

The occurrence of both insertion and deletion states in *Capra* and *Ovis* indicates that this polymorphism predates their divergence and, therefore, can be interpreted as a structural trans-species polymorphism, although it differs from classical cases in that the insertion represents the ancestral state and the deletion is a derived variant.

Although the functional significance of this intronic motif remains unknown, its distribution parallels that of other structural and coding variants at the *CSN2 locus*. Together with retroposon insertions and exonic polymorphisms, the intronic InDel contributes to a multilayered evolutionary signal in which structural variation reflects both deep ancestry and lineage-specific sorting processes.

### 3.7. Multilayered Evolution of the CSN2 Locus

#### 3.7.1. Retroposon Architecture and Coding Variation Mapped onto the *Bovidae* Phylogeny

Phylogenetic relationships within *Bovidae* were inferred using structural non-coding markers at the *CSN2*, including the presence/absence of four retroposon insertions (R0, R1, R2, R3), the buffalo specific LINE insertion L1_BT, the 10 bp intron 4 InDel (AJ011018.3:g.5292_5301) and the selected polymorphisms, namely p.Val177Ala, the two amino acids deletion (Tyr180/Pro181), and the substitution at position −2 of the signal peptide (p.Ile-2Leu). Genomic sequences of the *CSN2 locus* from all species represented in the phylogeny were examined to assess the distribution, structural configuration, and allelic state of these markers (Supplementary Table S2). Because retroposon insertions at orthologous *loci* are effectively irreversible, they provide a robust, near-homoplasy-free phylogenetic signal [28]. The intronic 10 bp InDel and coding variants (p.Val177Ala; Tyr180 and Pro181 deletion and p.Ile14Leu) were included to increase resolution among closely related taxa to capture evolutionary signals operating at different temporal and functional scales.

The distribution of retroposons at the *CSN2 locus* supports a predominantly hierarchical accumulation of rare genomic changes across *Bovidea* (Supplementary Figure S3). Basal *Artiodactyla* (*Suidae*, *Camelidae*, *Hippopotamidae*) consistently lack all retroposon insertions, whereas early-diverging *Ruminantia* (*Tragulidae*) lack R1, R2, and R3 elements and retain the +10 bp InDel, defining the plesiomorphic condition for the *locus*.

At a deeper level, comparative evidence across mammals indicates that Val177 represents the ancestral amino acid state in β-casein, whereas the derived Ala177 variant likely originated during early ruminant evolution and is subsequently fixed across most *Pecora*, providing a coding correlate to the transition from basal *Artiodactyls* to crown *Pecora*. Within *Pecora*, *Giraffidae* retain this ancestral configuration, whereas acquisition of R1 with a Bov-A2 sequence characterizes the common ancestor of crown *Pecora* excluding *Giraffidae*.

Based on published divergence-time estimates [29,30,31,32], the shared presence of R1 in *Cervidae* and *Moschidae* suggests that this insertion most likely predates the divergence between *Cervidae* and the *Moschidae–Bovidae* lineage (~27–25 Ma), indicating that the Bov-A2 insertion most parsimoniously originated in the stem lineage leading to the *Cervidae–Moschidae–Bovidae* clade rather than at the cervid–bovid node.

All examined *Bovidae* share R1, whereas R2 is absent in the archaic *Ovibovini* lineage.

Most *Bovidae* additionally possess the R2 insertion, which characterizes the predominant structural configuration of crown *Bovidae*. However, the archaic rupicolous caprine lineage comprising *Ovibos*, *Budorcas* and *Capricornis* exhibits a distinctive R1+ (Bov-A2), R2−, R3+ (Bov-B) and +10 bp configuration, indicating that the evolutionary history of the *CSN2 locus* is more complex than a strictly linear accumulation of retroposon insertions. The presence of the derived R3 insertion despite the apparent absence of R2 suggests either lineage-specific loss or secondary structural degeneration of an ancestrally inserted R2 element, retention of an ancestral R2− haplotypic background, or incomplete sorting of early retroposon architectures during basal caprine diversification [30,31,54]. Although ancient hybridization or introgression among early caprine lineages cannot be completely excluded as contributing factors, the present *locus*-based dataset does not allow these processes to be distinguished from incomplete lineage sorting or lineage-specific loss.

R2 became widely established during early *bovidae* diversification, although archaic *Ovibovini* retained an R2− configuration.

Within *Bovinae*, the absence of R3 together with fixation of the +10 bp InDel defines a conserved genomic configuration. In parallel, coding variation remains largely stable in this group, indicating that major structural changes rather than amino acid substitutions dominate this phase of evolution.

In addition to SINE-derived elements, a 291 bp LINE-1 insertion (L1_BT) was identified in the 5′ promoter region of the *CSN2* gene. This element is shared by *Syncerus* and all examined *Bubalus* species, including *Bubalus depressicornis*, but is absent from *Bison*, *Bos primigenius*, and all other investigated bovids, thereby representing a clear synapomorphy of the buffalo clade (*Syncerus* + *Bubalus*) and providing independent molecular evidence supporting Bubalina monophyly.

Beyond *Caprinae*, the incorporation of additional African bovids reveals a previously unrecognized structural signal at the *CSN2 locus*.

Several lineages of the African radiation, including *Reduncinae*, *Cephalophinae*, *Alcelaphinae*, and *Hippotraginae* (e.g., *Oryx gazella*, *Oryx dammah*, *Hippotragus equinus*), share a characteristic two-amino-acid deletion in β-casein. This deletion is also present in several *Antilopinae* lineages, including *Saiga tatarica*, *Litocranius walleri*, *Eudorcas thomsonii*, and *Nanger dama*, whereas other taxa such as *Aepyceros melampus* and *Neotragus moschatus* retain the full-length (+2 aa) configuration.

*Caprinae* are broadly characterized by retention of R3, although this insertion is also present in other bovid lineages, indicating a more ancient origin within *Bovidae*. The occurrence of R3 in both *Caprinae* and *Hippotraginae* suggests that the Bov-B element predates the divergence of these groups, followed by lineage-specific loss in other subfamilies.

Nevertheless, the presence of R3 in the absence of R2 within *Ovibos*, *Budorcas* and *Capricornis* demonstrates that the distribution of retroposon states within *Caprinae* does not conform to a strictly hierarchical R1 → R2 → R3 accumulation model (Supplementary Figure S3 and Table S2). Instead, early caprine evolution appears to have involved mosaic retention of alternative structural haplotypes, consistent with previous molecular studies reporting phylogenetic instability and rapid radiation among basal caprine lineages, including the deep evolutionary distinctiveness of extant and extinct *ovibovids* such as *Ovibos* and *Praeovibos* [30,31,55,56]. The predominant inferred ancestral caprine condition comprises R1+, R2+, R3+, and the +10 bp InDel. Alternatively, multiple structural haplotypes, including R2+ and R2− backgrounds, may already have been segregating during early caprine diversification, a scenario consistent with incomplete lineage sorting during rapid Miocene radiation events [31,54].

Early diverging rupicolous caprines retain these +10 configurations, indicating that the −10 bp state is derived within the caprine radiation. Additional coding variability emerges within *Caprinae*, including the Leu/Ile polymorphism at position −2 of the signal peptide. Comparative evidence confirms that leucine represents the ancestral mammalian state, whereas isoleucine is a derived variant restricted to caprine lineages.

A key structural transition occurs in the clade comprising *Oreamnos americanus* and *Ammotragus lervia*. Both species exhibit the combination R1+ (Bov-A), R2+, R3+, and the −10 bp InDel, defining a derived caprine configuration distinct from the Bov-A2/+10 ancestral state.

The retention of R2 in *Oreamnos* demonstrates that the R2− condition is restricted to the archaic *Ovibovini* lineage and is not a general feature of basal *rupicolous caprines*. This observation is consistent with previous phylogenetic studies placing *Oreamnos* outside the unstable *Ovibos*–*Budorcas*–*Capricornis* assemblage [30,55].

In these taxa, the R1 element corresponds to the Bov-A variant rather than Bov-A2, indicating that the Bov-A/Bov-A2 sequence divergence predates their separation and that the Bov-A variant was retained in this lineage. Given the effective irreversibility of retroposon insertions, the transition from Bov-A2 to Bov-A cannot be explained by simple excision or independent reinsertion. Instead, the trans-specific distribution of the two R1 sequence variants (Bov-A and Bov-A2), together with the associated ±10 bp InDel states, is consistent with their persistence from an ancestrally polymorphic *Capra–Ovis* stem lineage, followed by incomplete lineage sorting and lineage-specific retention. Most wild *Capra* species carry the R1+ (Bov-A) variant, whereas both +10 and −10 bp InDel states occur within the genus. A similar pattern appears to characterize *Ovis*, where both R1 sequence variants (Bov-A/Bov-A2) and the +10/−10 bp InDel remained polymorphic in the ancestral ovine lineage. Domestic *Ovis aries* exhibits near-equal frequency of R1 and InDel variants, further supporting the hypothesis of retained ancestral polymorphism at the *Capra–Ovis* stem. The persistence of both Ile and Leu variants likewise suggests that ancestral coding polymorphisms were maintained alongside structural variation during *caprine* diversification.

The observed distribution does not require multiple independent insertion events. A single origin of R1 (Bov-A2), an early origin of R2 followed by incomplete fixation or lineage-specific structural degeneration in *Ovibovini*, and an early origin of R3 followed by differential retention and lineage-specific loss are sufficient to explain the structural organization of accumulation of the retroposons at the *CSN2 locus*. Likewise, the lineage-specific insertion of L1_BT provides an independent and congruent signal within *Bovinae*. Such mosaic retention of retroposon architectures remains compatible with the generally near-homoplasy-free behavior associated with SINE insertions, while allowing rare lineage-specific structural remodeling events [28].

The contrasting distribution of R3, the p.Val177Ala substitution, and the −2 aa deletion highlights a pattern of overlapping but non-identical structural signals, consistent with lineage-specific sorting of ancestral genomic features. This interpretation is consistent with previous molecular phylogenetic analyses of *Caprinae* that recovered unstable relationships among *Ovibos*, *Budorcas*, *Capricornis* and related rupicolous taxa, supporting a scenario of rapid early caprine diversification [30,31,54,57].

Notably, additional localized coding variation is observed in the form of a lineage-specific insertion of the isoleucine residue at position 182 in *Syncerus caffer*. Because this insertion is absent from all other buffalo species, it likely represents an independent event nested within the broader buffalo clade defined by the L1_BT insertion, further indicating that both amino acid gains and losses have contributed to *CSN2* structural diversification.

The mosaic combination of R1 variants and InDel states across *Capra* and *Ovis* is therefore most parsimoniously explained by the persistence of ancestral haplotypes predating their divergence, rather than by recurrent mutation. Although historical introgression cannot be excluded, the combined distribution of linked coding, intronic, and retroposon variants is more consistent with ancestral polymorphism followed by incomplete lineage sorting.

The concordant behavior of structural (retroposons, InDel) and coding (p.Val177Ala, 2 aa deletion, p.Ile-2Leu, Ile182 insertion) markers demonstrates that the *CSN2 locus* does not simply reflect species phylogeny, but captures the interplay of ancestral polymorphism, structural innovation, and lineage-specific retention and loss across multiple evolutionary timescales. The unusual R2−/R3+ configuration retained in *Ovibovini* further indicates that the *locus* preserves traces of early caprine structural diversification that are no longer fully congruent with the species tree, a pattern previously inferred from mitochondrial and nuclear phylogenetic analyses of *Caprinae* [30,31].

#### 3.7.2. Comparison with Previously Published Phylogenies

The phylogenetic framework inferred from the retroposon architecture of the *CSN2 locus* (Supplementary Figure S3) is broadly congruent with previously published molecular and morphological reconstructions of *Ruminantia* and *Caprinae*. In particular, it corroborates the monophyly of *Pecora*, the basal divergence of *Tragulidae*, and the major splits within *Bovidae* and *Caprinae* consistently recovered in multilocus and genomic studies [29,32,55,58]. At deeper nodes, therefore, the topology recovered here aligns well with established phylogenetic hypotheses.

However, differences from earlier reconstructions emerge primarily at several shallow to intermediate nodes, where rapid diversification and incomplete lineage sorting are known to obscure bifurcating signal in sequence-based analyses [57,59,60,61].

Most mitochondrial and concatenated nuclear phylogenies place *Oreamnos americanus* either as the sister group to *Capra* as a basal caprine lineage, or as part of an unstable assemblage of rupicolous taxa, often with low or moderate support and marked topological variability [58,62,63]. Likewise, previous bioinformatic studies of β-casein evolution relied primarily on coding-sequence phylogenies and amino acid comparisons, without considering the broader structural organization of the *CSN2*, including retroposon architecture and intronic variation [64].

The present analysis is based on the presence, absence, and allelic state of rare retroposon insertions, rather than sequence divergence or branch length estimates, following the theoretical framework established for SINE- and LINE-based phylogenetics [28,63,64]. Retroposon insertions are nearly homoplasy-free; thus, they provide a robust source of phylogenetic information and have successfully resolved problematic relationships in cetaceans and other mammalian groups. Within *Caprinae*, this approach consistently places *Oreamnos* outside the crown *Capra–Ovis* clade while revealing retention of ancestral genomic features shared with early-diverging caprines, signals that are largely inaccessible to conventional sequence-based analysis methods.

A similar pattern emerges for *Capra falconeri*, whose phylogenetic position has varied across published trees, frequently clustering with extant *Capra* species but occasionally showing unstable affinities [30,63]. Such instability has often been attributed to rapid Miocene radiation and historical gene flow within *Caprinae* [58]. The partial sharing of derived retroposon states between *C. falconeri* and *Oreamnos americanus* identified here (R1 as Bov-A, R2+, R3+, −10 bp InDel; Supplementary Figure S3 and Table S2) instead suggests persistence of ancestral polymorphism at the caprine stem node. This interpretation is consistent with theoretical expectations for rare genomic changes and incomplete lineage sorting [60,65,66,67] and implies that the apparent conflict among sequence-based trees may reflect unresolved ancestral variation rather than contradictory phylogenetic signal.

A further distinction between the present framework and most previously published phylogenies concerns the explicit treatment of character evolution. Whereas sequence-based approaches typically model diversification as a series of successive bifurcations, the present retroposon-based reconstruction explicitly incorporates structural sequence remodelling within orthologous insertions (e.g., Bov-A2 to Bov-A transformation), transient allelic coexistence, and lineage-specific sorting [68]. Consequently, the divergence between *Capra* and *Ovis* is interpreted not simply as a bifurcation event, but as a sorting process involving alternative sequence states of a single R1 insertion and the associated 10 bp InDel, consistent with models of rapid radiation followed by differential fixation [59,60].

The inclusion of structural coding markers provides an additional level of information compared to previously published phylogenies. In particular, the two–amino-acid deletion in β-casein represents an independent phylogenetic event that complements the retroposon and intronic InDel markers used in the present reconstruction. Notably, its occurrence in several African bovid lineages, including *Reduncinae*, *Cephalophinae*, *Alcelaphinae*, and *Hippotraginae*, as well as in *Rupicapra rupicapra* suggests a single origin during early bovid diversification followed by lineage-specific retention across descendant clades.

The distribution of retroposon markers does not always coincide strictly with species-level phylogeny. As discussed in Section 3.7.1, the distribution of the R3 insertion is consistent with an origin of the Bov-B element predating the divergence of *Caprinae* and *Hippotraginae*, followed by lineage-specific loss in other bovid subfamilies [67,68].

In summary, the principal difference between the phylogeny proposed here and those previously published lies not in the overall topology of *Ruminantia*, which remains largely consistent across molecular, morphological, and genomic datasets, but in the interpretation of character evolution. The *CSN2 locus* reveals a multilayered evolutionary signal in which retroposon insertions, intronic structural variation, and coding polymorphisms capture different temporal and evolutionary processes. By integrating presence/absence data, allelic states, and structural variation, the present framework refines existing phylogenetic hypotheses and highlights the central role of incomplete lineage sorting, ancestral polymorphism, and lineage-specific retention and loss in shaping the evolutionary history of Bovidae [58,65,66,68].

### 3.8. Integrating Retroposon Architecture and Coding Variation in Caprinae Allele CSN2 Phylogeny

*Locus*-specific analyses of the *CSN2* gene in *Caprinae* reveal a complex evolutionary history characterized by the coexistence of deeply divergent allelic lineages, ancient interallelic recombination, and incomplete sorting of ancestral polymorphisms.

The segregation of *CSN2* alleles into two major evolutionary lineages was consistently recovered by multiple independent analytical approaches, including NeighborNet reconstruction, retroposon architecture, SNP- and InDel-based haplotype comparisons, and recombination analyses. These complementary approaches, based on distinct sources of evolutionary information, consistently identified the same pattern of allelic subdivision, hereafter referred to as the A-type and C-type lineages. The concordance among these independent analyses indicates that they represent ancient, deeply diverged allelic classes rather than recent mutational derivatives.

Recombination analyses were conducted using GARD on a multiple sequence alignment spanning the full *CSN2* gene sequence (10,796 bp) and including representative alleles from domestic and wild caprine taxa. The dataset comprised *CSN2* A2 alleles from *C. hircus* and *C. falconeri*, *CSN2* A and C alleles from *C. aegagrus*, the *CSN2* A1, C1, and 01 alleles from domestic goat, and the allele *CSN2* CX from *C. sibirica*. GARD analyses provided strong statistical evidence for recombination within the *locus*. A single-breakpoint model identified a major breakpoint at alignment position 4521, markedly improving model fit compared to the no-recombination model (ΔAICc > 8000). Allowing a second breakpoint further improved model fit relative to the single-breakpoint analysis, identifying breakpoints at positions 4521 and 9617. Although the second breakpoint further improved the statistical fit of the model, the principal phylogenetic incongruence and biological interpretation were associated with the first breakpoint at position 4521. Notably, the recombination signal primarily involves the 5′ portion of the gene, overlapping the region that includes exon 2, which encodes the signal peptide region of β-casein. Phylogenetic incongruence involving this region contrasts with the stable lineage assignment observed across downstream regions, indicating that the inferred interallelic recombination event primarily reshuffled ancestral A-type and C-type variants at exon 2.

**Figure 2:**
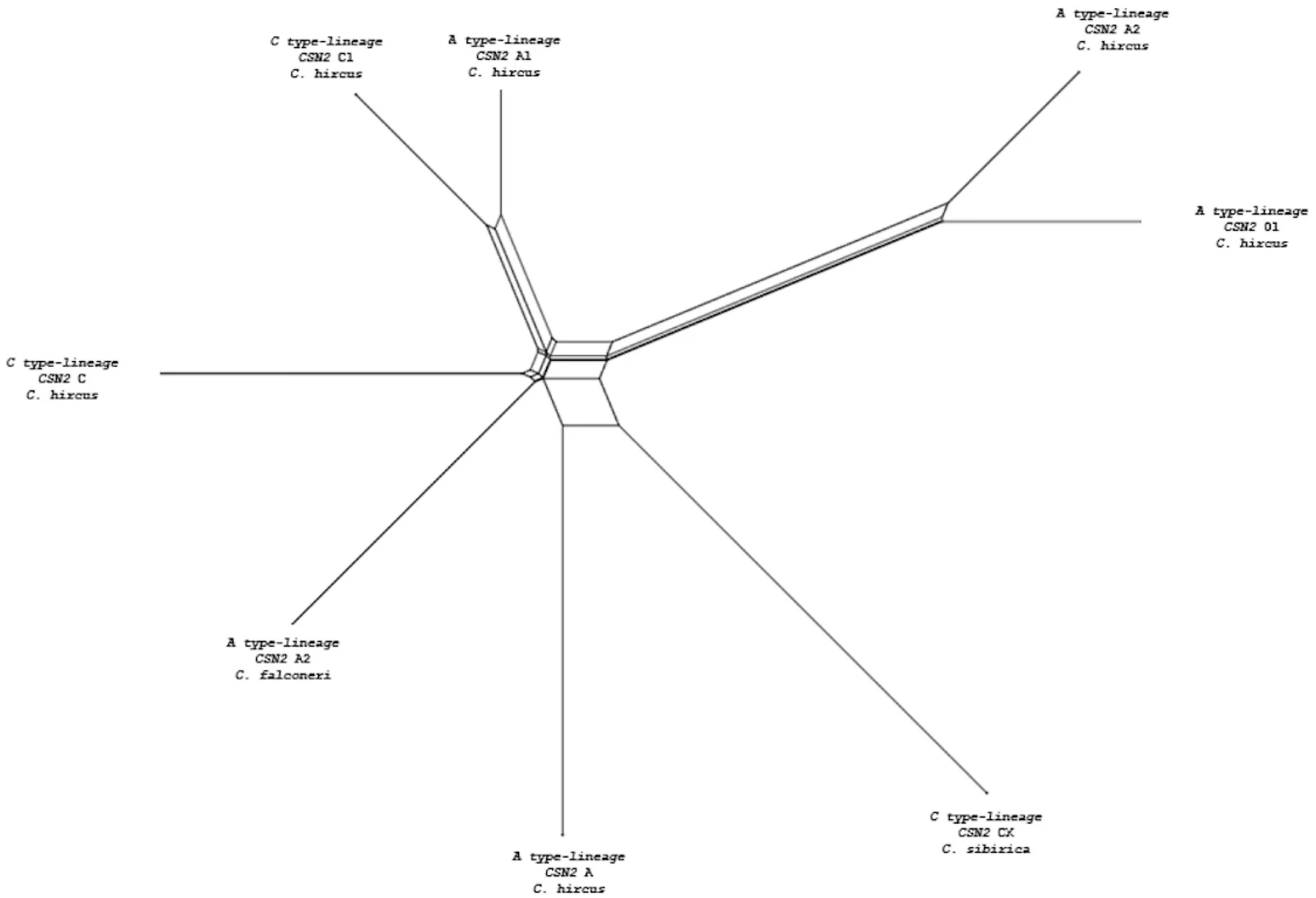
NeighborNet network generated in SplitsTree from full-length *CSN2* genomic haplotypes representing major caprine allelic lineages. Terminal labels indicate representative *CSN2* alleles and associated intronic insertion/deletion states. The network includes A-type and C-type haplotypes together with recombinant candidate alleles identified through GARD and Phi-test analyses. Branch reticulation reflects conflicting phylogenetic signals among genomic regions of the *CSN2 locus*. A schematic evolutionary interpretation of the network is provided in Figure 3.

**Figure 3:**
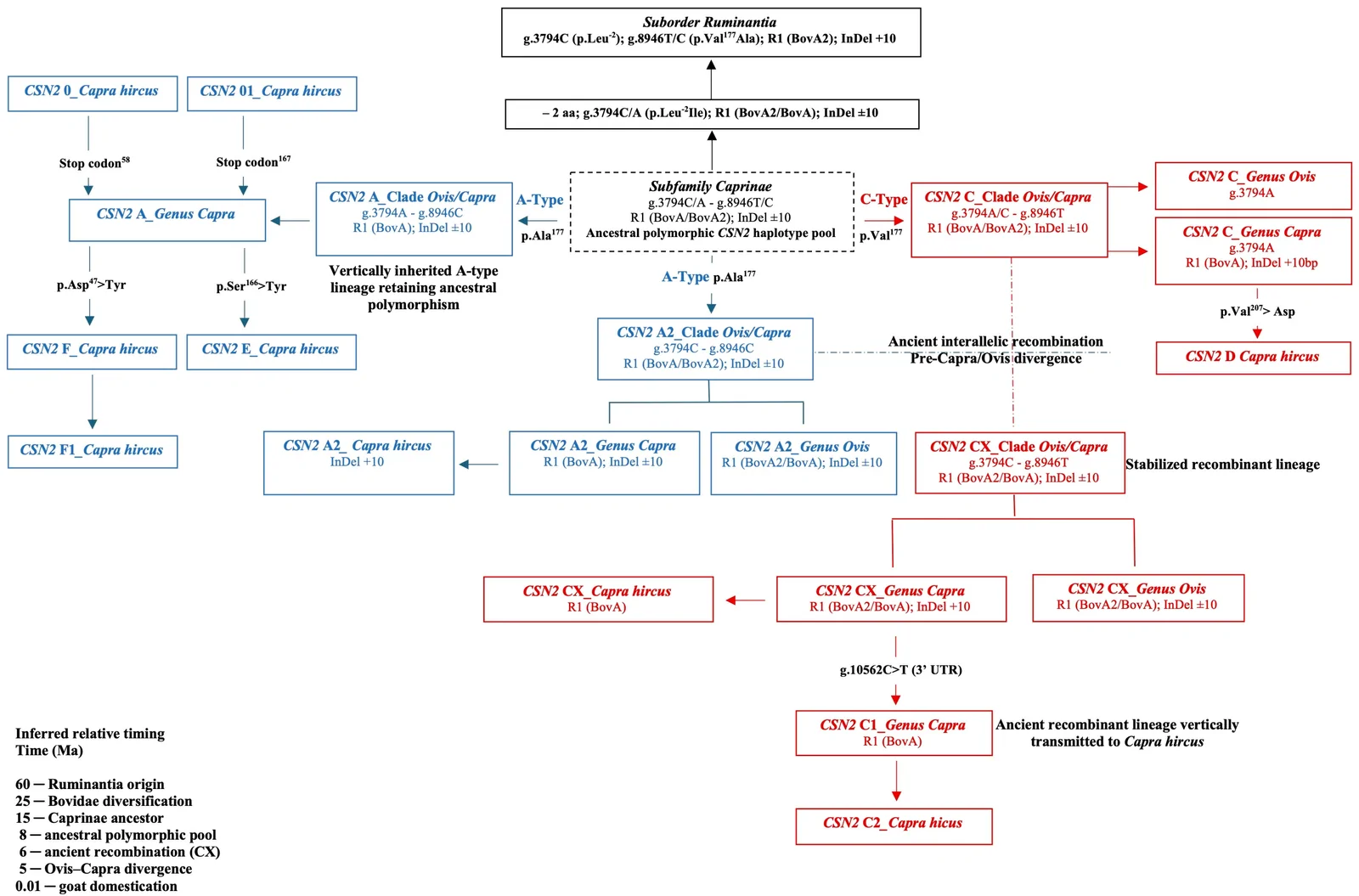
NeighborNet network reconstructed from full-length *CSN2* genomic sequences of domestic and wild *Caprinae* taxa using SplitsTree. The analysis includes representative A-type and C-type allelic lineages, recombinant candidate alleles, and wild-species haplotypes. The network was generated from aligned genomic haplotypes spanning the complete *CSN2 locus*. Reticulate relationships among alleles are visualized together with associated retroposon configurations, coding polymorphisms, and intronic insertion/deletion variants considered in the comparative analyses.

Phylogenetic trees inferred from individual GARD-defined partitions revealed topological incongruence involving alleles such as *CSN2* C1 and the rarest A1 variant, whereas representative A-type (*CSN2* A2) and C-type (*CSN2* C) alleles maintained stable phylogenetic affinities across all partitions. This pattern is indicative of ancient interallelic recombination between the A-type and C-type lineages. Consistently, NeighborNet reconstruction in SplitTree recovered a reticulated topology dominated by two major clusters corresponding to the A-type and C-type lineages, connected by limited reticulation involving the *CSN2* CX allele. The concordance among GARD breakpoint analyses, the permutation-based Phi test (*p* < 0.001), and the NeighborNet network provides consistent evidence for recombination and, together with the trans-generic distribution of recombinant haplotypes, supports the occurrence of at least one major ancient interallelic recombination event, followed by independent evolutionary trajectories within each lineage (Figure 2 and Figure 3). The inferred recombination was interpreted as ancient because the recombinant allelic configuration is shared across Capra and Ovis and is consistently recovered by independent analytical approaches. This distribution is more parsimoniously explained by a recombination event predating the divergence of the two genera than by recurrent independent mutations generating identical allelic combinations.

Ancestral state reconstruction across mammals supports g.3794C, g.8946T, and g.10562C as ancestral states at key polymorphic positions, indicating that the divergence between A-type and C-type lineages was preceded by ancestral polymorphism at multiple sites. The presence of the 10 bp insertion/deletion polymorphism in both caprine and ovine lineages further supports an origin predating the divergence of *Capra* and *Ovis*. In contrast, polymorphism at position g.10562 appears restricted to *Caprinae*, indicating subsequent lineage-specific diversification following the initial separation of the two major allelic lineages.

Consistent with this interpretation, NeighborNet analyses place *CSN2* C1 as a stable terminal lineage associated with the ancient recombination signal between the A-type and C-type allelic backgrounds. The position of C1 within the reticulated network topology, together with the absence of additional lineage-specific recombination breakpoints, indicates that this allele appears to represent a stabilized recombinant lineage inherited from an ancient interallelic recombination event. In contrast, the *CSN2* A1 allele appears to be extremely rare within the currently available genomic data for *Capra hircus*. Among more than fifty publicly available whole-genome assemblies, only a single sequence corresponds to the A1 allele, whereas more than twelve genomes carry the C1 allele (Supplementary Table S2). Although this observation is consistent with a genuinely low frequency of the A1 allele, a contribution of sampling bias in the currently available genomic datasets cannot be excluded. Consequently, the A1 allele was excluded from downstream phylogenetic modelling due to its extremely low representation. Thus, the following analyses focus on the C1 lineage, which represents the only recombinant allele consistently recovered across multiple genomic datasets. Intronic SNP patterns further support the antiquity of the recombination signal underlying the C1 lineage. Reduced datasets analyses showed that exclusion of representative parental A-type and C-type alleles markedly weakened the recombination signal detected by GARD, arguing against recent or recurrent recombination among derived alleles and reinforcing the interpretation of an ancient interallelic recombination event within an ancestral caprine population.

Additional evidence derives from intergeneric comparisons. In the *genus Ovis*, three of the same allelic combinations observed in *Capra* are reported in public databases, including g.3794C/g.8946C, g.3794A/g.8946T, and g.3794C/g.8946T haplotypes [24]. Considering *Capra* and *Ovis* jointly, all four theoretically possible combinations between nucleotide states at g.3794 (C/A) and g.8946 (C/T) are observed, corresponding to the amino acid haplotypes Leu-2/Ala177, Leu-2/Val177, Ile-2/Val177, and Ile-2/Ala177.

Their trans-generic distribution indicates that interallelic recombination between the A-type and C-type lineages most likely occurred before complete lineage sorting of the *Capra–Ovis* ancestral polymorphism, resulting in allelic combinations that were subsequently retained as ancestral polymorphisms and differentially sorted in the two genera.

The *locus*-specific phylogeny of *CSN2* is consistent with the retroposon architecture characterizing *Caprinae*. The R1 insertion represents a single orthologous event that originated in the stem lineage of crown *Pecora* and most likely arose in the Bov-A2 configuration, which is widely retained across pecoran lineages and therefore represents the ancestral state. Within advanced *Caprinae*, the Bov-A configuration appears to have emerged secondarily as a derived sequence variant of the same insertion, most plausibly through localized sequence remodeling affecting the internal SINE body. Notably, the SNP-defined A-type and C-type allelic lineages do not correspond strictly to retroposon configurations. In *Capra*, all analyzed alleles share the Bov-A configuration regardless of lineage assignment, whereas in *Ovis aries* the two R1 variants show a structured association with the intronic InDel polymorphism. This partial decoupling between SNP-defined haplotypes and retroposon sequence states likely reflects the combined effects of ancient interallelic recombination and incomplete lineage sorting acting on an ancestral polymorphic pool. The observed decoupling may also have been influenced by historical recombination between the retroposon-containing and coding regions of the *CSN2 locus*, consistent with the recombination signal detected by GARD.

Collectively, these results support a model in which the evolutionary history of the *CSN2 locus* in *Caprinae* was shaped by (i) early divergence of two deeply separated allelic lineages, (ii) at least one ancient interallelic recombination event generating mosaic alleles, and (iii) incomplete lineage sorting coupled with lineage-specific mutational diversification. This multilayered evolutionary scenario parsimoniously explains the reticulated phylogenetic signal observed at the *locus* and integrates allelic diversity, recombination dynamics, and retroposon-based phylogenetic evidence in *Caprinae*.

## 4. Conclusions

The *CSN2 locus* reveals a complex evolutionary history shaped by the combined effects of coding and non-coding variation, structural polymorphisms, retroposon insertions, ancestral polymorphisms, incomplete lineage sorting, and ancient recombinations. Together, these processes generated a multilayered evolutionary signal that cannot be adequately explained by a strictly bifurcating model.

Comparative analyses indicate that several coding and non-coding variants predate the divergence of major caprine lineages and have been maintained across speciation events, supporting the persistence of ancestral polymorphisms and the occurrence of trans-species variation within *Capra* and *Ovis*. Recombination analyses further support at least one ancient interallelic recombination event between the major *CSN2* lineages, contributing to the reticulate structure of the *locus*.

A major finding of this study is the characterization of the retroposon architecture of *CSN2*. The distribution of SINE and LINE insertions provides a robust phylogenetic framework that complements sequence-based analyses and helps distinguish true evolutionary relationships from patterns generated by incomplete lineage sorting and differential retention of ancestral states.

These results identify *CSN2* as a valuable model for investigating genome evolution and phylogenetic diversification in *Bovidae* and highlight the utility of retroposons for resolving complex evolutionary histories that are not fully captured by conventional sequence-based approaches. Beyond its evolutionary significance, the framework presented here provides a genomic reference for future studies aimed at linking structural and allelic diversity at the *CSN2 locus* with functional variation in milk proteins, breed characterization, conservation genetics, and genomic selection in dairy ruminants. Extending this integrative evolutionary approach to the remaining casein genes, particularly *CSN1S1* and *CSN1S2*, would be especially valuable to determine whether the interallelic recombination events and retroposon architectures previously reported at these *loci* likewise reflect multilayered evolutionary dynamics involving ancestral polymorphism, incomplete lineage sorting, and ancient recombination. Confirmation of similar patterns would suggest that the evolutionary processes identified here represent general features of the casein gene cluster rather than characteristics unique to *CSN2*, thereby providing a broader framework for understanding the evolution and functional diversification of milk protein genes in ruminants.

## Data Availability

The data presented in this study are available on request from the corresponding author.

## Supplementary Materials

The following supporting information can be downloaded at: https://www.mdpi.com/article/10.3390/ani16162482/s1, Figure S1: Comparative alignment of nucleotide sequences from the partial exon 7 region of the CSN2 gene and corresponding deduced amino acid sequences across representative mammalian taxa. Gaps indicate the absence of codon segments, whereas grey shading indicates the amino acid residue at position 177 of the mature ruminants β-casein protein. Taxa are grouped according to phylogenetic affinity to facilitate comparison of coding structural variation, including lineage-specific insertion/deletion events, across Bovidae and selected mammalian outgroups; Figure S2: Alignment of nucleotide sequences from the *CSN2* signal peptide coding region across representative mammalian taxa. Dashes indicate identity with the caprine reference sequence. The SNP AJ011018.3:g.3794A>C (highlighted in grey) results in an Ile→Leu substitution at position −2 of the signal peptide. Comparative analysis across mammals supports leucine as the predominant ancestral state, whereas isoleucine represents a derived variant segregating within *Capra* and *Ovis*. Additional substitutions reflect lineage-specific variation within an otherwise conserved signal peptide region; Figure S3: Evolutionary reconstruction of CSN2-associated molecular states across Ruminantia and related Cetartiodactyla; Table S1: Taxonomic composition of the dataset and CSN2 genomic records analysed. Part A: summary of the taxonomic representation of the dataset across the mammalian groups included in this study, reporting the number of species and CSN2 genomic records analysed for each major taxonomic group. Part B: complete list of CSN2 genomic records retrieved from public databases (GenBank, RefSeq, and WGS assemblies), including accession numbers, genomic coordinates, sequence orientation, and breed (when available) or species information used in the comparative analyses; Table S2: Genomic sequences analyzed for comparative investigation of the *CSN2 locus*. The table reports accession numbers, genomic coordinates, breed or species information, SNP-defined haplotypes, and associated retroposon/intronic insertion-deletion configurations. Representative allelic lineages are grouped according to A-type and C-type haplotypic backgrounds; Table S3: Coding and non-coding polymorphisms identified at the *CSN2 locus* across representative caprine alleles and related taxa. Nucleotide states are reported relative to the Capra hircus reference sequence AJ011018.3; Table S4: Detailed genotypic characterization of CSN2 polymorphisms identified from public SRA (Sequence Read Archive) datasets across wild (A) and domestic goats (Capra hircus) (B) taxa. The table includes known polymorphisms, novel SNPs, intronic insertion/deletion states, inferred haplotypes, breed information, and sequence accession identifiers; Table S5: Comparative matrix of nucleotide variation within the conserved 10 bp insertion located in intron 4 of the *CSN2 locus* across representative mammalian taxa. Columns represent nucleotide positions within the insertion, whereas rows correspond to species grouped according to phylogenetic affinity. The consensus sequence (AGTACAAACT) is shown at the top. Grey shading indicates nucleotide states differing from the consensus sequence. Only taxa retaining the orthologous insertion state were included in the comparison.

## References

[1] Rijnkels M. (2002). Multispecies comparison of the casein gene *loci* and evolution of casein gene family. J. Mammary Gland Biol. Neoplasia.

[2] Kawasaki K., Lafont A.G., Sire J.Y. (2011). The evolution of milk casein genes from tooth genes before the origin of mammals. Mol. Biol. Evol..

[3] Parveen S., Zhu P., Shafique L., Lan H., Xu D., Ashraf S., Ashraf S., Sherazi M., Liu Q. (2023). Molecular characterization and phylogenetic analysis of casein gene family in *Camelus ferus*. Genes.

[4] Rehman S.U., Feng T., Wu S., Luo X., Lei A., Luobu B., Hassan F.U., Liu Q. (2021). Comparative genomics, evolutionary and gene regulatory regions analysis of casein gene family in *Bubalus bubalis*. Front. Genet..

[5] Yu-Lee L.Y., Richter-Mann L., Couch C.H., Stewart A.F., Mackinlay A.G., Rosen J.M. (1986). Evolution of the casein multigene family: Conserved sequences in the 5’ flanking and exon regions. Nucleic Acids Res..

[6] Groenen M.A.M., Dijkhof R.J.M., Verstege A.J.M., van der Poel J.J. (1993). The complete sequence of the gene encoding bovine αs2-casein. Gene.

[7] Farrell H.M., Jimenez-Flores R., Bleck G.T., Brown E.M., Butler J.E., Creamer L.K., Hicks C.L., Hollar C.M., Ng-Kwai-Hang K.F., Swaisgood H.E. (2004). Nomenclature of the proteins of cows’ milk-Sixth revision. J. Dairy Sci..

[8] Gallinat J.L., Qanbari S., Drögemüller C., Pimentel E.C.G., Thaller G., Tetens J. (2013). DNA-based identification of novel bovine casein gene variants. J. Dairy Sci..

[9] Cieślińska A., Fiedorowicz E., Rozmus D., Sienkiewicz-Szłapka E., Jarmołowska B., Kamiński S. (2022). Does a little difference make a big difference? Bovine β-Casein A1 and A2 variants and human health-An update. Int. J. Mol. Sci..

[10] Roberts B., Di Tullio P., Vitale J., Hehir K., Gordon K. (1992). Cloning of the goat β-casein-encoding gene and expression in transgenic mice. Gene.

[11] Mahé M.F., Grosclaude F. (1993). Polymorphism of β-casein in the Creole goat of Guadeloupe: Evidence for a null allele. Genet. Sel. Evol..

[12] Persuy M.A., Printz C., Medrano J.F., Mercier J.C. (1999). A single nucleotide deletion resulting in a premature stop codon is associated with marked reduction of transcripts from a goat beta-casein null allele. Anim. Genet..

[13] Neveu C., Mollé D., Moreno J., Martin P., Léonil J. (2002). Heterogeneity of caprine beta-casein elucidated by rp-hplc/ms: Genetic variants and phosphorylations. J. Protein Chem..

[14] Galliano F., Saletti R., Cunsolo V., Foti S., Marletta D., Bordonaro S., D’Urso G. (2004). Identification and characterization of a new *β*-casein variant in goat milk by high-performance liquid chromatography with electrospray ionization mass spectrometry and matrix-assisted laser desorption/ionization mass spectrometry. Rapid Commun. Mass Spectrom..

[15] Caroli A., Chiatti F., Chessa S., Rignanese D., Bolla P., Pagnacco G. (2006). Focusing on the goat casein complex. J. Dairy Sci..

[16] Nicolai M.A., Garro G., Caira S., Mauriello R., Quarto M., De Pascale S., Chianese L. (2021). Occurrence of quantitative genetic polymorphism at the caprine β-CN *locus*, as determined by a proteomic approach. Int. Dairy J..

[17] Cosenza G., Pauciullo A., Colimoro L., Mancusi A., Rando A., Di Berardino D., Ramunno L. (2007). An SNP in the goat *CSN2* promoter region is associated with the absence of β-casein in milk. Anim. Genet..

[18] Cosenza G., Albarella S., D’Anza E., Iannuzzi A., Selvaggi M., Pugliano M., Galli T., Saralli G., Ciotola F., Peretti V. (2023). A new AS-PCR method to detect *CSN201* allele, genotyping at Ca-sensitive caseins *loci* and milk traits association studies in autochthonous Lazio goats. Animals.

[19] Cosenza G., Pauciullo A., Gallo D., Di Berardino D., Ramunno L. (2005). A SspI PCR-RFLP detecting a silent allele at the goat *CSN2 locus*. J. Dairy Res..

[20] Chessa S., Rignanese D., Küpper J., Pagnacco G., Erhardt G., Caroli A. (2008). Short communication: The beta-casein (*CSN2*) silent allele C1 is highly spread in goat breeds. J. Dairy Sci..

[21] Moatsou G., Mollé D., Moschopoulou E., Valérie G. (2007). Study of caprine β-casein using reversed-phase high-performance liquid chromatography and mass spectroscopy: Identification of a new genetic variant. Protein J..

[22] Vinesh P.V., Brahma B., Kaur R., Datta T.K., Goswami S.L., De S. (2013). Characterization of β-casein gene in Indian riverine buffalo. Gene.

[23] Fan X.Y., Xue J., Qiu L.H., Wang R.P., Miao Y.W. (2021). Polymorphisms of beta casein (*CSN2*) CDS and inference of its variants in river and swamp water buffalo (*Bubalus bubalis*). J. Anim. Plant Sci..

[24] Ali M., Gautam D., Deepika S., Meena A.S., Chera J., De S. (2022). The genetic variations in *CSN2* gene of Indian sheep breeds affect its protein stability and function. Small Rumin. Res..

[25] Li X., Spencer G.W.K., Ong L., Gras S.L. (2022). Beta casein proteins—A comparison between caprine and bovine milk. Trends Food Sci. Technol..

[26] Kriegs J.O., Churakov G., Kiefmann M., Jordan U., Brosius J., Schmitz J. (2006). Retroposed elements as archives for the evolutionary history of placental mammals. PLoS Biol..

[27] Nikaido M., Nishihara H., Okada N. (2022). SINEs as credible signs to prove common ancestry in the tree of life: A brief review of pioneering case studies in retroposon systematics. Genes.

[28] Nikaido M., Matsuno F., Hamilton H., Brownell R.L., Cao Y., Ding W., Zuoyan Z., Shedlock A.M., Fordyce R.E., Hasegawa M. (2001). Retroposon analysis of major cetacean lineages: The monophyly of toothed whales and the paraphyly of river dolphins. Proc. Natl. Acad. Sci. USA.

[29] Hassanin A., Douzery E.J.P. (2003). Molecular and morphological phylogenies of Ruminantia and the alternative position of the Moschidae. Syst. Biol..

[30] Ropiquet A., Hassanin A. (2005). Molecular phylogeny of caprines (Bovidae, Antilopinae): The question of their origin and diversification during the miocene. J. Zool. Syst. Evol. Res..

[31] Bibi F. (2013). A multi-calibrated mitochondrial phylogeny of extant bovidae (Artiodactyla, Ruminantia) and the importance of the fossil record to systematics. BMC Evol. Biol..

[32] Chen L., Qiu Q., Jiang Y., Wang K., Lin Z., Li Z., Bibi F., Yang Y., Wang J., Nie W. (2019). Large-scale ruminant genome sequencing provides insights into their evolution and distinct traits. Science.

[33] Bryant D., Moulton V. (2004). Neighbor-net: An agglomerative method for the construction of phylogenetic networks. Mol. Biol. Evol..

[34] Huson D.H., Bryant D. (2024). The SplitsTree app: Interactive analysis and visualization using phylogenetic trees and networks. Nat. Methods.

[35] Kosakovsky Pond S.L., Posada D., Gravenor M.B., Woelk C.H., Frost S.D.W. (2006). Automated phylogenetic detection of recombination using a genetic algorithm. Mol. Biol. Evol..

[36] Weaver S., Shank S.D., Spielman S.J., Li M., Muse S.V., Kosakovsky Pond S.L. (2018). Datamonkey 2.0: A Modern Web Application for Characterizing Selective and Other Evolutionary Processes. Mol. Biol. Evol..

[37] Kosakovsky Pond S.L., Poon A.F.Y., Velazquez R., Weaver S., Hepler N.L., Murrell B., Shank S.D., Magalis B.R., Bouvier D., Nekrutenko A. (2020). HyPhy 2.5-A Customizable Platform for Evolutionary Hypothesis Testing Using Phylogenies. Mol. Biol. Evol..

[38] Bruen T.C., Philippe H., Bryant D. (2006). A simple and robust statistical test for detecting the presence of recombination. Genetics.

[39] Cosenza G., Iannaccone M., Pico B.A., Ramunno L., Capparelli R. (2016). The SNP g.1311T>C associated with the absence of β-casein in goat milk influences *CSN2* promoter activity. Anim. Genet..

[40] Guan D., Mármol-Sánchez E., Cardoso T.F., Such X., Landi V., Tawari N.R., Amills M. (2019). Genomic analysis of the origins of extant casein variation in goats. J. Dairy Sci..

[41] Dou M., Li M., Zheng Z., Chen Q., Wu Y., Wang J., Shan H., Wang F., Dai X., Li Y. (2023). A missense mutation in *RRM1* contributes to animal tameness. Sci. Adv..

[42] Adelson D.L., Raison J.M., Edgar R.C. (2009). Characterization and distribution of retrotransposons and simple sequence repeats in the bovine genome. Proc. Natl. Acad. Sci. USA.

[43] Bonsing J., Ring J.M., Stewart A.F., Mackinlay A.G. (1988). Complete nucleotide sequence of the bovine beta-casein gene. Aust. J. Biol. Sci..

[44] Koczan D., Hobom G., Seyfert H.M. (1991). Genomic organization of the bovine alpha-S1 casein gene. Nucleic Acids Res..

[45] Provot C., Persuy M.A., Mercier J.C. (1995). Complete sequence of the ovine β-casein-encoding gene and interspecies comparison. Gene.

[46] Ramunno L., Cosenza G., Rando A., Illario R., Gallo D., Di Berardino D., Masina P. (2004). The goat αs1-casein gene: Gene structure and promoter analysis. Gene.

[47] Cosenza G., Feligini M., Mancusi A., D’Avino A., Coletta A., Di Berardino D., Ramunno L. (2009). Italian Mediterranean river buffalo *CSN2* gene structure and promoter analysis. Ital. J. Anim. Sci..

[48] Cosenza G., Gallo D., Auzino B., Gaspa G., Pauciullo A. (2021). Complete *CSN1S2* characterization, novel allele identification and association with milk fatty acid composition in river buffalo. Front. Genet..

[49] Pauciullo A., Giambra I.J., Iannuzzi L., Erhardt G. (2014). The β-casein in camels: Molecular characterization of the *CSN2* gene, promoter analysis and genetic variability. Gene.

[50] Pauciullo A., Shuiep E.T., Ogah M.D., Cosenza G., Di Stasio L., Erhardt G. (2019). Casein gene cluster in camelids: Comparative genome analysis and new findings on haplotype variability and physical mapping. Front. Genet..

[51] Pauciullo A., Versace C., Gaspa G., Letaief N., Bedhiaf-Romdhani S., Fulgione A., Cosenza G. (2023). Sequencing and characterization of αs2-casein gene (*CSN1S2*) in the Old-World camels have proven genetic variations useful for the understanding of species diversification. Animals.

[52] Richardson B.C., Creamer L.K. (1974). Comparative micelle structure. III. The isolation and chemical characterization of caprine β1-casein and β2-casein. Biochim. Biophys. Acta Protein Struct..

[53] Gatesy J., Hayashi C., Cronin M.A., Arctander P. (1996). Evidence from milk casein genes that cetaceans are close relatives of hippopotamid artiodactyls. Mol. Biol. Evol..

[54] Hassanin A., Douzery E.J.P. (1999). The tribal radiation of the family Bovidae (Artiodactyla) and the evolution of the mitochondrial cytochrome *b* gene. Mol. Phylogenet. Evol..

[55] Hernández Fernández M., Vrba E.S. (2005). A complete estimate of the phylogenetic relationships in Ruminantia: A dated species-level supertree of the extant ruminants. Biol. Rev..

[56] Campos P.F., Sher A., Mead J.I., Tikhonov A., Buckley M., Collins M., Willerslev E., Gilbert M.T.P. (2010). Clarification of the taxonomic relationship of the extant and extinct ovibovids, *Ovibos*, *Praeovibos*, *Euceratherium* and *Bootherium*. Quat. Sci. Rev..

[57] Matthee C.A., Davis S.K. (2001). Molecular insights into the evolution of the family Bovidae: A nuclear DNA perspective. Mol. Biol. Evol..

[58] Hassanin A., Delsuc F., Ropiquet A., Hammer C., Jansen Van Vuuren B., Matthee C., Ruiz-Garcia M., Catzeflis F., Areskoug V., Nguyen T.T. (2012). Pattern and timing of diversification of Cetartiodactyla (Mammalia, Laurasiatheria), as revealed by a comprehensive analysis of mitochondrial genomes. C R Biol..

[59] Pamilo P., Nei M. (1988). Relationships between gene trees and species trees. Mol. Biol. Evol..

[60] Degnan J.H., Rosenberg N.A. (2009). Gene tree discordance, phylogenetic inference and the multispecies coalescent. Trends Ecol. Evol..

[61] Mereu P., Pirastru M., Scarpa F., Zedda M., Bogliolo L., Naitana S., Leoni G.G. (2025). Mouflon and domestic sheep phylogeny: Ancestry, domestication, and evolutionary dynamics. Life.

[62] Ropiquet A., Hassanin A. (2006). Hybrid origin of the Pliocene ancestor of wild goats. Mol. Phylogenet. Evol..

[63] Bibi F., Bukhsianidze M., Gentry A., Geraads D., Kostopoulos D., Vrba E. (2009). The fossil record and evolution of Bovidae: State of the field. Palaeontol. Electron..

[64] Vincent S., Momoh O., Yakubu A. (2014). Bioinformatics analysis of beta-casein gene in some selected mammalian species. Res. Opin. Anim. Vet. Sci..

[65] Shedlock A.M., Okada N. (2000). SINE insertions: Powerful tools for molecular systematics. BioEssays.

[66] Ray D.A., Xing J., Salem A.H., Batzer M.A. (2006). SINEs of a nearly perfect character. Syst. Biol..

[67] Nilsson M.A., Klassert D., Bertelsen M.F., Hallström B.M., Janke A. (2012). Activity of ancient RTE retroposons during the evolution of cows, spiral-horned antelopes, and nilgais (Bovinae). Mol. Biol. Evol..

[68] Nijman I.J., Van Tessel P., Lenstra J.A. (2002). SINE retrotransposition during the evolution of the Pecoran ruminants. J. Mol. Evol..

