# Supplementary material for: The Bovidae CSN2 Locus as a Multilayered Evolutionary System: Evidence from Retroposons, Trans-Species Variation and Ancient Recombination
Source: Animals (Basel). 2026 Aug 10;16(16):2482. doi: 10.3390/ani16162482 (PMC13508899; doi:10.3390/ani16162482)
Supplement: Supplementary file 1 [file animals-16-02482-s001.zip › Supplementary Figure S1.pdf]

```
                                                   177
 A  L  P  P  Q  P  L  W  S  V             P  Q  P  K  V  L  P  I  P  Q  Q  V  V  P  Y  P    Q  R  A  V  P  V  Q  A  L  L  L  N  Q  E  L
GCACTTCCCCCTCAGCCCCTGTGGTCTGTT------------CCTCAGCCCAAAGTCCTGCCTATCCCCCAGCAAGTGGTGCCCTACCCT---CAGAGAGCTGTGCCTGTTCAAGCCCTTCTGCTCAACCAAGAACTT  1

 M  L  T  P  Q  S  V  L  S  I             P  Q  P  K  V  L  P  F  P  Q  Q  V  V  P  Y  L    Q  R  D  M  P  L  Q  A  F  L  P  Y  Q  E  S
ATGCTTACTCCTCAGTCAGTGCTGTCTATC------------CCACAGCCCAAAGTCTTGCCTTTTCCTCAGCAAGTGGTGCCCTACCTC---CAGAGAGATATGCCCCTACAAGCCTTTCTGCCATACCAGGAATCT  2

 M  L  P  P  Q  P  L  L  S  I             P  Q  P  K  V  M  P  F  P  Q  Q  I  V  P  Y  L    Q  R  D  M  P  V  Q  T  L  L  L  Y  Q  D  A
ATGCTTCCTCCTCAGCCACTGCTGTCCATC------------CCACAGCCCAAAGTCATGCCTTTTCCCCAGCAAATTGTGCCCTACCTC---CAGAGAGACATGCCCGTGCAAACCCTACTGCTGTACCAGGATGCC  3

 M  L  T  P  Q  P  L  L  S  I             P  Q  S  K  V  L  A  F  P  Q  Q  V  M  P  F  P    Q  R  D  M  P  P  Q  A  F  L  L  Y  Q  E  P
ATGCTTACTCCTCAGCCATTGCTGTCCATC------------CCACAGTCCAAAGTCCTGGCTTTTCCCCAGCAAGTGATGCCCTTCCCC---CAGAGAGACATGCCCCCACAAGCCTTTCTGCTGTACCAGGAGCCT  4

 M  L  T  P  Q  P  L  L  S  I             P  Q  S  K  V  L  P  F  P  Q  Q  V  M  P  Y  P    Q  R  D  M  A  L  Q  A  F  L  L  F  Q  E  P
ATGCTTACTCCTCAGCCACTGCTGTCCATC------------CCACAGTCCAAAGTCCTGCCTTTTCCCCAGCAAGTGATGCCCTACCCC---CAGAGGGACATGGCCCTACAAGCCTTTCTGCTGTTCCAGGAGCCT  5

 M  L  P  T  Q  P  L  L  S  V             P  Q  P  K  V  M  P  F  P  Q  Q  V  M  T  Y  P    Q  R  N  M  P  I  Q  A  L  L  Q  Y  Q  E  P
ATGCTTCCTACTCAGCCCTTGCTCTCTGTT------------CCTCAGCCCAAAGTCATGCCTTTTCCCCAGCAAGTGATGACCTACCCC---CAGAGAAATATGCCCATCCAAGCCCTTCTGCAGTACCAAGAGCCT  6

 R  I  P  S  Q  P  L  P  P  F             F  Q  S  K  V  Q  A  I  P  Q  Q  V  T  P  A  P    Q  R  Y  M  S  L  Q  A  L  H  L  N  Q  Q  P
AGGATTCCTTCTCAGCCCCTGCCACCGTTT------------TTTCAGTCCAAAGTCCAGGCTATTCCCCAGCAAGTGACACCCGCCCCC---CAGAGATACATGTCCCTGCAAGCCCTTCACCTGAACCAGCAACCT  7

 M  L  P  S  Q  P  V  L  S  P             P  Q  S  K  V  A  P  F  P  Q  P  V  V  P  Y  P    Q  R  D  T  P  V  Q  A  F  L  L  Y  Q  D  P
ATGCTTCCTTCTCAGCCTGTGCTATCTCCT------------CCTCAGTCTAAAGTCGCACCTTTTCCTCAGCCTGTGGTGCCCTACCCT---CAGAGAGATACACCTGTCCAAGCTTTTCTGCTGTACCAGGACCCT  8

 M  L  P  S  Q  P  V  L  S  P             P  Q  S  K  V  A  P  F  P  Q  P  V  V  P  Y  P    Q  R  D  T  P  V  Q  A  F  L  L  Y  Q  D  P
ATGCTTCCTTCTCAGCCTGTGCTGTCTCCT------------CCTCAGTCCAAAGTCGCACCTTTCCCTCAGCCTGTGGTGCCCTACCCT---CAGAGAGATACACCTGTCCAAGCTTTTCTGCTGTACCAGGACCCT  9

 M  L  L  S  Q  P  E  L  S  P             P  Q  S  E  V  P  P  F  P  Q  Q  V  V  P  Y  S    Q  R  D  T  P  V  E  A  F  L  L  Y  Q  E  P
ATGCTTCTTTCTCAACCTGAGCTGTCCCCT------------CCTCAGTCCGAAGTCCCACCTTTCCCCCAGCAAGTGGTGCCCTACTCT---CAGAGAGATACACCTGTCGAAGCCTTTCTGCTGTACCAGGAGCCT  10

 M  L  L  S  Q  P  E  L  S  P             P  Q  S  E  V  P  P  F  P  Q  Q  V  V  P  Y  S    Q  R  D  T  P  V  E  A  F  L  L  Y  Q  E  P
ATGCTTCTTTCTCAACCTGAGCTGTCCCCT------------CCTCAGTCCGAAGTCCCACCTTTCCCCCAGCAAGTGGTGCCCTACTCT---CAGAGAGATACACCTGTCGAAGCCTTTTTGCTGTACCAGGAGCCT  11

 M  F  A  P  Q  P  L  L  S  L             P  Q  A  K  V  L  P  V  P  Q  Q  V  V  P  F  P    Q  R  D  M  P  F  Q  A  L  L  L  Y  Q  D  P
ATGTTTGCTCCTCAGCCCCTGCTGTCCCTG------------CCTCAGGCCAAAGTCCTGCCTGTTCCCCAGCAAGTGGTGCCCTTCCCC---CAGAGAGATATGCCCTTCCAGGCCCTTCTGCTCTACCAGGATCCT  12

 M  I  P  P  Q  S  L  L  S  L             S  Q  F  K  V  L  P  V  P  Q  Q  M  V  P  Y  P    Q  R  A  I  P  V  Q  A  V  L  P  F  Q  E  P
ATGATTCCTCCTCAATCCCTGCTGTCCCTT------------TCTCAGTTCAAAGTCCTGCCTGTTCCCCAGCAAATGGTGCCCTACCCC---CAGAGAGCCATACCTGTGCAAGCCGTTCTGCCCTTCCAGGAGCCT  13

 M  I  P  P  Q  S  L  L  S  L             S  Q  F  K  V  L  P  V  P  Q  Q  M  V  P  Y  P    Q  R  A  M  P  V  Q  A  V  L  P  F  Q  E  P
ATGATTCCTCCTCAGTCCCTGCTGTCCCTT------------TCTCAGTTCAAAGTCCTGCCTGTTCCCCAGCAAATGGTGCCCTACCCC---CAGAGAGCCATGCCTGTGCAAGCCCTTCTGCCCTTCCAGGAGCCT  14

 M  F  P  P  Q  P  L  Q  S  L             S  Q  P  K  V  L  P  I  P  Q  Q  V  V  P  Y  L    Q  R  D  M  P  I  Q  A  L  L  L  Y  Q  E  P
ATGTTTCCTCCTCAGCCACTGCAGTCCCTT------------TCTCAGCCCAAAGTCCTGCCTATTCCCCAGCAAGTGGTGCCCTACCTC---CAGAGAGATATGCCCATCCAGGCCCTTCTGCTGTACCAGGAGCCT  15

 M  F  P  P  Q  P  L  P  S  L             S  Q  P  K  V  L  P  I  P  Q  Q  V  V  P  H  I    Q  R  H  M  P  I  Q  A  L  L  L  Y  Q  E  P
ATGTTTCCTCCTCAGCCCCTGCCGTCCCTT------------TCTCAGCCCAAAGTCCTGCCTATTCCCCAGCAAGTGGTGCCCCACATC---CAGAGACATATGCCCATCCAGGCCCTTCTGCTGTACCAGGAGCCT  16

 M  F  P  S  Q  P  L  Q  S  L  P  Q  P  V  S  Q  P  Q  A  L  P  I  P  Q  Q  V  V  P  Y  P    Q  R  E  M  P I/T Q  A  L  L  L  Y  Q  E  P
ATGTTTCCTTCTCAGCCCTTGCAGTCCCTTCCACAGCCCGTTTCTCAGCCCCAAGCCCTGCCTATTCCCCAGCAAGTGGTACCCTACCCA---CAGAGAGAGATGCCCAYCCAGGCCCTTCTGTTGTACCAGGAGCCT  17

 M  F  R  P  Q  S  L  L  S  L             T  Q  S  K  V  L  P  V  P  Q  K  E  V  P  Y  P    Q  R  G  M  P  I  Q  A  F  L  L  Y  Q  Q  P
ATGTTTCGTCCTCAGTCCCTGCTGTCCCTT------------ACTCAGTCCAAAGTCCTGCCAGTTCCTCAGAAAGAGGTGCCCTACCCC---CAGAGAGGTATGCCCATCCAAGCCTTTCTGCTGTACCAGCAGCCC  18

 M  F  P  P  Q  S  A  L  S  V             S  Q  P  K  A  L  P  V  P  Q  K  A  V  S  Y  P    P  R  G  M  P  I  Q  A  F  L  L  Y  Q  E  P
ATGTTTCCTCCTCAGTCCGCGCTGTCCGTT------------TCTCAGCCCAAAGCTCTGCCTGTTCCCCAGAAAGCRGTGTCCTATCCC---CCGAGGGGTATGCCCATCCAGGCCTTTCTGCTGTACCAGGAGCCT  19

 M  L  P  P  Q  S  V  L  S  L             S  Q  P  K  V  L  P  V  P  Q  K  A  V  P  Y  S    Q  R  D  I  P  I  Q  A  F  L  L  Y  Q  E  P
ATGCTTCCTCCTCAGTCCGTGCTGTCTCTT------------TCTCAGCCCAAAGTTCTGCCTGTTCCCCAGAAAGCAGTGCCCTATTCC---CAGAGAGATATACCCATCCAGGCCTTTCTGCTGTACCAGGAGCCT  20
```

```
 M  F  P  P  Q  S  V  L  S  L              S  Q  P  K  V  L  S  V  P  Q  K  A  V  P  Y  P     Q  R  D  M  P  I  Q  A  F  L  L  Y  Q  E  P
ATGTTTCCTCCTCAGTCCGTGCTGTCCCTT------------TCTCAGCCCAAAGTTCTGTCTGTTCCCCAGAAAGCAGTGCCCTATCCC---CAGAGAGATATGCCCATCCAGGCCTTTCTGCTGTATCAGGAGCCT 21

 M  F  P  P  Q  S  V  L  S  L              S  Q  P  K  V  L  S  V  P  Q  K  A  V  P  Y  P     Q  R  E  M  P  I  Q  A  L  L  L  Y  Q  E  P
ATGTTTCCTCCTCAGTCCGTGCTGTCCCTT------------TCTCAGCCCAAAGTTCTGTCTGTTCCCCAGAAAGCAGTGCCCTATCCC---CAGAGAGAGATGCCCATCCAGGCCTTGCTGCTGTACCAGGAGCCT 22

 M  F  P  P  Q  S  V  L  S  L              S  Q  P  K  V  L  S  V  P  Q  K  A  V  P  Y  P     Q  R  E  M  P  I  Q  A  F  L  L  Y  Q  E  P
ATGTTTCCTCCTCAGTCCGTGCTGTCCCTC------------TCTCAGCCCAAAGTTCTGTCTGTTCCCCAGAAAGCAGTGCCCTATCCC---CAGAGAGAGATGCCCATCCAGGCCTTTCTGCTGTACCAGGAGCCT 23

 M  F  P  P  Q  S  V  L  S  L              S  Q  P  K  V  L  S  V  P  Q  K  A  V  P  Y  P     Q  R  D  M  P  I  Q  A  F  L  L  Y  Q  E  P
ATGTTTCCTCCTCAGTCTGTGCTGTCCCTT------------TCTCAGCCCAAAGTTCTGTCTGTTCCCCAGAAAGCAGTGCCCTATCCC---CAGAGAGATATGCCCATCCAGGCCTTTCTGCTGTACCAGGAGCCT 24

 M  F  P  P  Q  S  V  L  S  L              S  Q  P  K  V  L  S  V  P  Q  K  A  V  P  Y  P     Q  R  D  M  P  F  Q  A  F  L  L  Y  Q  E  P
ATGTTTCCTCCTCAGTCCGTGCTGTCCCTT------------TCTCAGCCCAAAGTTCTGTCTGTTCCCCAGAAAGCAGTGCCCTATCCC---CAGAGAGATATGCCCTTCCAGGCCTTTCTGCTGTACCAGGAGCCT 25

 M  F  P  P  Q  S  V  L  S  L              S  Q  S  K  V  L  P  V  P  Q  K  A  V  P  Y  P     Q  R  D  M  P  I  Q  A  F  L  L  Y  Q  E  P
ATGTTTCCTCCTCAGTCCGTGCTGTCCCTT------------TCTCAGTCCAAAGTCCTGCCTGTTCCCCAGAAAGCAGTGCCCTATCCC---CAGAGAGATATGCCCATTCAGGCCTTTCTGCTGTACCAGGAGCCT 26

 M  F  P  P  Q  S  V  L  S  L              S  Q  S  K  V  L  P  V  P  Q  K  A  V  P  Y  P     Q  R  D  M  P  I  Q  A  F  L  L  Y  Q  E  P
ATGTTTCCTCCTCAGTCCGTGCTGTCCCTT------------TCTCAGTCCAAAGTCTTGCCTGTTCCCCAGAAAGCAGTGCCCTATCCC---CAGAGAGATATGCCCATTCAGGCCTTTCTGCTGTACCAGGAGCCT 27

 M  F  P  P  Q  S  V  L  S  L              S  Q  S  K  V  L  P  V  P  Q  K  A  V  P  Y  P     Q  R  D  M  P  I  Q  A  F  L  L  Y  Q  E  P
ATGTTTCCCCCTCAGTCCGTGCTGTCCCTT------------TCTCAGTCCAAAGTTCTGCCTGTTCCCCAGAAAGCAGTGCCCTATCCC---CAGAGAGATATGCCCATTCAGGCCTTTCTGCTGTACCAGGAGCCT 28

 M  F  P  P  Q  S  V  L  S  L              S  Q  S  K  V  L  P  I  P  Q  K  A  V  P  Y  P  I  Q  R  D  M  P  I  Q  A  F  L  L  Y  Q  E  P
ATGTTTCCTCCTCAGTCCGTGCTGTCCCTT------------TCTCAGTCCAAAGTTCTGCCTATTCCCCAGAAAGCAGTGCCCTATCCCATTCAGAGAGATATGCCCATTCAGGCCTTTCTGCTGTACCAGGAGCCT 29

 M  F  P  P  Q  S  V  L  S  L              S  Q  P  K  V  L  P  V  P  Q  K  A  V  P  Y  P     Q  R  D  M  P  I  Q  A  F  L  L  Y  Q  E  P
ATGTTTCCTCCTCAGTCCGTGCTGTCCCTT------------TCTCAGCCCAAAGTTCTGCCTGTTCCCCAGAAAGCAGTGCCCTATCCC---CAGAGAGATATGCCCATCCAGGCCTTTCTGCTGTACCAGGAGCCT 30

 M  F  P  P  Q  S  V  L  A  L              S  Q  P  K  V  L  P  V  P  Q  K  A  V  A  Y  P     Q  R  D  M  P  I  Q  A  F  L  L  Y  Q  E  P
ATGTTTCCTCCTCAATCTGTGCTGGCCCTT------------TCTCAGCCCAAAGTTCTGCCTGTTCCCCAGAAAGCAGTGGCCTATCCC---CAGAGAGATATGCCCATCCAGGCCTTTCTGCTGTACCAGGAGCCT 31

 M  F  P  P  Q  S  V  L  S  L              S  Q  P  K  V  L  P  V  P  Q  K  A  V  P              K  R  D  M  P  I  Q  A  F  L  L  Y  Q  E  P
ATGTTTCCTCCTCAGTCCGTGCTGTCCCTT------------TCTCAGCCCAAAGTTCTGCCTGTTCCCCAGAAAGCAGTGCCC---------AAGAGAGATATGCCCATCCAGGCCTTTCTGCTGTACCAGGAGCCT 32

 M  F  P  P  Q  S  V  L  S  L              S  Q  P  K  V  L  P  V  P  Q  K  A  V  P              Q  R  D  M  P  I  Q  A  F  L  L  Y  Q  E  P
ATGTTTCCTCCTCAGTCTGTGCTGTCCCTT------------TCTCAGCCCAAAGTTCTGCCTGTTCCCCAGAAAGCAGTGCCC---------CAGAGAGATATGCCCATCCAGGCCTTTCTGCTGTACCAGGAGCCT 33

 M  F  P  P  Q  S  V  L  S  L              S  Q  P  K  V  L  P  V  P  Q  K  A  V  P              Q  R  D  M  P  I  Q  A  F  L  L  Y  Q  E  P
ATGTTTCCTCCTCAGTCCGTGCTGTCCCTT------------TCTCAGCCCAAAGTTCTGCCTGTTCCTCAGAAAGCAGTGCCC---------CAGAGAGATATGCCCATCCAGGCCTTTCTGCTGTACCAGGAGCCT 34

 M  F  P  P  Q  S  V  L  S  L              S  Q  P  K  V  L  P  V  P  Q  K  A  V  P              Q  R  D  M  P  I  Q  A  F  L  L  Y  Q  E  P
ATGTTTCCTCCTCAGTCCGTGCTGTCCCTT------------TCTCAGCCCAAAGTTCTGCCTGTTCCTCAAAAAGCAGTGCCC---------CAGAGAGATATGCCCATCCAGGCCTTTCTGCTGTACCAGGAGCCT 35

 M  F  P  P  Q  S  V  L  S  L              S  Q  P  K  V  L  P  V  P  Q  K  A  V  S              Q  R  D  M  P  I  Q  A  F  L  L  Y  Q  E  P
ATGTTTCCTCCTCAATCCGTGCTGTCCCTT------------TCTCAGCCCAAAGTTCTGCCTGTTCCCCAGAAAGCAGTGTCC---------CAGAGAGATATGCCCATCCAGGCCTTTCTGCTGTACCAGGAGCCT 36

 M  F  P  P  Q  S  V  L  S  L              S  Q  P  K  V  L  P  V  L  Q  K  A  V  P              Q  R  D  M  P  V  Q  A  F  L  L  Y  Q  E  P
ATGTTTCCTCCTCAGTCCGTGCTGTCCCTT------------TCTCAGCCCAAAGTTCTGCCTGTTCTCCAGAAAGCAGTGCCC---------CAGAGAGATATGCCCGTCCAGGCCTTTCTGCTGTACCAGGAGCCT 37

 M  F  P  P  Q  S  V  L  S  I              S  Q  P  K  V  L  P  V  P  Q  K  A  V  P              Q  R  D  M  P  I  Q  A  F  L  L  Y  Q  E  P
ATGTTTCCTCCTCAGTCCGTGCTGTCCATT------------TCTCAGCCCAAAGTTCTGCCTGTTCCCCAGAAAGCAGTGCCC---------CAGAGAGATATGCCCATCCAGGCCTTTCTGCTGTACCAGGAGCCT 38

 M  F  P  P  Q  S  M  L  S  L              S  Q  P  K  V  L  P  V  P  Q  K  A  V  P              Q  R  D  M  P  I  Q  A  F  L  L  Y  Q  E  P
ATGTTTCCTCCTCAGTCCATGCTGTCCCTT------------TCTCAGCCCAAAGTTCTGCCTGTTCCCCAGAAAGCAGTGCCC---------CAGAGAGATATGCCCATCCAGGCCTTTCTGCTGTACCAGGAGCCT 39

 M  F  P  P  Q  S  V  L  S  L              S  Q  P  K  V  L  P  V  P  Q  K  A  V  P              Q  R  D  M  P  I  Q  A  F  L  L  Y  Q  E  P
ATGTTTCCTCCTCAGTCCGTGCTGTCCCTT------------TCTCAGCCCAAAGTTCTGCCTGTTCCCCAGAAAGCAGTGCCC---------CAGAGAGATATGCCCATCCAGGCCTTTCTGCTGTACCAGGAGCCT 40

 M  F  P  P  Q  S  V  L  S  L              S  Q  P  K  V  L  P  V  P  Q  K  A  V  P              Q  R  D  M  P  I  Q  A  F  L  L  Y  Q  E  P
ATGTTTCCTCCTCAGTCCGTACTGTCCCTT------------TCTCAGCCCAAAGTTCTGCCTGTTCCCCAGAAAGCAGTGCCC---------CAGAGAGATATGCCCATCCAGGCCTTTCTGCTGTACCAGGAGCCT 41
```

```
 M  F  P  P  Q  S  V  L  S  L              S  Q  P  K  V  L  P  V  P  Q  K  A  V  P           Q  R  D  M  P  I  Q  A  F  L  L  Y  Q  E  P
ATGTTTCCTCCTCAGTCCGTACTGTCCCTT------------TCTCAGCCCAAAGTTCTGCCTGTTCCCCAGAAAGCAGTGCCC---------CAGAGAGATATGCCCATCCAGGCCTTTCTGCTGTACCAGGAGCCT  42

 M  F  P  P  Q  S  V  L  S  L              S  Q  P  K  V  L  P  V  P  Q  K  A  V  P           Q  R  D  M  P  I  Q  A  F  L  L  Y  Q  E  P
ATGTTTCCTCCTCAGTCTGTGCTGTCCCTT------------TCTCAGCCCAAAGTTCTGCCTGTTCCCCAGAAAGCAGTGCCC---------CAGAGAGATATGCCCATCCAGGCCTTTCTGCTGTACCAGGAGCCT  43

 M  F  P  P  Q  S  V  L  S  L              S  Q  P  K  V  L  P  V  P  Q  K  A  V  P           Q  R  D  M  P  I  Q  A  F  L  L  Y  Q  E  P
ATGTTTCCTCCTCAGTCCGTGCTGTCCCTT------------TCTCAGCCCAAAGTTCTGCCTGTTCCCCAGAAAGCAGTGCCC---------CAGAGAGATATGCCCATCCAGGCCTTTCTGCTGTACCAGGAGCCT  44

 M  F  P  P  Q  S  M  L  S  L              S  Q  P  K  V  V  P  V  P  Q  K  A  V  P           Q  R  D  M  P  I  Q  A  F  L  L  Y  Q  E  P
ATGTTTCCTCCTCAGTCCATGCTGTCCCTT------------TCTCAGCCCAAAGTTGTGCCTGTTCCCCAGAAAGCAGTGCCC---------CAGAGAGATATGCCCATCCAGGCCTTTCTGCTGTACCAGGAGCCT  45

 M  F  P  P  Q  S  V  L  S  L              S  Q  P  K  V  L  P  V  P  Q  K  A  V  P           Q  R  D  M  P  I  Q  A  F  L  L  Y  Q  E  P
ATGTTTCCTCCTCAGTCCGTGCTGTCCCTT------------TCTCAGCCCAAAGTTCTGCCTGTTCCCCAGAAAGCAGTGCCC---------CAGAGAGACATGCCCATCCAGGCCTTTCTGCTGTACCAGGAGCCT  46

 M  F  P  P  Q  S  V  L  S  L              S  Q  P  K  V  L  P  V  P  Q  K  A  V  P           Q  R  D  M  P  I  Q  A  F  L  L  Y  Q  E  P
ATGTTTCCTCCTCAGTCCGTGCTGTCCCTT------------TCTCAGCCCAAAGTTCTGCCTGTTCCCCAGAAAGCAGTGCCC---------CAGAGAGATATGCCCATCCAGGCCTTTCTGCTGTACCAGGAGCCT  47

 M  F  P  P  Q  S  L  L  S  L              S  Q  P  K  V  L  P  V  P  Q  K  V  V  P           Q  R  D  M  P  I  Q  A  F  L  L  Y  Q  E  P
ATGTTTCCTCCTCAGTCCCTGCTGTCCCTT------------TCTCAGCCCAAAGTTCTGCCTGTTCCCCAGAAAGTAGTGCCC---------CAGAGAGATATGCCCATCCAGGCCTTTCTGCTGTACCAGGAGCCT  48

 M  F  P  P  Q  S  V  L  S  L              S  Q  P  K  V  L  P  V  P  Q  K A/V V  P           Q  R  D  M  P  I  Q  A  F  L  L  Y  Q  E  P
ATGTTTCCTCCTCAGTCCGTGCTGTCCCTT------------TCTCAGCCCAAAGTTCTGCCTGTTCCCCAGAAAGYAGTGCCC---------CAGAGAGATATGCCCATCCAGGCCTTTCTGCTGTACCAGGAGCCT  49

 M  F  P  P  Q  S  V  L  S  L              S  Q  P  K  V  L  P  V  P  Q  K A/V V  P           Q  R  D  M  P  I  Q  A  F  L  L  Y  Q  E  P
ATGTTTCCTCCTCAGTCCGTGCTGTCCCTT------------TCTCAGCCCAAAGTTCTGCCTGTTCCCCAGAAAGYAGTGCCC---------CAGAGAGATATGCCCATCCAGGCCTTTCTGCTGTACCAGGAGCCT  50
```

1: *Homo sapiens,* AF027807.1; *Symphalangus syndactylus*, XM_055296238.2; **2:** *Canis lupus familiaris*, VSDE02000029.1; *Canis aureus*, XM_077848242.1; *Canis lupus dingo*, XM_049093025.1; **3:** *Felis catus*, NC_058371.1; *Lynx canadensis*, XM_030314494.1; *Acinonyx jubatus*, XM_015074456.2; **4:** *Ailuropoda melanoleuca*, XM_019799951.1; **5:** *Odobenus rosmarus divergens*, XM_004392930.1; **6:** *Rousettus aegyptiacus*, XM_016123356.2; **7:** *Talpa occidentalis*, XM_054687856.1; **8:** *Equus caballus*, XM_070261650.1; *Equus przewalskii*, XM_008541734.2; *Equus quagga*, XM_046655866.1; **9:** *Equus asinus*, FN598778.1; **10:** *Ceratotherium simum simum*, NW_004454157.1; *Ceratotherium simum cottoni*; CM038159.1 **11:** *Diceros bicornis*, JAQIGC010000012.1; **12:** *Sus scrofa*, SSNJ01000008.1; *Phacochoerus africanus*, XM_047798953.1; **13:** *Camelus bactrianus*, OQ730238.1; **14:** *Vicugna pacos*, XM_072941057.1; *Lama glama*, HG969424.1; **15:** *Pseudorca crassidens*, XM_067734634.1; *Delphinapterus leucas*, XM_022561698.1; *Tursiops truncatus*, XM_073804668.1; *Sagmatias obliquidens*, XM_027089638.1; *Stenella coeruleoalba*, XM_079919820.1; *Delphinus delphis*, XM_060013165.1; *Lagenorhynchus albirostris*, XM_060147035.1; **16:** *Balaenoptera*, VNFC03000009.1; *Eschrichtius robustus*, XM_068541978.1; **17:** *Hippopotamus amphibius kiboko*, XM_057726918.1, JASFZM010000013.1; NC_080188.1; *Hippopotamus amphibius amphibius*, JAKVTS010000003.1, PVJP02000899.1, NKPW01003055.1; U53901.1; *Hexaprotodon liberiensis liberiensis*, JAKVTR010000011.1, CAXKWH010018458.1; **18:** *Tragulus javanicus*, PVHZ021082372.1; *Tragulus napu*; U53898; *Tragulus kanchil*, JAIOJX010000025.1; **19:** *Antilocapra americana*, PVKT021585145.1; U53895.1; **20:** *Giraffa camelopardalis*, JABSTO010000045.1; *Giraffa tippelskirchi*, XM_081515766.1; **21:** *Alces alces* OZ291545.1; **22:** *Cervus elaphus* XM_043906556.1, *Dama dama* XM_061145572.1; *Cervus canadensis*, XM_043447622.1; **23:** *Muntiacus reevesi*, OZ005646.2; **24:** *Moschus*, SPDY01005027.1; *Rangifer tarandus*, OZ289225.1; **25**: *Capreolus capreolus*, CATOUS010000074.1; **26:** *Bos taurus*, XM_010806178.3; *Bos indicus*, ON184053.1; *Bison bison bison*, XM_010852144.1; **27:** *Bos grunniens* JN655524.1; *Bos mutus* XM_014480227.2; **28:** *Bubalus bubalis* breed Mediterranean, KC577240.2; *Bubalus bubalis* breed Murrah, DQ631829.1; **29:** *Syncerus caffer*, CADEAB010000138.1; SJXX02015445.1; **30:** *Aepyceros melampus*, SJXQ01000110.1; **31**: *Neotragus moschatus*, SJYA01000952.1; **32:** *Oryx dammah*, XM_040237323.1; **33:** *Alcelaphus buselaphus*, SJXN01070790.1; **34:** *Redunca redunca*, SJYQ01003698.1; **35:** *Kobus ellipsiprymnus*, SJYR01004216.1; **36:** *Philantomba maxwellii*, SJYS01013238.1; **37:** *Cephalophula zebra*, JBSLPB010000008.1; **38:** *Cephalophus harveyi*, SJYT01022683.1; **39:** *Sylvicapra grimmia*, SJYH01002021.1; **40:** *Nanger dama ruficollis*, JAHTZZ010000015.1; **41:** *Eudorcas thomsonii*, SJYE01003710.1; **42:** *Oreotragus oreotragus*, SJYX01004298.1; **43:** *Saiga tatarica* PVIK010165690.1; **44:** *Litocranius walleri*, SJYG01000210.1; **45:** *Rupicapra rupicapra*, OZ022275.1; **46:** *Budorcas taxicolor*, XM_052641775.1; *Pantholops hodgsonii*, JBBYXG010000006.1; *Oreamnos americanus*, WJNR01000345.1; *Nilgiritragus hylocrius*, PVJR01002583.1; **47:** *Ovibos moschatus*, JBFTXH010000007.1; *Ovis*

orientalis, JACSDQ010000006.1; *Ovis vignei*, JBRERX010001131.1; *Ovis ammon polii*, JAKZEL010000006.1; *Ovis nivicola*, CAFBRR010000237.1; *Ovis canadensis*, XM_069595033.1; *Capra pyrenaica*, SRA: SRR8437785 **48:** *Capra ibex*, SJYO01041852.1, *Capra sibirica*, NIYN02007310.1; **49:** *Capra hircus*, JXYW01067838.1; AF409096.1; *Capra aegagrus,* CBYH010035613.1, JXYW01067823.1; *Capra nubiana*, CM141908.1; JBTVNK010000008.1; *Capra falconeri*, JAWPPH010116769.1; SRA:SRR6246090; **50:** *Ovis aries*, MZ542555.1; MW382180.1.

Supplementary Figure S1. Comparative alignment of nucleotide sequences from the partial exon 7 region of the *CSN2* gene and corresponding deduced amino acid sequences across representative mammalian taxa. Gaps indicate the absence of codon segments, whereas grey shading indicates the amino acid residue at position 177 of the mature ruminants β-casein protein. Taxa are grouped according to phylogenetic affinity to facilitate comparison of coding structural variation, including lineage-specific insertion/deletion events, across *Bovidae* and selected mammalian outgroups.
