# Supplementary material for: The Bovidae CSN2 Locus as a Multilayered Evolutionary System: Evidence from Retroposons, Trans-Species Variation and Ancient Recombination
Source: Animals (Basel). 2026 Aug 10;16(16):2482. doi: 10.3390/ani16162482 (PMC13508899; doi:10.3390/ani16162482)
Supplement: Supplementary file 1 [file animals-16-02482-s001.zip › Supplementary Figure S2.pdf]

```
 M  K  V  L  I  L  A  C  L  V  A  L  A  I14 A
ATGAAGGTCCTCATCCTTGCCTGTCTGGTGGCTCTGGCCATTGCA  goat, sheep
                                        L14
---------------------------------------C-----  goat, sheep, mouflon, takin
---------------------------------------C----G  impala
-----------------------C--------------TC-----  saiga antelope
            M5
--------------G------------------------C-----  sumatran serow, muskox
-----------------------C---------------C-----  cattle, buffalo, zebu, yak, deer, gemsbok, antelope, bison, pronghorn
-----------------------C-----------T--TC-----  new-old-world camels, bear; bat; gibbon; panda
-----------------C-----C-----------T--TC-----  man, chimpanzee, panther, cat, jaguar, hyena
--------------------T--C-----------T--TC-----  sloth
-----------------------CT----------T--TC-----  lemur
-----------------------CT----------T---C-----  whale, dolphin
-----------------------C-----------T---C-----  hippopotamus
      I3
------A----------------C-----------T--TC-----  horse, donkey, rhinoceros
      L3                 F9
------C----------------CT----------T---C-----  pig
         F4
---------T-------------C-----------T--TC-----  badger, otter
--------TT-------------C--T--------T--TC-----  dog
```

Supplementary Figure S2. Alignment of nucleotide sequences from the *CSN2* signal peptide coding region across representative mammalian taxa. Dashes indicate identity with the caprine reference sequence. The SNP AJ011018.3:g.3794A>C (highlighted in grey) results in an Ile→Leu substitution at position −2 of the signal peptide. Comparative analysis across mammals supports leucine as the predominant ancestral state, whereas isoleucine represents a derived variant segregating within *Capra* and *Ovis*. Additional substitutions reflect lineage-specific variation within an otherwise conserved signal peptide region.
