# Supplementary material for: The Bovidae CSN2 Locus as a Multilayered Evolutionary System: Evidence from Retroposons, Trans-Species Variation and Ancient Recombination
Source: Animals (Basel). 2026 Aug 10;16(16):2482. doi: 10.3390/ani16162482 (PMC13508899; doi:10.3390/ani16162482)
Supplement: Supplementary file 1 [file animals-16-02482-s001.zip › Supplementary Figure S3.pdf]

~60–55 Ma (Paleocene)

- ► Mammalia
- ► Perissodactyla (non-artiodactyl outgroup) ○ □ △ ◇ +10bp (Val177; +2 aa)
  - Equidae
    - Equus
      - Equus asinus
      - Equus caballus
      - Equus quagga
  - Rhinocerotidae
    - Ceratotherium simum simum
    - Diceros bicornis
- ► Cetartiodactyla ○ □ △ ◇ +10
  - ► Suina
    - Suidae
      - Sus scrofa
        (plesiomorphic non-ruminant state)
  - ► Tylopoda
    - Camelidae
  - ► Whippomorpha
    - Hippopotamidae
      - Hippopotamus amphibius
  - ► Ruminantia
    - ★ Origin of R0 (Bov-tA)
      (derived insertion following divergence of non-ruminants)
    - ★ Emergence of Ala177
      (derived state predominant in Ruminantia)
      Val177/Ala177 retained as a trans-specific polymorphism in Capra and Ovis

      ● □ △ ◇ +10
    - ► Tragulina
      - Tragulidae
        - Tragulus javanicus
          (plesiomorphic ruminant state; basal Ruminantia outside Pecora)
    - ► Pecora (~40–35 Ma)
      - ► Giraffidae
        - Giraffa camelopardalis
          (plesiomorphic state of Pecora)
      - ► Antilocapridae
        - Antilocapra americana
      - ~30–28 Ma ★ Origin of R1 (Bov-A2)
      - ~27–25 Ma → Cervidae ↔ (Moschidae + Bovidae) divergence

        ● ■A2 △ ◇ +10
      - ► Cervidae
        - ~18–16 Ma early cervid radiation
        - ~15–13 Ma Cervinae ↔ Capreolinae split
        - Subfamily Cervinae
          - Tribe Cervini
            - Cervus elaphus
          - Tribe Muntiacini
            - Muntiacus reevesi
        - Subfamily Capreolinae

```
│    │
│    ├── Tribe Alceini
│    │   └── Alces alces
│    │
│    ├── Tribe Capreolini
│    │   └── ~6–5 Ma Capreolus
│    │        ├── Capreolus pygargus
│    │        └── Capreolus capreolus
│    │
│    └── Tribe Rangiferini
│         └── ~4–3 Ma Rangifer
│              └── Rangifer tarandus
│
└── ~23–20 Ma Moschidae–Bovidae divergence
     │
     ├─▶ Moschidae
     │   └── Moschus moschiferus
     │
     └─▶ Bovidae (~20–18 Ma)
          │
          ├── ~15–13 Ma Divergence of the major subfamilies
          │
          │ ★ Origin of R2 (Bov-A2)
          │
          │  ● ■A2 ▲ ◇ +10bp
          │
          ├─▶ Bovinae
          │    │
          │    ├── ~10–9 Ma African–Asian radiation
          │    │
          │    ├── ~8–7 Ma Bos–Bison split
          │    │    │
          │    │    ├── Bison spp.
          │    │    │   └── Bison bison
          │    │    │
          │    │    └── Bos primigenius
          │    │         └── Bos taurus / Bos indicus / Bos grunniens / Bos mutus / Bos javanicus
          │    │
          │    └── ★ L1_BT insertion (CSN2 intron 1)
          │              (synapomorphy of the buffalo clade)
          │         │
          │         ├── ~9–8 Ma → Syncerus divergence
          │         │    └── Syncerus caffer
          │         │
          │         └── ~8–7 Ma → Bubalus divergence
          │              │
          │              ├── Bubalus depressicornis
          │              │
          │              ├── ~3–2 Ma Bubalus arnee
          │              │
          │              └── ~0.008–0.006 Ma Bubalus bubalis
          │
          ├─▶ Antilopinae
          │    │
          │    ├── Aepyceros melampus
          │    ├── Neotragus moschatus
          │    ├── Saiga tatarica (early origin of −2 aa deletion)
          │    │
          │    ├── Oreotragus oreotragus
          │    │
          │    └── Tribe Antilopini
          │         ├── Litocranius walleri
          │         ├── Eudorcas thomsonii
          │         └── Nanger dama
          │
          │  ● ■A2 ▲ ◇ +10bp −2 aa
          │
          ├─▶ Reduncinae
          │    │
          │    ├── Redunca redunca
          │    └── Kobus ellipsiprymnus
          │
          ├─▶ Cephalophinae
          │    │
          │    ├── Cephalophus zebra
          │    ├── Cephalophus harveyi
          │    ├── Philantomba maxwellii
          │
```

```
│  └── Sylvicapra grimmia
│
├─► Alcelaphinae
│  └── Alcelaphus buselaphus
│
├── ★ Origin of R3 (Bov-B)
│
│  ● ■A2 ▲ ◆ +10bp −2 aa
│
├─► Hippotraginae
│
│  ├── Oryx gazella
│  ├── Oryx dammah
│  └── Hippotragus equinus
│
└─► Caprinae
 Predominant inferred ancestral configuration: ● ■A2 ▲ ◆ +10bp −2 aa
  │
  ├── ~10–9 Ma Basal caprine divergence                    │
  ├── Stem caprine lineages
  │   │
  │   └── Pantholopini
  │       │
  │       └── Pantholops hodgsonii
  │
  └─► Crown Caprinae
      │
      ├── ~10–9 Ma Basal rupicolous caprine divergence
      │
      ├─► Archaic rupicolous clade (Ovibovini)
      │   │
      │   ├── Ovibos ● ■A2 △ ◆ +10bp −2 aa
      │   │
      │   └── Clade Budorcas–Capricornis ● ■A2 △ ◆ +10bp −2 aa
      │       │
      │       ├── Budorcas taxicolor
      │       │
      │       └── Capricornis
      │           └──C. sumatraensis
      │
      ├──Pre-Capra/Ovis rupicolous clade
      │
      ├── ★ −10 bp InDel
      │    │
      │    ● ■A ▲ ◆ -10bp −2 aa
      │    │
      │    ├── Oreamnos americanus
      │    ├── Ammotragus lervia
      │    ├── Nilgiritragus hylocrius
      │    └── Rupicapra rupicapra  ● ■A2 ▲ ◆ +10bp −2 aa
      │
      ├── ~8–7 Ma Divergence Capra ↔ Ovis
      │ node with ancestral polymorphism: ■A2/+10bp ↔ ■A/−10bp
      │
      └─► Clade Capra–Ovis
          │
          ├── ★ (Leu-2/Ile) CSN2 leader peptide polymorphism
          │ Leu (ancestral mammalian state) → Ile (derived state)
          │ (trans-specific polymorphism in Capra and Ovis)
          │
          ├── Pre-Capra ● ■A2/■A ▲ ◆ ±10bp −2 aa
          │   │
          │   ├── ibex clade  ● ■A ▲ ◆ +10bp −2 aa
          │   │   │
          │   │   ├── Capra ibex
          │   │   ├── Capra sibirica
          │   │   ├── Capra pyrenaica
          │   │   ├── Capra nubiana
          │   │   ├── Capra walie
          │   │   ├── Capra caucasica
          │   │   └── Capra cylindricornis
          │   │
          │   ├── Capra falconeri ● ■A ▲ ◆ ±10bp −2 aa
          │   │
          │   └── clade aegagrus ● ■A ▲ ◆ ±10bp −2 aa
          │
```

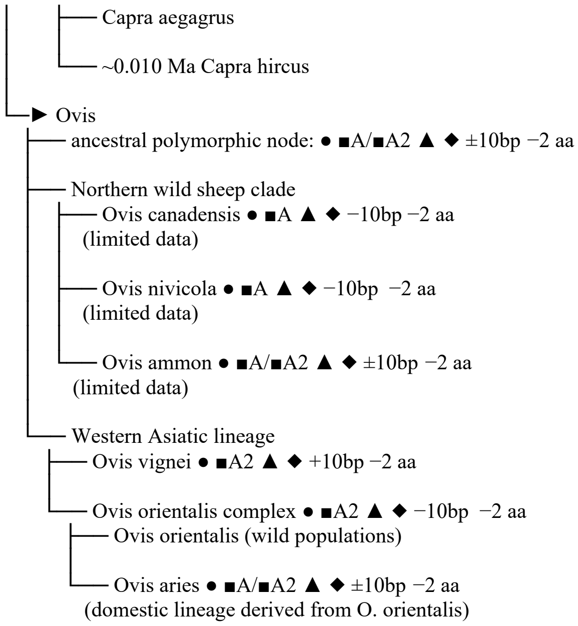


**Supplementary Figure S3.** *Evolutionary reconstruction of CSN2-associated molecular states across Ruminantia and related Cetartiodactyla.*

Simplified phylogenetic framework showing the inferred stepwise emergence and distribution of four diagnostic molecular states (R0–R3), the ±10 bp InDel polymorphism, the two-amino-acid deletion, and associated amino acid variants across major ruminant lineages. The ancestral non-ruminant condition is characterized by the absence of R0–R3, Val177, and the +10 bp state. Emergence of Ala177 and R0 is inferred near the origin of Ruminantia, followed by sequential appearance of R1, R2, and R3 within *Pecora* and *Bovidae*. *Caprinae* retain extensive ancestral polymorphism, including R1+ (Bov-A/Bov-A2) and ±10bp states, with trans-specific maintenance across *Capra* and *Ovis*. The distribution of the two-amino-acid deletion was interpreted by parsimony-based character mapping onto the accepted *Bovidae* phylogeny, representing the minimum number of evolutionary changes required to explain the observed character distribution. Polymorphic nodes indicate incomplete lineage sorting and/or persistence of ancestral variation. Geological divergence times are approximate and expressed in million years ago (Ma). Divergence times are based on published molecular estimates [29–32].

Symbols indicate absence/presence or allelic state of each marker:
○/● = R0−/R0+ (Bov-tA);
□ = R1−; ■A = R1+ (Bov-A); ■A2 = R1+ (Bov-A2);
△/▲ = R2−/R2+ (Bov-A2);
◇/◆ = R3−/R3+ (Bov-B).
