# Supplementary material for: The Bovidae CSN2 Locus as a Multilayered Evolutionary System: Evidence from Retroposons, Trans-Species Variation and Ancient Recombination
Source: Animals (Basel). 2026 Aug 10;16(16):2482. doi: 10.3390/ani16162482 (PMC13508899; doi:10.3390/ani16162482)
Supplement: Supplementary file 1 [file animals-16-02482-s001.zip › Supplementary Table S1.pdf]

Order	Family	Subfamily	Representative taxa	No. of species	No. of *CSN2* records analysed
Cetartiodactyla	*Bovidae*	*Caprinae*	*Capra, Ovis, Rupicapra, Ammotragus, Pseudois, Budorcas, Ovibos, Capricornis, Oreamnos, Nilgiritragus…*	20	187
		Bovinae	*Bos, Bubalus, Bison, Syncerus*	9	71
		Antilopinae	*Oryx, Nanger, Eudorcas, Aepyceros, Litocranius…*	6	6
		Hippotraginae	*Hippotragus*	1	1
		Reduncinae	*Redunca, Kobus*	2	2
	Giraffidae	—	*Giraffa*	1	1
	Cervidae	*Cervinae*	*Cervus, Muntiacus*	2	2
		Capreolinae	*Capreolus, Alces, Rangifer*	3	7
	Moschidae	—	*Moschus*	1	1
	Camelidae	—	*Camelus*	1	1
	Suidae	—	*Sus*	1	1
	Hippopotamidae	—	*Hippopotamus*	1	1
Perissodactyla	*Equidae*	—	*Equus*	3	3
	Rhinocerotidae	—	*Ceratotherium, Diceros*	2	2

Part A.

Capra hircus (Goat)		
**GeneBank acc.n.**	**Position nt (orientation)**	**Breed**
JBBJUA010000004.1	85901437 – 85912232 - (reverse)	Xinong Saanen
JAIWQT010000026.1	85724474 – 85735269 - (reverse)	
JBKFXY010000026.1	87382247 – 87393026 - (reverse)	Boer
CBDTXY010000007.1	85732548 – 85743327 - (reverse)	
JACWUT010000006.1	86104951 – 86115730 - (reverse)	Saanen
JBKFYH010000026.1	86243264 - 86254043 - (reverse)	Guanzhong
CBDTXO010000006.1	86082343 – 86093122 - (reverse)	Rove
JBKFYE010000026.1	86575729 – 86586513 - (reverse)	Nubian
JBFPJL010000026.1	86366109 – 86376893 - (reverse)	
JBKFYD010000026.1	85718212 – 85728994 - (reverse)	
JBKFYC010000026.1	86308778 – 86319562 - (reverse)	Hainan black
CBDTXK010000007.1	86064952 – 86075730 - (reverse)	Poitevine
AJ011019.1	1 – 10785 - (forward)	Garganica
CBDTXQ010000006.1	86068027 – 86078822 - (reverse)	Provencale
AJ011018.3	1- 10786 - (forward)	Garganica
CBDUDO010000006.1	89037829 – 89048619 - (reverse)	Fosses
CBDTZD020000007.1	86142699 – 86153478 - (reverse)	Pyreneenne
JBLKYB010000026.1	88203980 – 88214772 - (reverse)	Hainan black
JBKFYB010000026.1	87419297 – 87430089 - (reverse)	
CBDUDM010000006.1	85944199 – 85954987 - (reverse)	Saanen
CBDTYD010000006.1	86411945 – 86422741 - (reverse)	Creole
JBKFYA010000026.1	86567127 – 86577919 - (reverse)	Shannan White
JBKFYG010000026.1	86210347 – 86221140 - (reverse)	
JBLKYA010000026.1	87533548 – 87544341 - (reverse)	
JBKFXZ010000026.1	85997981 – 86008774 - (reverse)	
JBKFXW010000026.1	95760754 – 95771546 - (reverse)	Tibetan
JBKFXV010000026.1	89492500 – 89503292 - (reverse)	
JBJNUV010000026.1	91460624 – 91471416 - (reverse)	
LWLT01000006.1	86006170 – 86016963 - (reverse)	San Clemente
JBKFXX010000026.1	85428877 – 85439670 - (reverse)	Boer
JBFPJM010000026.1	87509944 – 87520737 - (reverse)	
AJPT02026392.1	567419 – 578211 - (reverse)	Yunnan black
QWFW01031295.1	86006255 – 86017048 - (reverse)	Pashmina (Cashmere)
JBGRUL010000026.1	86572938 – 86583730 - (reverse)	Shanbei (Cashmere)
AF409096.1	2846 – 13636 - (forward)	Alpine/Saanen
JBKFYK010000026.1	90676023 – 90686816 - (reverse)	Alpine
JBKFYJ010000026.1	79933445 – 79944238 - (reverse)	
JBFPJJ010000026.1	92601394 – 92612187 - (reverse)	
JBFPJK010000026.1	86532861 – 86543654 - (reverse)	Guanzhong
JBKFYI010000026.1	86384197 – 86394990 - (reverse)	
XM_005681721.2	cDNA- (forward)	San Clemente
XM_013964699.2	cDNA - (forward)	
Ovis aries (Sheep)		
**GeneBank acc.n.**	**Position nt**	**Breed**
JAKJQL010000023.1	87299080 – 87309861 - (reverse)	Texel
JAJTAA010000158.1	8180920 -8191701 - (forward)	
JAJSZZ010000179.1	2079551 – 2090332 - (forward)	
JAKJQJ010000023.1	86511982 – 86522763 - (reverse)	Charollais
JAJTAB010000446.1	832292 – 843073 - (reverse)	
JAKJQK010000023.1	87348195 – 87358976 - (reverse)	Yunnan
JAJTAR010000025.1	33353003 – 33363784 - (reverse)	
JAKJQH010000023.1	87909858 – 87917681 - (reverse)	Suffolk
JAJTAL010000208.1	6621240 – 6629063 - (reverse)	
CBDTYA010000006.1	88330393 – 88338234 - (reverse)	Ile de France
JAAFGP010000006.1	89120647 – 89128469 - (reverse)	Hu
CBDTXR010000006.1	86290994 – 86298818 - (reverse)	Corse
JAHUUQ010000411.1	86502540 – 86510361 - (reverse)	Romanov x White Dorper
JAKJQO010000023.1	87193954 – 87201768 - (reverse)	Chinese Merino
JAJTAD010000031.1	653137 – 660959 - (reverse)	
JAJTAE010000140.1	29465261 – 29473075 - (reverse)	
JAKJQG010000023.1	86759486 – 86767300 - (reverse)	Romney
JAJTAK010000097.1	133621 – 141435 - (reverse)	
JAKFGC010000037.1	80607705 – 80615528 - (reverse)	East Friesian
JAJTAO010000125.1	2567698 – 2575509 - (reverse)	Ujimqin
JAKJQI010000023.1	88030418 – 88038228 - (reverse)	
JAJTAN010000028.1	88029660 – 88037470 - (reverse)	
JAJTAG010000077.1	7054377 to 7065133 - (forward)	Kermani
CM043057.1	32196918 – 32207686 - (forward)	Ovis aries x Ovis ammon polii
CBDUAG030000006.1	89200500 – 89189567 - (reverse)	Soay
JBPCZR010000006.1	86451350 – 86440417 - (reverse)	Butuo black
JBDLWQ010000006.1	89674487 – 89663554 - (reverse)	Awassi

JAWMPZ010000006.1	91333801 – 91322868 - (reverse)	East Friesian
JAGTAQ010000006.1	86445403 – 86434472 - (reverse)	
JAKFGD010000057.1	31921525 – 31932456 - (reverse)	
JAMHGE010000023.1	87218528 – 87207595 - (reverse)	Waggir
JAMHGD010000007.1	32501116 - 32512049 - (forward)	
JAMHGC010000249.1	7563969 – 7553037 - (reverse)	
JAMFTJ010000486.1	2488833 – 2499766 - (forward)	Romanov
JAMFTI010000172.1	3905140 – 3894208 - (reverse)	
JAMFTK010000023.1	87483157 – 87472225 - (reverse)	
JAKJQP010000023.1	87472350 – 87461417 - (reverse)	Kazak
JAJTAT010000007.1	18905959 – 18916892 - (forward)	
JAJTAU010000086.1	11023008 – 11033941 - (forward)	
JAKJQN010000023.1	87340105 -87329172 - (reverse)	Kermani
JAJTAF010000009.1	87185282 -87174349 - (reverse)	
JAJTAJ010000087.1	68888 – 57955 - (reverse)	Romney
CBDTXW010000007.1	86300585 – 86289651 - (reverse)	Basco Bearnaise
PEKD01002087.1	406249 -395318 - (reverse)	Rambouillet
JAEVFA010000137.1	86213726 – 86202793 - (reverse)	
CBDUAQ010000007.1	86213411 – 86202478 - (reverse)	
JAKJQM010000637.1	88419002 – 88408072 - (reverse)	Polled Dorset
JAJTAV010000101.1	11926929 – 11915999 - (reverse)	
JAJTAW010000052.1	32356726 – 32367656 - (forward)	
JAKJQF010000023.1	87609569 – 87598638 - (reverse)	Qiaoke
JAJTAH010000108.1	1057703 -1046772 - (reverse)	
JAJTAI010000001.1	87616601 – 87605670 - (reverse)	
JAKJQE010000023.1	87702514 – 87691584 - (reverse)	White Dorper
JAGTXJ010000043.1	58556928 – 58545997 - (reverse)	
JAJTAQ010000706.1	348301- 337369 - (reverse)	
JAJTAP010000614.1	1969909 – 1980839 - (forward)	
JAJTAS010000778.1	314190 – 303259 - (reverse)	Yunnan
JAJTAM010000338.1	4660225 - 4671156 - (forward)	Suffolk
JAJTAC010001164.1	269837 – 280768 - (forward)	Charollais
JAHUUR010000810.1	86596235 – 86585302 - (reverse)	Romanov x White Dorper
CBDTXU010000006.1	85979520 – 85968588 - (reverse)	Blanche du Massif Central
CBDTXI010000007.1	86204292 – 86193361 - (reverse)	Noire du Velay
JBEJUG010000024.1	87337183 – 87326242 - (reverse)	Guide Black-Fur
CBDTXP020000023.1	86935675 – 86924742 - (reverse)	Blackbelly
CBDUAK010000007.1	86195949 – 86185019 - (reverse)	Manex tête noire
AMGL02018602.1	144748 – 133813 - (reverse)	Texel
JBLGTL010000006.1	86164688 – 86153757 - (reverse)	Pedi
CBDTZT020000003.1	90228841 – 90217910 - (reverse)	Lacaune
CBDTYB010000006.1	86319020 – 86308081 - (reverse)	Vendeen
CBDUDN010000007.1	85812445 – 85801508 - (reverse)	Ouessant
	**Other species**	
**GeneBank acc.n.**	**Position nt**	**Species**
CM141908.1	32531027 – 32541809 - (forward)	*Capra nubiana* (Nubian ibex)
NIYN02007310.1	8247803 – 8256225 - (reverse)	*Capra sibirica* (Siberian ibex)
CM141940.1	32553176 - 32563871- (forward)	*Capra nubiana* (Nubian ibex)
SJYO01041852.1	71561595 – 71570017 - (reverse)	*Capra ibex* (Alpine ibex)
JXYW01067823.1	18461 – 29185 - (reverse)	*Capra aegagrus* (Bezoar ibex)
CBYH010035613.1	252085 – 262872 - (reverse)	
NIZD01172036.1	3 - 8000	*Pseudois nayaur* (Bharal)
JAWPPH010062006.1<br>JAWPPH010382278.1<br>JAWPPH010116769.1	1 – 4594 - (reverse)<br>1 – 1041 - (reverse)<br>1939 – 6397 - (reverse)	*Capra falconeri* (Markhor)
WJNR01000345.1	30683242 – 30694004 - (forward)	*Oreamnos americanus* (Mountain goat)
PVJR01002583.1	24427 – 35189 - (forward)	*Nilgiritragus hylocrius* (Nilgiri tahr)
NIVO01024987.1	452004 – 461589 - (reverse)	*Ammotragus lervia* (Barbary sheep)
JAXIOT010000008.1	88003376 – 87992883 - (reverse)	*Capricornis sumatraensis* (Sumatran serow)
JAKGDW010000006.1	85564630 – 85575124 - (reverse)	*Budorcas taxicolo* (Takin)
JBFTXH010000007.1	31954239 – 31964734 - (forward)	*Ovibos moschatus* (Muskox)
CATOUS010000074.1	28137696 – 28147769 - (reverse)	*Capreolus capreolus*

		(Roe deer)
CACTHY010000059.1	944978 – 955129 - (reverse)	*Rangifer tarandus* (Reindeer)
OZ289225.1	20361431 - 20351304 - (reverse)	
OZ291602.1	18681535 - 18671408 - (reverse)	
SSNK01103504.1	29617 – 39625 - (forward)	*Alces alces* (Moose)
CAXWQY010001075.1	30446938 - 30457015 - (forward)	
OZ291545.1	18822601 - 18812523 - (reverse)	
OZ290786.1	30429794 – 30439873 - (reverse)	
JADEYB020000017.1	17348874 - 17338794 - (reverse)	
JBTNMK010000006.1	30538856 – 30547958 - (reverse)	*Cervus elaphus* (Red deer)
OZ005646.2	19160178 – 19170401 - (reverse)	*Muntiacus reevesi* (Muntjac deer)
JBBYXG010000006.1	32128329 – 32139298 - (forward)	*Pantholops hodgsonii* (Tibetan antelope)
PVIS010002430.1	25290 – 35975 - (forward)	*Ovis canadensis* (Bighorn)
JBGCUV010000006.1	102085609 – 102074856 - (reverse)	
CM025419.1	32167872 – 32178712 - (forward)	*Ovis orientalis* (Asiatic mouflon)
PVHU021072491.1	13604125 – 13614127 - (forward)	*Moschus* (Musk deer)
JBRERX010001131.1	17172910 – 17178866 - (forward)	*Ovis vignei* (Urial)
JBMPHZ010082182.1<br>JBMPHZ010156498.1	1 – 5863 - (reverse)<br>1 – 4818 - (forward)	*Ovis vignei punjabiensis* (Punjab urial)
OZ022275.1	32030503 – 32041369 - (forward)	*Rupicapra rupicapra* (Alpine chamois)
NIWH01032118.1	68049104 – 68059948 - (reverse)	*Ovis ammon* (Argali)
SJYN01003292.1	222394 – 232536 - (forward)	*Oryx gazella* (Gemsbok)
JABAEV010000003.1	84517171 – 84527324 - (reverse)	*Oryx dammah* (Scimitar-horned oryx)
JAEFBM010000003.1	19914499 – 19924670 - (forward)	*Hippotragus equinus* (Roan antelope)
CAFBRR010000237.1	2866059 – 2876739 - (forward)	*Ovis nivicola* (Siberian bighorn)
JAKZEL010000006.1	32488115 – 32477326 - (forward)	*Ovis ammon* (Argali)
JBIQQL010000006.1	86756717 – 86745948 - (reverse)	
JPYT01031076.1	16528 – 6281 - (reverse)	*Bison bison* (American bison)
ON398262.1	1 – 10338 - (forward)	*Bos indicus* x *Bos taurus* (Zebu x Cattle)
ON398263.1	1 – 10338 - (forward)	
X14711.1	1 – 10338 - (forward)	*Bos taurus* (Cattle)
CM117469.1	87083621 – 87095418 - (reverse)	
M55158	1 – 10338 - (forward)	
OZ375899.1	93120426 – 93132201 - (reverse)	
OZ387464.1	101679078 – 101690850 - (reverse)	
CM117959.1	87698907 – 87710679 - (reverse)	
CM079954.1	103432154 – 103443926 - (reverse)	
OY997234.1	86582810 – 86594585 - (reverse)	
CM105374.1	104362441 – 104374216 - (reverse)	
CM079984.1	101788362 - 101800134 - (reverse)	
NC_037333.1	85449103 – 85460875 - (reverse)	
OX344695.1	86621495 – 86631787 - (reverse)	
OZ375839.1	90755534 – 90765826 - (reverse)	
OZ375959.1	101646185 – 101656477 - (reverse)	
OZ375929.1	88162046 – 88172338 - (reverse)	
OZ375869.1	88204439 – 88214731 - (reverse)	
CM054875.2	88136942 – 88147236 - (reverse)	
CM037801.1	85374277 – 85384567 - (reverse)	
CM078296.1	32395474 – 32405763 - (forward)	
LR962889.1	32196058 – 32206346 - (forward)	
CM067183.1	90156096 – 90166410 - (reverse)	
LR962764.1	86231150 – 86241438 - (reverse)	
JBMUHV010000001.1	32163670 – 32173959 - (forward)	
CM038084.1	85360145 – 85370434 - (reverse)	
PRDE01000026.1	87180124 – 87169897 - (reverse)	*Bos indicus* (Zebu)
ON206758.1	1 – 10338 - (forward)	
ON206757.1	1 – 10338 - (forward)	

ON184059.1	1 – 10338 - (forward)	
ON184057.1	1 – 10338 - (forward)	
ON124705.1	1 – 10338 - (forward)	
ON206754.1	1 – 10338 - (forward)	
ON184052.1	1 – 10338 - (forward)	
ON206756.1	1 – 10338 - (forward)	
ON184058.1	1 – 10338 - (forward)	
ON184054.1	1 – 10338 - (forward)	
ON124706.1	1 – 10338 - (forward)	
ON206753.1	1 – 10338 - (forward)	
ON184051.1	1 – 10338 - (forward)	
ON184055.1	1 – 10338 - (forward)	
ON206751.1	1 – 10338 - (forward)	
ON184053.1	1 – 10338 - (forward)	
ON184060.1	1 – 10338 - (forward)	
ON124707.1	1 – 10338 - (forward)	
ON206759.1	1 – 10338 - (forward)	
ON206760.1	1 – 10338 - (forward)	
ON184056.1	1 – 10338 - (forward)	
ON206752.1	1 – 10338 - (forward)	
ON206750.1	1 – 10338 - (forward)	
ON124708.1	1 – 10338 - (forward)	
ON206755.1	1 – 10338 - (forward)	
ON124709.1	1 – 10338 - (forward)	
ON124704.1	1 – 10338 - (forward)	
CM145279.1	32236489 – 32246802 - (forward)	*Bos grunniens* (Domestic Yak)
CM113970.1	32265724 – 32276037 - (forward)	
CM050815.1	31590625 – 31600941 - (forward)	
CM016695.1	35918149 – 35929460 - (forward)	
JAVLEU010000006.1	85750260 – 85740028 - (reverse)	*Bos javanicus* (Banteng)
AGSK01123303.1	18176 – 28414 - (forward)	*Bos mutus* (Wild yak)
VBQZ03000078.1	1049607 – 1051087 - (reverse)	
JANCMR010002070.1	85160359 – 85150062 - (reverse)	
CADEAB010000138.1	32160785 – 32171387 - (forward)	*Syncerus caffer* (Cape buffalo)
SJXX02015445.1	828457 – 839075 - (reverse)	
NC_073630.1	89654172 – 89664785 - (reverse)	*Bubalus bubalis carabanensis* (Swamp type water buffalo)
CM119497.1	91745348 – 91755959 - (reverse)	
JARFXY010000007.1	89654172 – 89664785 - (reverse)	
AWWX01564620.1	1478 – 12016 - (forward)	*Bubalus bubalis bubali* (Water buffalo)
FN424088	1 – 10347 - (forward)	
CBFDFF020000011.1	79628919 – 79639524 - (reverse)	
NC_037551.1	32117159 – 32127763 - (forward)	
JAMXBS010060650.1	20630 – 31245 - (forward)	*Bubalus depressicornis* (Lowland anoa)
SJYG01000210.1	2370336 – 2380598 - (forward)	*Litocranius walleri* (Gerenuk)
SJXQ01000110.1	52872 – 42470 - (reverse)	*Aepyceros melampus* (Impala)
SJYE01003710.1	1040612 – 1050886 - (reverse)	*Eudorcas thomsonii* (Thomson's gazelle)
JAHTZZ010000015.1	32341944 – 32352187 - (forward)	*Nanger dama* (Dama gazelle)
SJYQ01003698.1	142185 – 151516 - (forward)	*Redunca redunca* (Bohor reedbuck)
SJYR01004216.1	55655 – 64879 - (forward)	*Kobus ellipsiprymnus* (Waterbuck)
LVKQ01124240.1	14932 – 24734 - (forward)	*Giraffa camelopardalis* (Giraffe)
PVKT021585145.1	29424702 – 29434215 - (forward)	*Antilocapra americana* (Pronghorn)
PVHZ021082372.1	22046138 – 22056121 - (forward)	*Tragulus javanicus* (Java mouse-deer)
LSZX01093722.1	25640 – 35296 - (forward)	*Camelus dromedarius* (Dromedary)
JBJXUT010000008.1	68885128 – 68895488 - (reverse)	*Sus scrofa* (Pig)
JAKVTS010000003.1	147887625 – 147899486 - (forward)	*Hippopotamus amphibius* (Hippopotamus)
NW_004454157.1	71995431 – 72008191 - (reverse)	*Ceratotherium simum simum* (Southern white rhinoceros)
PVJY021981105.1	1490446 – 1503207 - (reverse)	*Diceros bicornis* (Black rhinoceros)
FN598778.1	1 – 9378 - (forward)	*Equus asinus*

		(Donkey)
JAPJZR010002749.1	51021513 – 51030894 - (forward)	*Equus caballus* (Horse)
JAKJSB010001568.1	101042704 – 101052077 - (forward)	*Equus quagga* (Common Zebra)

Part B

Supplementary Table S1. Taxonomic composition of the dataset and *CSN2* genomic records analysed. Part A: summary of the taxonomic representation of the dataset across the mammalian groups included in this study, reporting the number of species and *CSN2* genomic records analysed for each major taxonomic group. **Part B:** complete list of *CSN2* genomic records retrieved from public databases (GenBank, RefSeq, and WGS assemblies), including accession numbers, genomic coordinates, sequence orientation, and breed (when available) or species information used in the comparative analyses.
