# Supplementary material for: The Bovidae CSN2 Locus as a Multilayered Evolutionary System: Evidence from Retroposons, Trans-Species Variation and Ancient Recombination
Source: Animals (Basel). 2026 Aug 10;16(16):2482. doi: 10.3390/ani16162482 (PMC13508899; doi:10.3390/ani16162482)
Supplement: Supplementary file 1 [file animals-16-02482-s001.zip › Supplementary Table S2.pdf]

Capra hircus (Goat)					
**Alleles**		**Haplotypes (SNP)**	**GeneBank acc.n.**	**Breed**	**Haplotypes (Retroposons/InDel)**
**A-type**	**A**	g.3794A/g.8946C/g.10562C	JBBJUA010000004.1	Xinong Saanen	R0+ (Bov-tA), R1+ (Bov-A), R2+ (Bov-A2), R3+ (Bov-B) InDel +10bp
			JAIWQT010000026.1		
			JBKFXY010000026.1	Boer	R0+ (Bov-tA), R1+ (Bov-A), R2+ (Bov-A2), R3+ (Bov-B), InDel -10bp
			CBDTXY010000007.1		
			JACWUT010000006.1	Saanen	
			JBKFYH010000026.1	Guanzhong	
			CBDTXO010000006.1	Rove	
			JBKFYE010000026.1	Nubian	
			JBFPJL010000026.1		
			JBKFYD010000026.1		
			JBKFYC010000026.1	Hainan black	
			CBDTXK010000007.1	Poitevine	
	**01**		AJ011019.1	Garganica	R0+ (Bov-tA), R1+ (Bov-A), R2+ (Bov-A2), R3+ (Bov-B), InDel +10bp
	**A1**	g.3794C/g.8946C/g.10562T	CBDTXQ010000006.1	Provencale	
	**A2**	g.3794C/g.8946C/g.10562C	AJ011018.3	Garganica	
			CBDUDO010000006.1	Fosses	
			CBDTZD020000007.1	Pyreneenne	R0+ (Bov-tA), R1+ (Bov-A), R2+ (Bov-A2), R3+ (Bov-B), InDel -10bp
**C-type**	**C1**	g.3794C/g.8946T/g.10562T	JBLKYB010000026.1	Hainan black	R0+ (Bov-tA), R1+ (Bov-A), R2+ (Bov-A2), R3+ (Bov-B), InDel +10bp
			JBKFYB010000026.1		
			CBDUDM010000006.1	Saanen	
			CBDTYD010000006.1	Creole	
			JBKFYA010000026.1	Shannan White	
			JBKFYG010000026.1		
			JBLKYA010000026.1		
			JBKFXZ010000026.1		
			JBKFXW010000026.1	Tibetan	
			JBKFXV010000026.1		
			JBJNUV010000026.1		
			LWLT01000006.1	San Clemente	
	**C**	g.3794A/g.8946T/g.10562C	JBKFXX010000026.1	Boer	
			JBFPJM010000026.1		
			AJPT02026392.1	Yunnan black	
			QWFW01031295.1	Pashmina (Cashmere)	
			JBGRUL010000026.1	Shanbei (Cashmere)	
			AF409096.1	Alpine/Saanen	
			JBKFYK010000026.1	Alpine	
			JBKFYJ010000026.1		
			JBFPJJ010000026.1		
			JBFPJK010000026.1	Guanzhong	
			JBKFYI010000026.1		
			XM_005681721.2	San Clemente	
			XM_013964699.2		

Ovis aries (Sheep)					
**Alleles**		**Aplotipo SNP**	**GeneBank acc.n.**	**Breed**	**Aplotipo Retro**
**A-type**	**A2**	g.3794C/g.8946C/g.10562C	JAKJQL010000023.1	Texel	R0+ (Bov-tA), R1+ (Bov-A), R2+ (Bov-A2), R3+ (Bov-B), InDel -10bp
			JAJTAA010000158.1		
			JAJSZZ010000179.1		
			JAKJQJ010000023.1	Charollais	
			JAJTAB010000446.1		
			JAKJQK010000023.1	Yunnan	
			JAJTAR010000025.1		

			JAKJQH010000023.1	Suffolk	
			JAJTAL010000208.1		
			CBDTYA010000006.1	Ile de France	
			JAAFGP010000006.1	Hu	
			CBDTXR010000006.1	Corse	
			JAHUUQ010000411.1	Romanov x White Dorper	
			JAKJQO010000023.1	Chinese Merino	
			JAJTAD010000031.1		
			JAJTAE010000140.1		
			JAKJQG010000023.1	Romney	
			JAJTAK010000097.1		
			JAKFGC010000037.1	East Friesian	
			JAJTAO010000125.1	Ujimqin	
			JAKJQI010000023.1		
			JAJTAN010000028.1		
			JAJTAG010000077.1	Kermani	
			CM043057.1	Ovis aries x Ovis ammon polii	
			CBDUAG030000006.1	Soay	R0+ (Bov-tA), R1+ (Bov-A2), R2+ (Bov-A2), R3+ (Bov-B), InDel +10bp
			JBPCZR010000006.1	Butuo black	
			JBDLWQ010000006.1	Awassi	
			JAWMPZ010000006.1	East Friesian	
			JAGTAQ010000006.1		
			JAKFGD010000057.1		
			JAMHGE010000023.1	Waggir	
			JAMHGD010000007.1		
			JAMHGC010000249.1		
			JAMFTJ010000486.1	Romanov	
			JAMFTI010000172.1		
			JAMFTK010000023.1		
			JAKJQP010000023.1	Kazak	
			JAJTAT010000007.1		
			JAJTAU010000086.1		
			JAKJQN010000023.1	Kermani	
			JAJTAF010000009.1		
			JAJTAJ010000087.1	Romney	
			CBDTXW010000007.1	Basco Bearnaise	
			PEKD01002087.1	Rambouillet	
			JAEVFA010000137.1		
			CBDUAQ010000007.1		
			JAKJQM010000637.1	Polled Dorset	
			JAJTAV010000101.1		
			JAJTAW010000052.1		
			JAKJQF010000023.1	Qiaoke	
			JAJTAH010000108.1		
			JAJTAI010000001.1		
			JAKJQE010000023.1	White Dorper	

			JAGTXJ010000043.1		
			JAJTAQ010000706.1		
			JAJTAP010000614.1		
			JAJTAS010000778.1	Yunnan	
			JAJTAM010000338.1	Suffolk	
			JAJTAC010001164.1	Charollais	
			JAHUUR010000810.1	Romanov x White Dorper	
			CBDTXU010000006.1	Blanche du Massif Central	
			CBDTXI010000007.1	Noire du Velay	
			JBEJUG010000024.1	Guide Black-Fur	
			CBDTXP020000023.1	Blackbelly	
			CBDUAK010000007.1	Manex tête noire	
			AMGL02018602.1	Texel	
			JBLGTL010000006.1	Pedi	
			CBDTZT020000003.1	Lacaune	
			CBDTYB010000006.1	Vendeen	
			CBDUDN010000007.1	Ouessant	
**Other species**					
**Allele**		**Haplotypes (SNP)**	**GeneBank acc.n.**	**Species**	**Haplotypes (Retroposons/InDel)**
**A-type**	**A**	g.3794A/g.8946C/g.10562C	CM141908.1	*Capra nubiana* (Nubian ibex)	R0+ (Bov-tA), R1+ (Bov-A), R2+ (Bov-A2), R3+ (Bov-B), InDel -10bp
**C-type**	**CX**	g.3794C/g.8946T/g.10562C	NIYN02007310.1	*Capra sibirica* (Siberian ibex)	R0+ (Bov-tA), R1+ (Bov-A), R2+ (Bov-A2), R3+ (Bov-B), InDel +10bp
			CM141940.1	*Capra nubiana* (Nubian ibex)	
			SJYO01041852.1	*Capra ibex* (Alpine ibex)	
**A-type**	**A**	g.3794A/g.8946C/g.10562C	JXYW01067823.1	*Capra aegagrus* (Bezoar ibex)	
**C-type**	**C**	g.3794A/g.8946T/g.10562C	CBYH010035613.1		
**A-type**	**A2**	g.3794C/g.8946C/g.10562C	NIZD01172036.1	*Pseudois nayaur* (Bharal)	
			JAWPPH010062006.1<br>JAWPPH010382278.1<br>JAWPPH010116769.1	*Capra falconeri* (Markhor)	R0+ (Bov-tA), R1+ (Bov-A), R2+ (Bov-A2), R3+ (Bov-B), InDel -10bp
			WJNR01000345.1	*Oreamnos americanus* (Mountain goat)	
			PVJR01002583.1	*Nilgiritragus hylocrius* (Nilgiri tahr)	
			NIVO01024987.1	*Ammotragus lervia* (Barbary sheep)	
			JAXIOT010000008.1	*Capricornis sumatraensis* (Sumatran serow)	R0+ (Bov-tA), R1+ (Bov-A2) R2− (Bov-A2), R3+ (Bov-B), InDel +10bp
			JAKGDW010000006.1	*Budorcas taxicolo* (Takin)	
			JBFTXH010000007.1	*Ovibos moschatus* (Muskox)	
			CATOUS010000074.1	*Capreolus capreolus*	R0+ (Bov-tA), R1+ (Bov-A2), R2− (Bov-A2), R3− (Bov-B), InDel +10bp

				(Roe deer)	
			CACTHY010000059.1<br>OZ289225.1<br>OZ291602.1	*Rangifer tarandus*<br>(Reindeer)	
			SSNK01103504.1<br>CAXWQY010001075.1<br>OZ291545.1<br>OZ290786.1<br>JADEYB020000017.1	*Alces alces*<br>(Moose)	
			JBTNMK010000006.1	*Cervus elaphus*<br>(Red deer)	
			OZ005646.2	*Muntiacus reevesi*<br>(Muntjac deer)	
			JBBYXG010000006.1	*Pantholops hodgsonii*<br>(Tibetan antelope)	R0+ (Bov-tA), R1+ (Bov-A2), R2+ (Bov-A2), R3+ (Bov-B), InDel −10bp
			PVIS010002430.1<br>JBGCUV010000006.1	*Ovis canadensis*<br>(Bighorn)	
			CM025419.1	*Ovis orientalis*<br>(Asiatic mouflon)	
			PVHU021072491.1	*Moschus*<br>(Musk deer)	R0+ (Bov-tA), R1+ (Bov-A2), R2− (Bov-A2), R3− (Bov-B), InDel +10bp
			JBRERX010001131.1	*Ovis vignei*<br>(Urial)	R0+ (Bov-tA), R1+ (Bov-A2), R2+ (Bov-A2), R3+ (Bov-B), InDel +10bp
			JBMPHZ010082182.1<br>JBMPHZ010156498.1	*Ovis vignei punjabiensis*<br>(Punjab urial)	
			OZ022275.1	*Rupicapra rupicapra*<br>(Alpine chamois)	
			NIWH01032118.1	*Ovis ammon*<br>(Argali)	
			SJYN01003292.1	*Oryx gazella*<br>(Gemsbok)	
			JABAEV010000003.1	*Oryx dammah*<br>(Scimitar-horned oryx)	
			JAEFBM010000003.1	*Hippotragus equinus*<br>(Roan antelope)	
			CAFBRR010000237.1	*Ovis nivicola*<br>(Siberian bighorn)	R0+ (Bov-tA), R1+ (Bov-A), R2+ (Bov-A2), R3+ (Bov-B), InDel -10bp
			JAKZEL010000006.1<br>JBIQQL010000006.1	*Ovis ammon*<br>(Argali)	
			JPYT01031076.1	*Bison bison*<br>(American bison)	R0+ (Bov-tA), R1+ (Bov-A2), R2+ (Bov-A2), R3− (Bov-B), InDel +10bp
			ON398262.1<br>ON398263.1	*Bos indicus* x *Bos taurus*<br>(Zebu x Cattle)	
			X14711.1<br>CM117469.1<br>M55158	*Bos taurus*<br>(Cattle)	

			OZ375899.1		
			OZ387464.1		
			CM117959.1		
			CM079954.1		
			OY997234.1		
			CM105374.1		
			CM079984.1		
			NC_037333.1		
			OX344695.1		
			OZ375839.1		
			OZ375959.1		
			OZ375929.1		
			OZ375869.1		
			CM054875.2		
			CM037801.1		
			CM078296.1		
			LR962889.1		
			CM067183.1		
			LR962764.1		
			JBMUHV010000001.1		
			CM038084.1		
			PRDE01000026.1	*Bos indicus* (Zebu)	
			ON206758.1		
			ON206757.1		
			ON184059.1		
			ON184057.1		
			ON124705.1		
			ON206754.1		
			ON184052.1		
			ON206756.1		
			ON184058.1		
			ON184054.1		
			ON124706.1		
			ON206753.1		
			ON184051.1		
			ON184055.1		
			ON206751.1		
			ON184053.1		
			ON184060.1		
			ON124707.1		
			ON206759.1		
			ON206760.1		
			ON184056.1		
			ON206752.1		
			ON206750.1		
			ON124708.1		
			ON206755.1		
			ON124709.1		
			ON124704.1		

			CM145279.1	*Bos grunniens* (Domestic Yak)	
			CM113970.1		
			CM050815.1		
			CM016695.1		
			JAVLEU010000006.1	*Bos javanicus* (Banteng)	
			AGSK01123303.1	*Bos mutus* (Wild yak)	
			VBQZ03000078.1		
			JANCMR010002070.1		
			CADEAB010000138.1	*Syncerus caffer* (Cape buffalo)	
			SJXX02015445.1		
			NC_073630.1	*Bubalus bubalis carabanensis* (Swamp type water buffalo)	
			CM119497.1		
			JARFXY010000007.1		
			AWWX01564620.1	*Bubalus bubalis bubali* (Water buffalo)	
			FN424088		
			CBFDFF020000011.1		
			NC_037551.1		
			JAMXBS010060650.1	*Bubalus depressicornis* (Lowland anoa)	
			SJYG01000210.1	*Litocranius walleri* (Gerenuk)	
			SJXQ01000110.1	*Aepyceros melampus* (Impala)	
			SJYE01003710.1	*Eudorcas thomsonii* (Thomson's gazelle)	
			JAHTZZ010000015.1	*Nanger dama* (Dama gazelle)	
			SJYQ01003698.1	*Redunca redunca* (Bohor reedbuck)	
			SJYR01004216.1	*Kobus ellipsiprymnus* (Waterbuck)	
			LVKQ01124240.1	*Giraffa camelopardalis* (Giraffe)	R0+ (Bov-tA), R1− (Bov-A2), R2− (Bov-A2), R3− (Bov-B), InDel +10bp
			PVKT021585145.1	*Antilocapra americana* (Pronghorn)	
**C-type like**	**CX like**	g.3794C/g.8946A/g.10562C	PVHZ021082372.1	*Tragulus javanicus* (Java mouse-deer)	
**C-type**	**CX**	g.3794C/g.8946T/g.10562C	LSZX01093722.1	*Camelus dromedarius* (Dromedary)	R0- (Bov-tA), R1− (Bov-A2), R2− (Bov-A2), R3− (Bov-B), InDel +10bp
			JBJXUT010000008.1	*Sus scrofa* (Pig)	
			JAKVTS010000003.1	*Hippopotamus amphibius* (Hippopotamus)	
			NW_004454157.1	*Ceratotherium simum simum* (Southern white rhinoceros)	
			PVJY021981105.1	*Diceros bicornis* (Black rhinoceros)	
			FN598778.1	*Equus asinus*	

				(Donkey)	
			JAPJZR010002749.1	*Equus caballus* (Horse)	
			JAKJSB010001568.1	*Equus quagga* (Common Zebra)	

Table S2. Genomic sequences analyzed for comparative investigation of the *CSN2 locus*. The table reports accession numbers, genomic coordinates, breed or species information, SNP-defined haplotypes, and associated retroposon/intronic insertion-deletion configurations. Representative allelic lineages are grouped according to A-type and C-type haplotypic backgrounds.
