# Supplementary material for: The Bovidae CSN2 Locus as a Multilayered Evolutionary System: Evidence from Retroposons, Trans-Species Variation and Ancient Recombination
Source: Animals (Basel). 2026 Aug 10;16(16):2482. doi: 10.3390/ani16162482 (PMC13508899; doi:10.3390/ani16162482)
Supplement: Supplementary file 1 [file animals-16-02482-s001.zip › Supplementary Table S3.pdf]

Species	*Capra hircus*	*Capra hircus*	*Capra hircus*	*Capra hircus*	*Capra hircus*	*Capra hircus*	*Capra falconeri*	*Capra aegagrus*	*Capra aegagrus*	*Capra sibirica*	*Ovis aries*	Description
**Genotype**	***CSN2* A2**	***CSN2* 01**	***CSN2 A***	***CSN2* A1**	***CSN2* C**	***CSN2* C1**	***CSN2* A2**	***CSN2* C**	***CSN2* A**	***CSN2* CX**	***CSN2* A2**	
**GenBank n.**	**AJ011018.3**	**AJ011019.1**	**JBBJUA010000004.1<br>JAIWQT010000026.1<br>JBKFXY010000026.1<br>CBDTXY010000007.1<br>JACWUT010000006.1<br>JBKFYH010000026.1<br>CBDTXO010000006.1<br>JBKFYE010000026.1<br>JBFPJL010000026.1<br>JBKFYD010000026.1<br>JBKFYC010000026.1<br>CBDTXK010000007.1**	**CBDTXQ010000006.1**	**JBKFXX010000026.1<br>JBFPJM010000026.1<br>AJPT02026392.1<br>QWFW01031295.1<br>JBGRUL010000026.1<br>AF409096.1<br>JBKFYK010000026.1<br>JBKFYJ010000026.1<br>JBFPJJ010000026.1<br>JBFPJK010000026.1<br>JBKFYI010000026.1<br>XM_005681721.2<br>XM_013964699.2**	**JBLKYB010000026.1<br>JBKFYB010000026.1<br>CBDUDM010000006.1<br>CBDTYD010000006.1<br>JBKFYA010000026.1<br>JBKFYG010000026.1<br>JBLKYA010000026.1<br>JBKFXZ010000026.1<br>JBKFXW010000026.1<br>JBKFXV010000026.1<br>JBJNUV010000026.1<br>LWLT01000006.1**	**JAWPPH010062006.1<br>JAWPPH010382278.1<br>JAWPPH010116769.1**	**CBYH010035613.1**	**JXYW01067823.1**	**NIYN02007310.1**	**X79703.1**	
**Location**	**Mutations**											
24	C	C	A	A	A	A	A	A	A	A	A	
25	T	T	A	A	A	A	A	A	A	A	A	
26	A	A	G	G	G	G	G	G	G	G	G	
28	G	G	A	A	A	A	A	A	A	A	A	
137	G	G	G	G	G	G	C	G	G	C	C	
146	A	A	A	A	A	A	G	A	A	G	G	
315	C	C	C	T	C	T	C	C	C	C	C	
417	T	T	T	A	T	A	T	T	T	T	T	
564	A	A	A	G	A	G	T	A	A	T	T	
695-696	DEL	DEL	T	T	T	T	T	T	T	T	T	
698-699	DEL	DEL	T	T	T	T	T	T	T	T	T	**Promoter**
1037	G	G	G	G	G	G	A	G	G	G	G	
1200	A	A	A	A	A	A	G	A	A	A	A	
1206	G	G	G	G	G/A	G	A	G	A	A	A	
1311	T	C	T	T	T	T	T	T	T	T	T	
1470	A	A	A	A	A	A	G	A	A	G	G	
1526	G	G	G	G	G	G	A	G	G	A	A	
1538	A	G	A	A	A/G	A	A	G	A	A	A	
1624	A	A	G/A	A	A	A	A	A	A	A	A	
1641	C	C	C	C	C	C	T	C	C	C	C	
1644	G	G	C	C	C	C	C	C	C	C	C	
1790	T	T	T/A	T	T	T	T	T	T	T	T	
1860	T	A	T	T	T	T	T	T	T	T	T	
1886	C	T	C	C	C	C	C	C	C	C	C	
1937	G	G	DEL	DEL	DEL	DEL	DEL	DEL	DEL	DEL	DEL	
1945	C	C	A	C	A	A	A	A	A	A	A	
2060	A	C	A	A	A	A	A	A	A	A	T	
2081	G	A	A	G	A	A	A	A	A	A	A	
2166	T	T	T	T	T	T	G	T	T	G	G	
2357	T	C	T	T	T	T	T	T	T	T	T	
2467	C	C	T	T	T	T	T	T	T	T	T	
2522	A	T	T	A	T	A	T	T	T	T	T	
2542	G	G	A	A	A	A	A	A	A	A	A	
2605	G	G	G	G	G	G	A	G	G	A	A	
2662	A	C	C	A	C	A	A	C	C	A	A	
2728	G	G	A	A	A	A	A	A	A	A	A	**Intron 1**
2963	A	G	A	A	A	A	A	A	A	A	A	
3023	G	G	DEL	DEL	DEL	DEL	DEL	DEL	DEL	DEL	DEL	
3129	C	C	C	C	C/T	C	T	C	C	T	T	
3152-3153	DEL	DEL	DEL	DEL	A	A	DEL	DEL	A	DEL	DEL	
3182	C	T	T	C	T	C	C	T	C	C	G	
3237	T	C	C	T	C	T	T	C	C	T	C	
3383	A	T	A	A	A	A	A	A	A	A	A	
3402	A	A	A	G	A	G	G	A	A	G	A	
3520	C	C	C	T	C	T	T	C	C	T	T	
3536	G	G	G	A	G	A	A	G	G	A	A	
3606	C	C	C	C	C	C	T	C	C	C	C	
3607	A	A	A	G	A	G	G	A	A	G	G	
3711	C	A	A	C	A	C	A	A	A	C	A	

3794	C (Leu-2)	A (Ile-2)	A (Ile-2)	C (Leu-2)	A (Ile-2)	C (Leu-2)	C (Leu-2)	A (Ile-2)	A (Ile-2)	C (Leu-2)	C (Leu-2)	**Exon 2**
3814	T	A	A	T	A	T	A	A	A	A	A	
3870	G	A	A	G	A	G	G	A	A	G	G	
3875	G	A	A	G	A	G	G	A	A	A	A	
4135	G	G	DEL	DEL	DEL	DEL	DEL	DEL	DEL	DEL	DEL	
4268	G	G	DEL	DEL	DEL	DEL	DEL	DEL	DEL	DEL	DEL	
4301	C	C	C/T	C	C	C	C	C	C	C	C	
4425	G	G	C	C	C	C	C	C	C	C	C	
4477	A	A	A/G	A	A	A	A	A	A	A	A	**Intron 2**
4500-4501	DEL	DEL	G	DEL	G	G	G	G	G	G	G	
4505	G	G	A	A	A	A	A	A	A	A	A	
4526	T	T	C/T	C	T	T	T	C	C	C	T	
4528-4529	TT	TT	DEL	DEL	DEL	DEL	TT	DEL	DEL	T-	DEL	
4535	C	C	C	C	C	C	T	C	C	T	DEL	
4809	T	A	T	T	T	T	A	T	T	T	T	
4848	C	C	G/C	C	C	C	C	C	C	G	G	
4876	T	T	T/C	T	T	T	C	T	C	T	T	
4901	C	C	C	C	A/C	C	C	C	C	C	C	
4984	T	T	T/C	T	T	T	T	T	T	T	T	
5040	T	T	T/C	T	T	T	T	T	T	T	T	
5292-5301	AGTACAAA<br>CT	AGTACAAAC<br>T	AGTACAAACT / DEL	AGTACAAACT	AGTACAAACT	AGTACAAACT	DEL	AGTACAAACT	AGTACAAACT	AGTACAAACT	AGTACGA<br>ACT	
5361	T	T	C	T	T	T	C	T	T	T	T	
5414	T	T	C	T	T	T	C	T	T	T	T	
5417	T	T	G	T	T	T	G	T	T	G	G	
5428-5429	DEL	DEL	G	G	G	G	G	G	G	G	G	
5499	G	G	A	A	A	A	A	A	A	A	A	
5516	G	G	A	G	A	A	A	A	A	A	A	
5516-5517	DEL	DEL	GGA	GGA	GGA	GGA	GGA	GGA	GGA	GGA	GGA	
5527	C	C	DEL	DEL	DEL	DEL	DEL	DEL	DEL	DEL	DEL	
5558	A	A	G	A	A	A	A	A	G	G	G	
5573	A	A	A/G	A	A	A	A	A	G	A	G	
5578	C	C	T	C	C	C	T	C	T	T	T	
5605	T	T	C	T	T	T	T	T	C	T	C	
5624-5625	DEL	DEL	A	A	A	A	A	A	A	A	A	
5627	A	A	C	A	A	A	C	A	C	C	C	
5628	T	T	T	T	A	T	T	A	T	T	T	
5657	A	A	A/G	A	A	A	A	A	A	A	A	
5660	G	G	T	G	G	G	G	G	T	T	T	
5715	G	G	G/A	G	G	G	A	G	A	G	G	
5729-5730	DEL	DEL	DEL/GC	DEL	DEL	DEL	DEL	DEL	GC	DEL	DEL	**Intron 4**
5746	C	C	T/C	C	C	C	C	C	C	C	C	
5859	G	G	G/C	G	G	G	G	G	G	G	C	
5865	A	A	T/A	A	A	A	A	A	A	A	A	
5917	C	C	A	C	C	C	C	C	C	C	C	
5929	G	G	A	G	G	G	G	G	G	A	A	
5979	T	T	T/C	T	T	T	T	T	C	T	T	
5981	A	A	C	A	A	A	A	A	C	A	C	
5983	G	G	A	G	G	G	G	G	A	G	G	
6022	A	A	A	A	A/G	A	G	G	A	A	A	
6058	A	G	A/G	A	A	A	G	G	A	A	A	
6068	C	C	C	C	C/T	C	T	T	C	C	C	
6076	A	A	A	A	A/T	A	T	T	A	A	A	
6150	C	T	T	T	T	T	T	T	T	T	T	
6231	A	A	A/G	A	A	A	A	A	A	A	A	
6238	T	C	T	T	T	T	T	T	T	T	T	
6379	G	G	G/C	G	G	G	G	G	G	G	G	
6406-6407	DEL	DEL	G	G	G	G	G	G	G	G	G	
6440	A	A	G	A	A	A	G	A	G	G	G	
6468	G	G	A	G	G	G	A	G	G	G	A	
6473	C	C	DEL	DEL	DEL	DEL	DEL	DEL	DEL	DEL	DEL	
6481	C	A	A	A	A	A	A	A	A	A	A	
6570	T	T	C	T	T	T	C	T	C	T	C	
6582	C	C	DEL	DEL	DEL	DEL	DEL	DEL	DEL	DEL	DEL	
6786	C	C	C/T	C	C	C	C	C	C	C	C	

6844	C	C	A	C	C	C	A	C	C	A	A	
6951	C	T	T	T	T	T	T	T	T	T	T	
6973	G	G	G	G	G	G	A	G	G	A	G	
7081	G	A	A	A	A	A	A	A	A	A	A	
7096	T	T	C	T	T	T	C	T	C	C	C	
7115	T	T	C	T	T	T	C	T	C	C	C	
7116	G	G	A	G	G	G	A	G	A	A	G	**Intron 5**
7136	G	G	G/A	G	G	G	G	G	G	G	G	
7154-7177	14 T	13 T	11/15 T	14 T	13 T	15 T	12T	11T	12 T	11 T	10 T	
7314	G	G	A	A	A	A	A	A	A	A	A	
7340	G	G	G	G	G/T	G	G	G	G	G	G	
7462	C	C	C	C	T	T	C	T	T	T	T	
7523-7524	DEL	DEL	A	A	A	A	A	A	A	A	A	
7538	G	G	G	A	G	A	G	G	G	G	G	
7612	T	T	C	T	T	T	C	T	C	C	C	
7665	C	C	C	C	C	C	C	C	T	C	C	**Intron 6**
7724-7725	DEL	DEL	C	C	DEL	DEL	C	DEL	C	C	C	
7974	T	T	T/C	T	T	T	T	T	T	T	T	
7989	C	C	T/C	C	C	C	C	C	C	C	C	
8134	T	T	T/C	T	T	T	T	T	T	T	T	
8302	C	C	C	C	C	C	C	C	T	C	T	
8384	G	G	G/A	G	G	G	G	G	G	G	G	
8683	G (Gln89)	G (Gln89)	G (Gln89)	G (Gln89)	G (Gln89)	G (Gln89)	A (Gln89)	G (Gln89)	G (Gln89)	G (Gln89)	G (Gln89)	
8759	C (Pro115)	C (Pro115)	C (Pro115)	C (Pro115)	C (Pro115)	C (Pro115)	C (Pro115)	C (Pro115)	T (Ser115)	C (Pro115)	C (Pro115)	
8859	C (Pro148)	C (Pro148)	C (Pro148)	C (Pro148)	C (Pro148)	C (Pro148)	T (Leu148)	C (Pro148)	C (Pro148)	C (Pro148)	C (Pro148)	
8900	C (Val162)	C (Val162)	C (Val162)	C (Val162)	C (Val162)	C (Val162)	C (Val162)	C (Val162)	C (Val162)	G (Leu162)	C (Val162)	**Exon 7**
8915	C	T (Stop codon167)	C	C	C	C	C	C	C	C	C	
8946	C (Ala177)	C (Ala177)	C (Ala177)	C (Ala177)	T (Val177)	T (Val177)	C (Ala177)	T (Val177)	C (Ala177)	T (Val177)	C (Ala177)	
9080	T	T	T	T	T	T	T	T	C	T	T	
9110-9111	DEL	DEL	A	A	A	A	DEL	A	A	DEL	A	
9230	T	T	C	C	C	C	C	C	C	C	C	
9374	G	G	G/T	T	G	G	G	G	G	G	G	**Intron 7**
9390	C	C	C/T	C	C	C	C	C	C	C	T	
9456	A	A	A/T	A	A	A	A	A	A	A	A	
9486	C	C	A	A	C	C	A	C	C	A	A	
9701	C	C	DEL	DEL	DEL	DEL	DEL	DEL	DEL	DEL	DEL	
9773	G	G	A	G	G	G	G	G	A	G	G	
9885-9886	DEL	DEL	A	A	A	A	A	A	A	A	A	
9897-9898	DEL	DEL	TC	TC	TC	TC	TC	TC	TC	TC	TC	
9898	C	C	T	C	T	T	T	T	T	T	T	
9918-9919	DEL	DEL	T	T	T	T	T	T	T	T	T	
9936	G	G	T	T	T	T	T	T	T	T	T	
9944-9945	DEL	DEL	AAC	AAC	AAC	AAC	AAC	AAC	AAC	AAC	AAC	**Intron 8**
9946	C	C	A	C	A	A	A	A	A	A	A	
9961-9962	DEL	DEL	T	T	T	T	T	T	T	T	T	
10140	T	T	T	T	T	T	T	T	A	T	T	
10158	A	A	T	T	A	A	A	A	T	A	T	
10179	G	G	G	A	G	A	G	G	G	G	G	
10248	T	T	C	C	T	T	T	T	C	T	C	
10323	G	G	DEL	DEL	DEL	DEL	DEL	DEL	DEL	DEL	DEL	
10562	C	C	C	T	C	T	C	C	C	C	C	**Exon 9**

Supplementary Table S3. Coding and non-coding polymorphisms identified at the *CSN2 locus* across representative caprine alleles and related taxa. Nucleotide states are reported relative to the *Capra hircus* reference sequence AJ011018.3.
