# Supplementary material for: The Bovidae CSN2 Locus as a Multilayered Evolutionary System: Evidence from Retroposons, Trans-Species Variation and Ancient Recombination
Source: Animals (Basel). 2026 Aug 10;16(16):2482. doi: 10.3390/ani16162482 (PMC13508899; doi:10.3390/ani16162482)
Supplement: Supplementary file 1 [file animals-16-02482-s001.zip › Supplementary Table S4.pdf]

Species	SRA ID	Know polymorphisms				*CSN2* genotypes	Novel SNPs			
		**Exon 2 g.3794A>C p.Leu-2>Ile**	**Exon 7 g.8946T>C p.Val177>Ala**	**Exon 9 g.10562C>T 3' UTR**	**Indel intron 4 g.5292_5301**		**Exon 7 g.8683G>A p.90Gln**	**Exon 7 g.8759C>T p.Pro115>Ser**	**Exon 7 g.8859C>T p.Pro148>Leu**	**Exon 7 g.8900G>C p.Val162>Leu**
C. aegagrus	ERR219211	A/A	T/T	C/C	+/+	C/C	G/G	C/C	C/C	G/G
	SRR5803211	A/A	C/T	C/C	+/-	A/C	G/G	C/C	C/C	G/G
	SRR1576679	A/A	C/T	C/C	+/-	A/C	G/G	C/T	C/C	G/G
	ERR470106	A/A	T/T	C/C	+/+	C/C	G/G	C/C	C/C	G/G
	ERR470104	A/A	T/T	C/C	+/+	C/C	G/G	C/C	C/C	G/G
	ERR470100	A/A	C/C	C/C	-/-	A/A	G/G	C/C	C/C	G/G
	ERR340426	A/C	C/T	C/T	+/-	A1/C or A/C1 (ambiguous phase)		C/C	C/C	G/G
	ERR340347	A/A	C/T	C/C	+/-	A/C	G/G	C/C	C/C	G/G
	ERR340345	A/C	C/T	C/T	+/-	A1/C or A/C1 (ambiguous phase)	G/G	C/C	C/C	G/G
	ERR340344	A/A	C/T	C/C	+/-	A/C	G/G	C/C	C/C	G/G
	ERR340343	A/C	C/T	C/T	+/-	A1/C or A/C1 (ambiguous phase)	G/G	C/C	C/C	G/G
	ERR340342	A/A	C/C	C/C	+/-	A/A	G/G	C/C	C/C	G/G
	ERR340340	A/A	T/T	C/C	+/+	C/C	G/G	C/C	C/C	G/G
	ERR340338	A/A	T/T	C/C	+/+	C/C	G/G	C/C	C/C	G/G
	ERR340336	A/A	T/T	C/C	+/+	C/C	G/G	C/C	C/C	G/G
	ERR340335	A/A	C/T	C/C	+/-	A/C	G/G	C/C	C/C	G/G
	ERR340334	A/A	T/T	C/C	+/+	C/C	G/G	C/C	C/C	G/G
	ERR340333	A/A	C/C	C/C	-/-	A/A	G/G	C/C	C/C	G/G
	ERR340331	A/A	C/T	C/C	+/-	A/C	G/G	C/C	C/C	G/G
	ERR340330	A/A	T/T	C/C	+/+	C/C	G/G	C/C	C/C	G/G
	ERR340329	A/A	C/T	C/C	+/-	A/C	G/G	C/C	C/C	G/G
	ERR340328	A/A	T/T	C/C	+/+	C/C	G/G	C/C	C/C	G/G
	ERR340348	A/A	C/T	C/T	+/-	A/C	G/G	C/C	C/C	G/G
C. falconeri	SRR8437778	C/C	C/C	C/C	-/-	A2/A2	G/G	C/C	C/C	G/G
	SRR6246090	C/C	C/T	C/C	+/-	A2/CX	G/G	C/C	C/C	G/G
	SRR6246089	C/C	C/T	C/C	+/-	A2/CX	G/G	C/C	C/C	G/G
C. sibirica	SRR5258690	C/C	T/T	C/C	+/+	CX/CX	G/G	C/C	C/T	G/C
	SRR5803192	C/C	T/T	C/C	+/+	CX/CX	G/G	C/C	C/C	G/C
	SRR5803193	C/C	T/T	C/C	+/+	CX/CX	G/G	C/C	C/C	G/G
C. nubiana	SRR8437789	A/A	T/T	C/C	-/-	C/C	A/A	C/C	C/T	G/G
	SRR8437792	C/C	C/T	C/C	+/+	A2/CX	A/G	C/C	C/T	G/G
C. pyrenaica	SRR8437785	C/C	C/C	C/C	+/+	A2/A2	G/G	C/C	T/T	G/G
	SRR8437790	C/C	C/C	C/C	+/+	A2/A2	G/G	C/C	T/T	G/G

(A)

Species	SRA ID	Exon 2<br>g.3794A>C<br>p.Leu-2>Ile	Exon 7<br>g.8946T>C<br>p.Val177>Ala	Exon 9<br>g.10562C>T<br>3' UTR	*CSN2* genotypes	Breed	Country
C. hircus	SRR5803155	A/A	C/T	C/C	A/C	**Black**	**Bengal**
	SRR5803153	A/A	C/T	C/C	A/C	**Bengal goat**	
	SRR3144618	A/A	C/C	C/C	A/A	**Boer**	**Europe**
	SRX1560749	A/A	C/C	C/C	A/A		
	SRR5803174	A/A	C/T	C/C	A/C	**Poitou goat**	**France**
	SRR5803234	A/A	T/T	C/C	C/C	**Dries**	**Netherlands**
	SRR5803227	C/C	C/T	C/T	A2/C1 or A1/CX (ambiguous phase)	**Terry**	
	SRR5803226	A/A	C/C	C/C	A/A	**Saanen**	
	SRR5803152	A/A	C/T	C/C	A/C	**Beetal Black**	**Pakistan**
	SRR5803151	A/A	C/C	C/C	A/A		
	SRR5803148	A/A	C/T	C/C	A/C		
	SRR5803150	A/A	C/C	C/C	A/A	**Beetal Brown**	
	SRR5803149	A/A	C/C	C/C	A/A	**Barbari**	
	SRR5803147	A/A	C/T	C/C	A/C	**Beetal**	
	SRR17775431	A/A	C/T	C/C	A/C	**Local population**	
	SRR17775432	A/C	C/T	C/C	C/A2 or CX/A (ambiguous phase)		
	SRR17775434	A/A	T/T	C/C	C/C		
	SRR17775435	A/A	C/T	C/C	A/C		
	SRR17775444	A/A	C/C	C/C	A/A		
	SRR17775461	A/A	C/C	C/C	A/A		
	SRR17775462	A/A	C/T	C/C	A/C		
	SRR17775463	A/A	C/C	C/C	A/A		
	SRR17775464	A/A	C/C	C/C	A/A		
	SRR17775465	A/A	C/C	C/C	A/A		
	SRR17775466	A/A	T/T	C/C	C/C		
	SRR17775467	A/A	C/C	C/C	A/A		
	SRR17775468	A/A	C/T	C/C	A/C		
	SRR17775469	A/A	C/T	C/C	A/C		
	SRR17775470	A/A	T/T	C/C	C/C		
	SRR17775472	A/A	T/T	C/C	C/C		
	SRR17775473	A/A	C/T	C/C	A/C		
	SRR17775474	A/A	C/T	C/C	A/C		
	SRR17775475	A/A	T/T	C/C	C/C		
	SRR17775476	A/A	T/T	C/C	C/C		
	SRR17775477	A/A	C/T	C/C	A/C		
	SRR3144623	C/C	T/T	T/T	C1/C1	**Coppernecked**	**Switzerland**
	SRR3144630	C/C	T/T	T/T	C1/C1	**Saanen**	
	SRR3144625	A/A	C/C	C/C	A/A		
	SRR3144624	A/A	C/C	C/C	A/A		
	SRR3144633	A/C	C/T	C/T	A1/C or A/C1		

					(ambiguous phase)		
	SRR3144601	C/C	T/T	T/T	C1/C1	**Grisons Striped**	
	SRR3144619	A/C	T/T	C/C	C/CX	**Tessin Grey**	
	SRR3144621	A/C	C/T	C/T	A1/C or A/C1 (ambiguous phase)	**Chamois Colored**	
	SRR3144622	A/C	C/T	C/T	A1/C or A/C1 (ambiguous phase)		
	ERR470102	A/A	C/C	C/C	A/A	**Saanen**	**France**
	ERR470101	A/A	C/T	C/C	A/C		
	ERR470105	A/A	T/T	C/C	C/C	**Alpine**	
	ERR470103	A/A	C/T	C/C	A/C		
	SRR5803235	A/A	T/T	C/C	C/C		
	ERR340339	A/A	T/T	C/C	C/C	**local breed**	**Iran**
	ERR340337	A/A	C/T	C/C	A/C		
	ERR340332	A/A	T/T	C/C	C/C		
	ERR313215	A/A	C/T	C/C	A/C		
	ERR313213	A/A	T/T	C/C	C/C		
	ERR313212	A/A	T/T	C/C	C/C		
	ERR313211	A/A	T/T	C/C	C/C		
	ERR313210	A/C	T/T	C/C	C/CX		
	ERR313209	A/C	T/T	C/C	C/CX		
	ERR313207	A/A	T/T	C/C	C/C		
	ERR313206	A/A	C/T	C/C	A/C		
	ERR313204	A/C	T/T	C/C	C/CX		
	ERR313202	A/A	T/T	C/C	C/C		
	ERR313200	A/C	C/T	C/C	C/A2 or CX/A (ambiguous phase)		
	ERR313199	A/A	T/T	C/C	C/C		
	ERR313198	A/A	C/T	C/C	A/C		
	ERR313197	A/A	C/C	C/C	A/A		
	ERR299456	A/A	C/T	C/C	A/C		
	ERR299449	A/A	C/T	C/C	A/C		
	ERR297229	A/A	C/T	C/C	A/C		
	SRR4051866	A/A	T/T	C/C	C/C	**Inner Mongolia cashmere**	**China**
	SRR4051964	A/C	T/T	C/T	C/C1		
	SRR4051928	A/A	T/T	C/C	C/C		
	SRR4052103	A/A	C/T	C/C	A/C		
	SRR4052272	A/A	C/T	C/C	A/C		
	SRR4052346	A/A	C/T	C/C	A/C		
	SRR4052349	A/A	T/T	C/C	C/C		
	ERR313259	A/A	C/C	C/C	A/A	**Local population**	**Morocco**
	ERR313264	A/C	T/T	C/T	C/C1		
	ERR315501	A/A	C/C	C/C	A/A		
	ERR315503	A/A	T/T	C/C	C/C		
	ERR315504	A/A	C/C	C/C	A/A		
	ERR315506	A/A	C/T	C/C	A/C		
	ERR315511	A/A	C/C	C/C	A/A		

	ERR345977	A/A	C/C	C/C	A/A		
	SRR4064255	A/A	C/T	C/C	A/C	**Liaoning cashmere**	**China**
	SRR4064259	A/A	C/T	C/C	A/C		
	SRR4064259	A/A	C/T	C/C	A/C		
	SRR4068058	A/A	C/T	C/C	A/C		
	SRR4068057	A/A	T/T	C/C	C/C		
	SRR4068056	A/A	C/C	C/C	A/A		
	SRR1999390	A/C	C/T	C/C	C/A2 or CX/A (ambiguous phase)	**Tibetan**	
	SRR1999396	A/A	C/T	C/C	A/C		
	SRR1999407	A/C	C/T	C/C	C/A2 or CX/A (ambiguous phase)		
	SRR1999408	A/A	T/T	C/C	C/C		
	SRR1999409	A/A	C/T	C/C	A/C		
	SRR1999410	A/A	C/T	C/C	A/C		
	SRR1999411	A/C	T/T	C/C	C/CX		
	SRR1999413	A/A	T/T	C/C	C/C		
	SRR1999415	A/A	C/T	C/C	A/C		
	SRR1999416	A/A	T/T	C/C	C/C		
	SRR1999419	A/A	T/T	C/C	C/C		
	SRR1999477	A/A	T/T	C/C	C/C		
	SRR1999645	A/A	T/T	C/C	C/C		
	SRR1999646	A/A	T/T	C/C	C/C		
	SRR5149617	A/A	C/C	C/C	A/A		

(B)

Supplementary Table S4. Detailed genotypic characterization of *CSN2* polymorphisms identified from public SRA (Sequence Read Archive) datasets across wild (**A**) and domestic goats (*Capra hircus*) (**B**) taxa. The table includes known polymorphisms, novel SNPs, intronic insertion/deletion states, inferred haplotypes, breed information, and sequence accession identifiers.
