# Supplementary material for: The Bovidae CSN2 Locus as a Multilayered Evolutionary System: Evidence from Retroposons, Trans-Species Variation and Ancient Recombination
Source: Animals (Basel). 2026 Aug 10;16(16):2482. doi: 10.3390/ani16162482 (PMC13508899; doi:10.3390/ani16162482)
Supplement: Supplementary file 1 [file animals-16-02482-s001.zip › Supplementary Table S5.pdf]

10 bp insertion sequence variation across mammals

		Position									
**Species**	**Clade**	**1**	**2**	**3**	**4**	**5**	**6**	**7**	**8**	**9**	**10**
**Consensus**		A	G	T	A	C	A	A	A	C	T
Antilocapra americana	Antilocapridae	A	G	T	A	C	G	A	A	C	T
Aepyceros melampus	Antilopinae	A	G	T	A	C	G	A	A	C	T
Eudorcas thomsonii	Antilopinae	A	G	T	A	C	G	A	C	C	T
Gazella	Antilopinae	A	G	T	A	C	G	A	A	C	T
Litocranius walleri	Antilopinae	A	G	T	A	C	G	A	A	C	T
Nanger dama	Antilopinae	A	G	T	A	G	G	A	A	C	T
Saiga tatarica	Antilopinae	A	G	T	A	C	G	A	A	C	T
Bison bison	Bovinae	A	G	T	A	C	A	A	A	C	T
Bos frontalis	Bovinae	A	G	T	A	C	A	A	A	C	T
Bos grunniens	Bovinae	A	G	T	A	C	A	A	A	C	T
Bos indicus	Bovinae	A	G	T	A	C	A	A	A	C	T
Bos javanicus	Bovinae	A	G	T	A	C	A	A	A	C	T
Bos motus	Bovinae	A	G	T	A	C	A	A	A	C	T
Bos taurus	Bovinae	A	G	T	A	C	A	A	A	C	T
Bubalus arnee	Bovinae	A	G	T	A	C	A	A	A	C	T
Bubalus bubalis	Bovinae	A	G	T	A	C	A	A	A	C	T
Syncerus caffer	Bovinae	A	G	T	A	C	A	A	A	C	T
Camelus dromedarius	Camelidae	G	G	T	A	C	A	A	A	C	T
Budorcas taxicolor	Caprinae	A	G	T	A	C	G	A	A	C	T
C. hircus breed Xinong Saanen	Caprinae	A	A	T	A	C	A	A	A	C	T
C. nubiana	Caprinae	A	A	T	A	C	A	A	A	C	T
Capra aegagrus	Caprinae	A	G	T	A	C	A	A	A	C	T
Capra hircus	Caprinae	A	G	T	A	C	A	A	A	C	T
Capra ibex	Caprinae	A	G	T	A	C	A	A	A	C	T
Ovibos moschatus	Caprinae	A	G	T	A	C	A	A	A	C	T
Ovis aries	Caprinae	A	G	T	A	C	G	A	A	C	T
Ovis vignei	Caprinae	A	G	T	A	C	G	A	A	C	T
Pseudois nayaur	Caprinae	A	G	T	A	C	G	A	A	C	T
Rupicapra rupicapra	Caprinae	A	G	T	A	C	G	A	A	C	T
Capricornis	Caprinae/Cervid-like	A	G	T	A	T	G	A	A	C	T
Ovibos moschatus (alt)	Caprinae/Cervid-like	A	G	T	A	T	G	A	A	C	T
Alces alces	Cervidae	A	G	T	A	T	G	A	A	C	T
Capreolus capreolus	Cervidae	A	G	T	A	T	G	A	A	C	T
Cervus nippon	Cervidae	A	G	T	A	T	G	A	A	C	T
Muntiacus reevesi	Cervidae	A	G	T	A	G	G	A	A	C	T
Rangifer tarandus	Cervidae	A	G	T	A	T	G	A	A	C	T
Pipistrellus pipistrellus	Chiroptera	G	C	T	A	C	A	A	A	T	T
Giraffa	Giraffidae	A	G	T	A	C	A	A	A	C	T
Hexaprotodon liberiensis	Hippopotamidae	G	G	T	A	C	A	A	A	C	C
Hippopotamus amphibius	Hippopotamidae	G	G	T	A	C	A	A	A	C	C
Oryx dammah	Hippotraginae	A	G	T	A	C	G	A	A	C	T
Oryx gazella	Hippotraginae	A	G	T	A	C	G	A	A	C	T
Neotragus moschatus	Neotraginae	A	G	T	A	C	G	A	G	C	T
Antilope tibetana	Pantholopini	A	G	T	A	C	A	A	A	C	T
Pantholops hodgsonii	Pantholopini	A	G	T	A	C	G	A	A	C	T
Ceratotherium simum simum	Perissodactyla	G	G	T	G	C	A	A	A	C	T
Equus asinus	Perissodactyla	G	G	T	A	C	A	A	G	C	T
Homo sapiens	Primates	G	G	T	A	C	A	A	A	C	T
Kobus ellipsiprymnus	Reduncinae	A	G	T	A	C	A	A	A	C	T
Redunca redunca	Reduncinae	A	G	T	A	C	A	A	A	C	T
Sus scrofa	Suidae	G	G	T	A	C	A	A	A	C	T
Tragulus javanicus	Tragulidae	A	G	T	A	C	A	A	A	C	T

Supplementary Table S5. Comparative matrix of nucleotide variation within the conserved 10 bp insertion located in intron 4 of the *CSN2 locus* across representative mammalian taxa. Columns represent nucleotide positions within the insertion, whereas rows correspond to species grouped according to phylogenetic affinity. The consensus sequence (AGTACAAACT) is shown at the top. Grey shading indicates nucleotide states differing from the consensus sequence. Only taxa retaining the orthologous insertion state were included in the comparison.
